# Permutation test applied to lexical reconstructions partially supports the Altaic linguistic macrofamily

**DOI:** 10.1017/ehs.2021.28

**Published:** 2021-06-01

**Authors:** Alexei S. Kassian, George Starostin, Ilya M. Egorov, Ekaterina S. Logunova, Anna V. Dybo

**Affiliations:** 1 School of Advanced Studies in the Humanities, The Russian Presidential Academy of National Economy and Public Administration, Moscow, Russia; 2 Institute for Oriental and Classical Studies, National Research University Higher School of Economics, Moscow, Russia; 3 Santa Fe Institute, New Mexico, USA; 4 Institute of Linguistics, Russian State University for the Humanities, Moscow, Russia; 5 Institute of Linguistics, Russian Academy of Sciences, Moscow, Russia

**Keywords:** Altaic languages, Transeurasian languages, language classification, permutation test, Altaic hypothesis

## Abstract

In this paper, we present the results of our analysis of the 110-item basic wordlists for four reconstructed and one ancient languages, the linguistic ancestors of five language families which are hypothesized to constitute the Altaic (a.k.a. Transeurasian) macrofamily: Proto-Turkic, Proto-Mongolic, Proto-Tungusic, Middle Korean and Proto-Japonic wordlists. Protolanguage wordlists were reconstructed according to strict criteria of semantic reconstruction, based on accurate semantic glossing of forms in daughter languages. Each involved form was encoded into a bi-consonantal *CC*-shaped sequence using the consonant class method, after which a recently developed weighted permutation test was applied. In a typical situation, our algorithm makes a small number of type 1 errors (false positive), but the number of type 2 errors (false negative) can be substantial. Our main finding is that pairs between the Nuclear Altaic taxa – Turkic, Mongolic and Tungusic – as well as the Turkic-Japonic and Tungusic-Japonic pairs demonstrate significant *p*-values. In some cases, this can be attributed to either ancient contacts or genealogical relationships, but at least for the Turkic–Japonic pair, a contact scenario is unlikely owing to geographical remoteness.

## Graphical abstract

**Figure:**
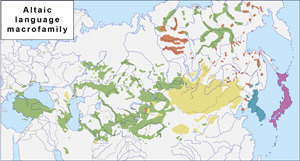

**Social media summary:** The paper finds statistical support for a common origin in the basic lexicon of five families commonly known as Altaic

## Introduction

**Figure 1.**
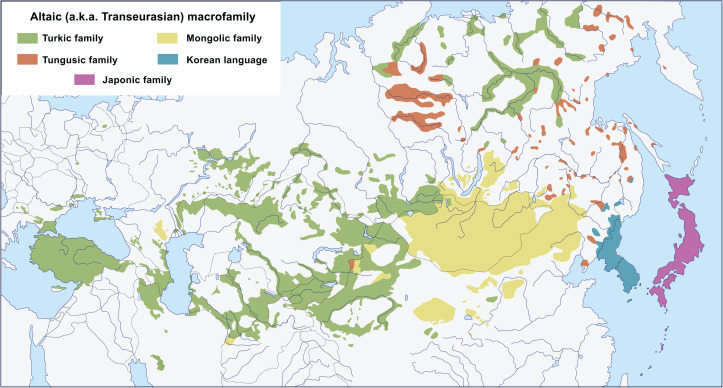
Modern distribution of the five language families that constitute the Altaic (a.k.a. Transeurasian) macrofamily.

The so-called Altaic hypothesis suggests common ancestry for several universally accepted language families spoken across Eurasia, namely the Turkic, Mongolic, Tungusic, Korean and Japonic families (see Fig. 1 for the map). The hypothesis has two common versions. The ‘narrow’ one claims that Turkic, Mongolic and Tungusic are genealogically related to each other and form a distinct clade. The ‘broad’ version expands this clade to also include Korean and Japonic. The resulting clade is traditionally labelled as the Altaic macrofamily, although certain researchers use the term ‘Altaic macrofamily’ specifically for the [Turkic, Mongolic, Tungusic] clade, whereas the same plus Korean and Japonic can be referred to as the ‘Macro-Altaic’ or, using a recently introduced term, ‘Transeurasian macrofamily’. Below we prefer to keep the term ‘Altaic’ for the Altaic macrofamily in the broad sense, assigning the special term ‘Nuclear Altaic’ to the [Turkic, Mongolic, Tungusic] clade.

A very detailed and extensive overview of the Altaic hypothesis, its history and the current state of art is offered in Blažek (2019), with the recent handbook (Robbeets & Savelyev, 2020) nicely complementing it. As of today, the only attempt at a systematic reconstruction of Proto-Altaic (= Macro-Altaic) phonology and vocabulary has been offered in Starostin, Dybo, & Mudrak (2003); however, it has been severely criticized for various perceived methodological flaws by both opponents of the Altaic hypothesis (e.g. Vovin, 2005) and its supporters (e.g. Robbeets, 2005, offering a major revision of the reconstructed phonology and rejecting a large number of proposed Altaic etymologies).

In general, most experts agree that lexical similarities between the ‘narrow’ Altaic taxa – Turkic, Mongolic and Tungusic – are too numerous to be accidental, but the proposed historical scenarios differ: some scholars argue in favour of genealogical relationship, while others attribute the observed lexical matches to several chronological layers of (prehistoric) contacts. The same arguments are applicable to the relations between Korean and Japonic, while their hypothetical connections to Turkic, Mongolic and Tungusic are less obvious and actually raise the issue of potential chance resemblances.

A complicated, but historically realistic, ‘hybrid scenario’ (usually adopted by ‘Altaicists’, albeit to highly differing degrees) could imply that an original deep-level genealogical relationship between Turkic, Mongolic, Tungusic, Korean and Japonic may have later become overlapped and partially obscured by various contacts between the already separated branches, varying in intensity and continuance.

As far as we know, the only previously published statistical tests for Altaic relatedness are Turchin, Peiros and Gell-Mann (2010) and Ceolin (2019). The first of these studies (Turchin et al., 2010) applies a bootstrap test to the data, consisting of 100-item wordlists encoded for analysis in accordance with the so-called ‘consonant class’ method (in which each form is reduced to a ‘consonantal type’ based on its two first consonants). The languages involved include Proto-Turkic, Proto-Mongolic, Proto-Tungusic and Proto-Japonic (no Korean). However, the analysis suffers from several shortcomings, such as:

1. There is no methodology of semantic (onomasiological) reconstruction. Protoforms and their meanings were taken from Starostin et al. (2003) and other databases of the Tower of Babel Project, although precise semantic reconstruction was never a goal of the project and individual databases can seriously vary in quality in respect to semantic accuracy.
2. The accepted allocation of consonants to classes seems unreasonably coarse-grained: only nine consonant classes are introduced and some decisions look controversial, e.g. *r*-type phonemes are united with *t*-type phonemes as a single class, even though it is typologically more natural to either join *r*- and *l*-type phonemes in a single class or (as we prefer) assign a distinct class for *r*-type sounds.
3. No multiple testing correction was applied to the results of individual bilateral comparisons.

Despite these points, Turchin et al.'s experiment is somewhat similar to the one proposed in the present paper.

A recent paper (Ceolin, 2019) represents a completely different paradigm. The dataset includes 168-item wordlists from three standardized languages, representative of the three Nuclear Altaic families: Literary Turkish, Standard Khalkha Mongolian and Written Manchu. The author attempts to revive some archaic techniques of machine wordlist comparison previously proposed by other scholars (Ringe, Kessler and Lehtonen), e.g. for Indo-European–Uralic comparison. The flaws of Ceolin's experiment appear to be exactly the same as those of the preceding IE-Uralic attempts overviewed and criticized in Baxter (1998) and Kassian, Starostin and Zhivlov (2015: 302–304). The following are the most important objections:

1. The choice of automatic algorithms applied to the data is not substantiated by the author; moreover, it explicitly contradicts the techniques normally used in comparative-historical linguistics. The main problem is that only the initial root consonant is taken into account. This is the same as if we were to investigate the language relationship between English and German by focusing on such lexical pairs with similar initial consonants as *belly/Bauch*, *bird/Vogel*, *woman/Frau*. In actual practice, historical linguists always attempt to compare *whole roots* rather than arbitrarily selected parts of roots. Because of this, and since the most common root pattern in the world's languages is *CVC(V)* (mono-consonantal structures *CV* and *VC* may formally be regarded as variants with a zero-class consonant in the first or second position – *CH* and *HC* respectively), the automated bi-consonantal comparison between *CC*-forms as proposed by Dolgopolsky (1964, 1986), then adapted by Turchin et al. (2010) and then by Kassian et al. (2015) and employed for the present study, is more appropriate for machine analysis when it comes to minimizing false positive matches.
2. As a result, the applied automatic algorithms reveal a substantial number of incorrect etymological pairs and improbable sound correspondences, e.g. within the Indo-European test group, Ceolin's (2019: 313) machine comparison between such recognizably related lects as Modern Italian and Standard Hindi recognizes the phantom correspondence ‘Italian *l-*/Hindi *c-*’ deduced from the following ‘etymologies’: It. *largo/*Hi. *caura* ‘wide’, It. *liscio/*Hi. *cikna* ‘smooth’, It. *luna/*Hi. *cand* ‘moon’ (not a single one of these pairs represents true cognates). Similarly odd sound correspondences can be found in Ceolin's (2019: 318) Altaic comparison. Thus, Turkish *k-/*Manchu *f-* involves such ‘etymologies’ as:
  - Turkish *kɨsa* ‘short’ (< Proto-Turkic *kɨs-ka ‘short’ from Proto-Turkic *kɨs- ‘to press, squeeze’; Dybo, 2013: 542) = Manchu *foxolon* ‘id.’ (< Proto-Tungusic *poko-lo- ‘short(?)’; Starostin et al., 2003: 1100; Tsintsius, 1977: 331);
  - Turkish *kök* ‘root’ (< Proto-Turkic *kök ‘a k. of root’; Dybo, 2013: 435) = Manchu *fulexe* ‘id.’ (Tsintsius, 1977: 302; no *comparanda* in Tungusic, but the Proto-Tungusic form is expected to be *pule-; Starostin et al., 2003: 1182), and so on. Likewise, Khalkha *nV-*/Manchu *V-*involves such ‘etymologies’ as:
  - Khalkha *neg* ‘one’ (< Proto-Mongolic *nike-n ~ *nige-n; Gruntov & Mazo, 2015: 235; Nugteren, 2011: 460) = Manchu *emu* ‘id.’ (< Proto-Tungusic *emu; Starostin et al., 2003: 505; Tsintsius, 1977: 270–272);
  - Khalkha *nawč* ‘leaf’ (< Proto-Mongolic *labči-n ~ *nabči-n; Gruntov & Mazo, 2015: 231; Nugteren, 2011: 450) = Manchu *abdaxa* ‘id.’ (< Proto-Tungusic *xabda-nsa; Starostin et al., 2003: 764; Tsintsius, 1975: 5), and so on.
  We are not aware of any version of Proto-Altaic reconstruction or of any proposed scenario of lexical contact that would imply that the forms within the aforementioned pairs could be related to each other. Given that these are but a few examples quoted from a large group of similarly false positives, it is not clear whether the obtained results could be considered trustworthy even from a purely theoretical stance.
3. The wordlists were manually designed in order to reinforce the signal, raising the issue of personal bias. Thus, the concept ‘man (male)’ was intentionally included in the list (Ceolin, 2019: 308) for the sake of the match between Turkish *er* ‘man (male)’ and Khalkha *er* ‘id.’, but at the same time the mismatched Manchu *xaxa* ‘id.’ was deleted from the dataset owing to its allegedly onomatopoeic nature (it is actually very much an open question how justified it is to automatically consider any *C*_1_*V*_1_*C*_1_*V*_1_-stem encountered in any of the world's languages as onomatopoeia).
4. Compiled wordlists suffer from inaccurate lexicographic work. Thus, e.g. within the Khalkha 110-item wordlist (a subset of the whole Khalkha 168-item wordlist used by Ceolin), the following errors have been detected, the majority of which directly affect Ceolin's test results:
  - ‘earth’ – Khalkha *towrog* actually means ‘dust’ and must be excluded. The basic Khalkha term *šoroː* ‘earth (soil)’ is not listed at all. This is crucial for the experiment since Khalkha *towrog* matches Turkish *toprak* ‘earth’ in terms of initial consonants;
  - ‘to hear’ – the rare Khalkha form *duːla-* specifically means ‘to heed, hearken’ and must be excluded. This is crucial for the experiment since Khalkha *duːla-* matches Turkish *duymak*, Manchu *donjimbi* ‘to hear’ in terms of initial consonants;
  - ‘horn’ – Khalkha *büreː* actually means ‘horn (music instrument)’ and must be excluded. This is crucial for the experiment since Khalkha *büreː* matches Turkish *boynuz* ‘horn’ in terms of initial consonants;
  - ‘root’ – the quoted Khalkha word *ug* ‘root’ is a marginal rather than basic term;
  - ‘short’ – Khalkha *axar ~ oxor* ‘short’ exists, but it is a marginal term with limited lexical compatibility, so it must be excluded;
  - ‘to sleep’ – the quoted Khalkha verb *noyrso-* ‘to sleep’ is a marginal rather than basic term;
  - ‘small’ – Khalkha *ǯaːl* actually means ‘baby; small (of age)’, *ǯiǯig* actually means ‘minute, petty (Russian *melkij*)’ rather than generic ‘small’, whereas the basic Khalkha term *očüːxen* ‘small (of physical objects)’ is not listed at all. This is crucial for the experiment since Khalkha *očüːxen* would match Manchu *ajige* ‘small’ in terms of initial consonants;
  - ‘year’ – quoted Khalkha *ǯil* means ‘annual cycle, calendar year’. A more basic term is *on* ‘year (as a time period)’. At the very least, both should be taken as synonyms. This is crucial for the experiment since Khalkha *on* would match Manchu *aniya* ‘year’ in terms of initial consonants.

Most importantly, it may be *a priori* questioned whether a permutation test conducted on three modern (or historically attested) literary languages, at least two of which (Khalkha and Manchu) have been in a state of intense linguistic contact with each other over the past several centuries, could yield a proper positive signal for a genetic relationship between their ancestors that is generally understood (by those who support the Altaic hypothesis) to date to a period preceding Indo-European. In our opinion, to stand at least a reasonable chance of success, such a test should be conducted not on a small number of living descendants, all of which have undergone significant cognate loss over millennia, but on the reconstructed ancestral stages of these languages – provided, of course, that the latter have been produced based on a more or less rigorous application of the classic comparative method.

The goal of our present project is to estimate the degree of statistical support for the Altaic hypothesis with the help of an algorithm which could, at least to a certain extent, emulate the research of an actual comparative linguist. It is the combination of such a research procedure, well tested over the past couple of centuries, with the formal edge provided by machine algorithms that could, in our opinion, provide the best possible outcome for any controversial hypotheses of distant linguistic relationship.

Throughout the text we largely use scientific terminology that is well established in basic comparative historical linguistics, but for the sake of those readers who are not well versed in this subject it makes sense to provide a few concise definitions for some of the most important terms. Two or more languages are considered to be *genealogically related* if they may be traced, through historic sources or comparative reconstruction, to a single (attested or hypothetical) ancestral language. Such common ancestry is demonstrated through sets of *cognates* – full words or single morphemes (roots, affixes, etc.) inherited by the compared languages from their ancestor. As a rule, cognacy of specific items is proven by establishing regular (recurrent) *sound correspondences* which are common across multiple sets of cognates rather than isolated ones, thus ruling out the possibility of accidental resemblance (e.g. English *t* – German *z*, as in *two* – *zwei*, *ten* – *zehn*, *to* – *zu*, etc.). Since a genealogic relationship of languages is relative rather than absolute (i.e. some languages may be more closely related to each other than others, just like biological species), there exist various methods to measure degrees of relationship; most of them are *lexicostatistical* in nature, using fixed lists of the so-called *basic vocabulary* (words that represent some of the most common and universal concepts, such as body parts, simple natural objects, essential vital functions, etc.) to determine the relative proximity between languages by the percentages of cognates on these lists. For living languages, basic vocabulary wordlists may be collected from text corpora or dictionaries, or directly from native speakers; for proto-languages, they have to be reconstructed by applying a special *onomasiological* methodology (choosing the optimal candidate for the corresponding meaning on the wordlist, e.g. *twa- for Germanic ‘two’ on the basis of English *two* and German *zwei*, etc.).

## Materials and methods

Below we present our analysis for five Swadesh 110-item wordlists which represent five branches constituting the hypothetical Altaic macrofamily: Turkic, Mongolic, Tungusic, Korean and Japonic (see (Kassian, Starostin, Dybo & Chernov, 2010) for the principles of Swadesh wordlist compilation and semantic specifications of Swadesh concepts).

Out of these five, four wordlists have been reconstructed: Proto-Turkic, Proto-Mongolic, Proto-Tungusic and Proto-Japonic. The fifth wordlist reflects the synchronic basic vocabulary of Late Middle Korean (owing to the relative historical irrelevance of data on most modern Korean dialects and to the scantiness of pre-Hankul data, it is hard to reconstruct a Swadesh list that would be equivalent to Old Korean or precede it, but the Late Middle Korean wordlist may be considered as a reasonable proxy).

For Proto-Japonic, we introduce two distinctly different versions of the wordlist, since there are some significant unresolved problems in its reconstruction where adhering to one or the other solution influences the results of testing – namely, the reconstruction of Proto-Japonic by Sergei Starostin (1991), later adopted for the Altaic etymological dictionary (Starostin et al., 2003), posits an initial *d- for Proto-Japonic, whereas a more conservative approach prefers the phonetic interpretation of the same phoneme as *y- (Martin, 1987, gives no preference to either approach; both Vovin, 2005, and Robbeets, 2005, explicitly reject *d-; see Supplementary Material for details). Ultimately, we have to perform two sets of calculations because of these differences in interpretation of Proto-Japonic phonology.

A similar uncertainty concerns the Proto-Turkic reconstruction, in which the phonemes *ŕ and *ĺ yield *r* and *l* in the Bulghar branch, but *z and *š in Nuclear (*scil*. ‘Common’) Turkic. The oldest view (shared by some modern Turkologists as well, e.g. Róna-Tas, 1998: 71–72; Shcherbak, 1994: 132–142) is to reconstruct these phonemes as *z, *š for Proto-Turkic. Later, however, this was contested in favour of an alternative interpretation of these phonemes as specific liquidae ‘*ŕ, *ĺ’ (e.g. Doerfer, 1968: 17, 1976: 30–36, 1988; Poppe, 1926: 107; Ramstedt, 1923: 29). We follow the second view and reconstruct the Proto-Turkic items in question as palatal *rʸ, *ʎ (a more phonetically exact reconstruction would be flap *ɾ or ‘Czech’ *r̝ and voiceless *ɬ), since we consider the evidence for such an interpretation to be conclusive; consequently, we have intentionally abstained from introducing an alternative Proto-Turkic wordlist with the phonemes *z and *š into our dataset. The main argument is the fact that Proto-Turkic *rʸ, *ʎ regularly correspond to Proto-Mongolic *r, *l, regardless of whether one accepts the Altaic hypothesis or treats the following examples as loanwords in one direction or another:

- PT *burʸagu ‘calf’ / PM *biraɣu ‘calf (1 year old)’;
- PT *borʸ ‘gray’ / PM *boro ‘grey’;
- PT *kumɨrʸ ‘fermented milk’ / PM *kimur ‘fermented milk with water’;
- PT *arʸɨg ‘fang’ / PM *ariɣa ‘molar’;

or

- PT *arɨʎ ‘thill’ / PM *aral ‘thill’;
- PT *aʎ ‘food’ / PM *öl ‘nutrition’;
- PT *köʎ- ‘to freeze’ / PM *köl-de- ‘to freeze’.

Similarly, lexical matches speaking in favour of *r-*like and *l-*like articulation of the Turkic phonemes in question can be found in Proto-Tungusic and Proto-Korean. Lexical borrowings between Proto-Turkic and such taxa as Old Chinese, Proto-Samoyedic and Proto-Yeniseian also unequivocally point to *r-*like and *l-*like sounds (Dybo, 2007). There is no reason to attribute these Turkic words to Proto-Bulghar rather than to Proto-Turkic (in fact, sometimes there are specific obstacles to such a scenario).

Specific phonetic arguments in favour of original liquids include, for instance, the development of Proto-Turkic clusters *Crʸ into Proto-Nuclear Turkic *Cr (Helimski, 1986; Tenishev & Dybo, 2006: 37–38). For example,

*gökürʸ ‘breast, chest’ → diminutive *gökr-ek;
*tɨgɨrʸ ‘firm, tough’ → *tɨgr-ak ‘id.’ (with regular reduction of the narrow vowel).

Under the ‘rhotacism’ reconstruction model, one should assume the development *kz > *kr for such cases, which would be phonetically unmotivated and typologically unusual.

Phonological and morphological reconstructions for Proto-Turkic, Proto-Mongolic and Proto-Tungusic have generally been adapted from Starostin et al. (2003), with further emendations and corrections discussed in the Supplementary Material, along with references. For the problem of selecting the optimal lexical candidate(s) for the reconstruction slot, we adhere to the principles of semantic (onomasiological) reconstruction proposed in Kassian et al. (2015: 305–306), namely, topological principle, external etymology principle, internal etymology principle, semantic plausibility principle and areal effect exclusion principle. Detailed linguistic comments on each reconstructed word are offered in the Supplementary Material.

We encoded the involved wordforms in the simplified consonant class-based transcription, reducing them to bi-consonantal *CC*-shaped skeletons. The entire transcriptional procedure is the same as previously described for the Indo-European–Uralic comparison (Kassian et al., 2015: 307–309). The general idea of consonant classes and the advantage of *CC*-transcription goes back to Dolgopolsky (1964, 1986). The specific consonant classes are listed in Table 1.

**Table 1.** Consonant classes

P-class (labials)	p b ɓ β f v …
T-class (dentals)	t d ɗ θ ð ȡ ȶ …
S-class (sibilant fricatives)	s z š ž …
Ʒ-class (sibilant affricates)	c ʒ č ǯ …
Y-class (palatal glides)	y …
W-class (labial glides)	w ʍ …
M-class (labial nasals)	m ɱ …
N-class (non-labial nasals)	n ɳ ɲ ŋ …
Q-class (lateral affricates)	ƛ …
R-class	r ɾ …
L-class	l ɭ ɬ ɫ …
K-class (velars and uvulars)	k g x ɣ q χ ʁ …
Zero-class or H-class	ħ ʕ ʜ ʢ ʡ h ɦ ʔ and any vowels

A weighted permutation test was applied to each pair within the five involved wordlists, 10 pairs in total. The permutation test algorithm marks a pair of forms with the same *CC*-transcription as a match (*CC*-match), and other pairs as non-matching. The method of consonant classes is thus, on the one hand, a crude variation on the measurement of Levenshtein distances and, on the other hand, is close to modelling the preliminary stage of real comparative-historical research, at least as far as criteria for eliciting potential etymological lexical matches between two languages are concerned. The weighted permutation test was recently proposed in Starostin et al. (forthcoming); its difference from the standard (unweighted) test (described, e.g., in Kassian et al., 2015: 309–310) is that the typological stability of individual Swadesh concepts is taken into account: coincidence within a more stable concept is treated as more expensive than coincidence within a less stable concept. One million pseudo-random trials were performed.

Some examples for *CC-*matches between Proto-Mongolic and Proto-Tungusic wordlists are:

- ‘green’ – Mongolic *nogoha = Tungusic *ɲog = consonant classes *NK*;
- ‘I’ – Mongolic *bi = Tungusic *bi = consonant classes *PH*;
- ‘stone’ – Mongolic *čila = Tungusic *ǯolo = consonant classes *ƷL*.

See Supplementary Material for all detected *CC* matches between the Altaic wordlists.

## Results

Probabilistic results of pairwise comparison between five Altaic wordlists are offered in Tables 2 and 3. The Holm–Bonferroni correction (Holm, 1979) was applied to counteract the problem of multiple comparisons within each of two sets of wordlists.

**Table 2.** Probabilities of phonetic matches between five Altaic wordlists (S. Starostin's Japonic *d-reconstruction) obtained by the weighted permutation test

	Mongolic	Tungusic	Korean	Japonic (S. Starostin)
Turkic	7.8 × 10^−5^	8.8 × 10^−4^	0.577	1.8 × 10^−4^
Mongolic	—	<10^−6^	0.085	0.094
Tungusic		—	0.234	0.004
Korean			—	0.011

Statistically significant values under the Holm–Bonferroni correction are shaded: *α* = 0.001, 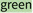; *α* = 0.01, 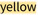; *α* = 0.05, 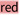.

**Table 3.** Probabilities of phonetic matches between five Altaic wordlists (‘conservative’ Japonic *y-reconstruction) obtained by the weighted permutation test

	Mongolic	Tungusic	Korean	Japonic (‘conservative’)
Turkic	7.8 × 10^−5^	8.8 × 10^−4^	0.577	5.4 × 10^−5^
Mongolic	—	<10^−6^	0.085	0.103
Tungusic		—	0.234	0.006
Korean			—	0.026

Statistically significant values under the Holm-Bonferroni correction are shaded: *α* = 0.001, 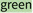; *α* = 0.01, 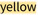; *α* = 0.05, 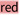. Values between Turkic–Mongolic–Tungusic–Korean are the same as in Table 2.

The two tables show no substantial differences between statistical results of S. Starostin's *d-reconstruction and ‘conservative’ *y-reconstruction of Proto-Japonic. Pairs between the Nuclear Altaic taxa – Turkic, Mongolic and Tungusic – have either highly significant (*α* = 0.001) or very significant (*α* = 0.01) *p-*values.

Japonic demonstrates a weaker result: Japonic–Turkic, highly or very significant *p-*value (*α* = 0.001 or 0.01 depending on the Japonic reconstruction); Japonic–Tungusic, significant *p-*value (*α* = 0.05); and Japonic–Mongolic, insignificant *p-*value. All pairs which involve Korean have insignificant *p-*values.

In total, the algorithm detected 66 *CC*-matched pairs between five involved languages (see the tables in the Supplementary Material). Comparison with the etymological suggestions of Starostin et al. (2003, with further emendations and corrections discussed in the Supplementary Material) shows that, out of these, 11 pairs (17%) are false-positive, i.e. are not supposed to represent true cognates according to the Macro-Altaic paradigm. The number of false-negatives is much greater: 74 pairs thought to be true cognates were not recognized by the *CC*-algorithm (see the Supplementary Material). We count this as an advantage of the method that the number of type 1 errors is relatively small and that the majority of incorrect detections are type 2 errors. Compare this with the Proto-Indo-European–Proto-Uralic comparison where the same algorithm detected precisely those seven pairs which are thought to be true cognates (Kassian et al., 2015: 320) without type 1 and type 2 errors at all. On the other hand, the fact that the majority of presumably true cognate pairs were not detected in the present Altaic comparison (74 pairs) exposes the limitations of the *CC* algorithm in situations of major phonetic sound change (involving, among other things, transitions of consonants from one class to another, mutations in syllabic structure and complex morphophonological processes obscuring the original shape of the root).

## Discussion

Can a genealogical relationship between languages be *proven* by such statistical methods as the permutation test? The strict answer is ‘no’, since demonstrating such a relationship requires going beyond mere substantial number of phonological coincidences in terms of consonant classes between basic vocabularies of the given languages by establishing *regularity*, i.e. the recursive nature of these coincidences which identifies them as regular sound correspondences. Rather, statistically significant *p*-value obtained by such methods should be considered an heuristic indication that the languages in question can be related to each other either genealogically or via intensive contacts.

Conversely, an insignificant *p*-value should not be understood as proving that the languages in question are not related. Although we typically expect the majority of sound shifts to take place within the borders of the same consonant class (the very reason for advocating the idea of consonant classes as such), it goes without saying that certain types of relatively frequent sound shifts also involve consonants from different phonetic classes (e.g. *č/s* or *r/d*) which will not be detected by the algorithm. This effect is particularly crucial in the situation of multiple comparisons when the applied multiple testing correction drastically decreases the significance level (e.g. the Korean-Japonic pair has 0.01 ≤ *p* < 0.05, but this result becomes insignificant in the wider context of the Altaic comparison).

The Nuclear Altaic taxa – Turkic, Mongolic and Tungusic – demonstrate a strong signal of mutual relationship. The nature of this signal lies beyond the scope of the present paper, although, in our opinion, there is ample reason to believe that the majority of matches between the basic vocabularies of Proto-Turkic, Proto-Mongolic and Proto-Tungusic are inherited (although it is also possible to attribute some matches, especially in the Turkic–Mongolic pair, to ancient contacts – at the moment, we are preparing a detailed analysis of Altaic phylogeny where the issue of discrimination between signals of genealogical relationship and ancient loanwords will be addressed). That said, these low *p*-values for the Nuclear Altaic taxa are hardly surprising: bilateral lexical similarities between Turkic, Mongolic and Tungusic can be easily seen with the naked eye and have, for quite a long time, attracted the attention of linguists.

A more important result is significant *p*-values for the Japonic–Turkic and Japonic–Tungusic pairs. If at least some lexical similarities between Proto-Japonic and Proto-Tungusic could be attributed to hypothetical prehistoric contacts, for Proto-Japonic and Proto-Turkic such a scenario is improbable owing to their geographical remoteness. This means that if we are unwilling to assume the scenario of chance coincidence for any pair connected by a significant *p*-value, a genealogical relationship between Japonic and Turkic would be the only sensible conclusion.

Korean shows no positive results with any of its potential Altaic relatives. The *p*-value for Korean–Japonic is significant by itself (0.01 ≤ *p* < 0.05), but it was not sufficient to pass the multiple testing correction. The Korean–Japonic result is somewhat surprising, since a substantial number (if not the majority) of experts in the field accept a relationship between Korean and Japonic as either genealogical or areal (Blažek, 2019: 48–54). However, the overall negative result of Korean is not unexpected, since proponents of the Altaic hypothesis or at least the Korean–Japonic genealogical relationship (e.g. Martin, 1966; Starostin et al., 2003; Robbeets, 2005) are forced to assume various processes of non-initial consonant deletion in Pre-Proto-Korean, on the one hand, and unexplainable initial *s- in some Korean stems (e.g. *spyə́* ‘bone’), on the other. Both factors affect *CC* transcription of the Korean forms and thus impact the results of the permutation test.

Summing up, four taxa – Proto-Turkic, Proto-Mongolic, Proto-Tungusic and Proto-Japonic – demonstrate statistical support for mutual relationship with various degrees of significance. In some cases, geographical distribution allows speculations about prehistoric contacts and loanword exchange, but in the Turkic–Japonic pair contact scenario seems *a priori* less likely than a genealogical one. Korean emerges as either unrelated to any of these four taxa or impervious to the efficacy of the algorithm owing to major mutations undergone by non-initial consonants in Pre-Proto-Korean.

## Supplementary material

For supplementary material accompanying this paper visit https://doi.org/10.1017/ehs.2021.28. click here to view supplementary material

## Research transparency and reproducibility

We provide all linguistic data and technical files used for and produced by the permutation test package which makes our experiment reproducible.
