# Supplementary material for: Permutation test applied to lexical reconstructions partially supports the Altaic linguistic macrofamily
Source: Evol Hum Sci. 2021 Jun 1;3:e32. doi: 10.1017/ehs.2021.28 (PMC10427268; doi:10.1017/ehs.2021.28)
Supplement: Supplementary file 1 [file S2513843X21000281sup.zip › S2513843X21000281sup001.pdf]

Permutation test applied to lexical reconstructions partially supports the Altaic linguistic macrofamily

Alexei S. Kassian,* George Starostin,* Ilya M. Egorov, Ekaterina S. Logunova, Anna V. Dybo

* Corresponding authors (a.kassian@gmail.com, gstarst1@gmail.com)

Text Supplement

Basic information on the language groups 2
CC-matches between the Altaic wordlists 6
Linguistic comments on individual Swadesh forms 14
Supplement references 85

05.05.2021

Basic information on the language groups

Turkic (I. Egorov, A. Kassian, G. Starostin)

The Turkic group consists of several dozens of modern and extinct languages which can be divided into several principal subgroups: Bulghar, Kipchak (a.k.a. Northwestern), Oghuz (a.k.a. Southwestern), Karluk (a.k.a. Southeastern), Yakut (North Siberian), South Siberian. See Johanson 1998: 82–83; Tenishev & Dybo 2002: 5–6; Dybo 2013: 18 and Blažek 2019: 80–90 for overview. Out of these subgroups, Bulghar (represented by modern Chuvash) is the first outlier. The rest of the subgroups are usually named *Common Turkic*; however, since this label is somewhat misleading, we consider it more appropriate to apply the name *Nuclear Turkic* for the non-Bulghar branch of Turkic. Relationships between subgroups within the Nuclear Turkic clade are not entirely clear; there is no consensus on the internal classification of the Turkic languages among experts. Of course, such an uncertainty about the internal classification of the group is a serious obstacle for onomasiological reconstruction.

We accept the Proto-Turkic phonological reconstruction offered in Starostin, Dybo & Mudrak 2003; Dybo 2007. The Proto-Turkic phonemes usually reconstructed as ⟨*e *ẹ *ạ⟩ are actually to be interpreted as *ä *e *ɤ* respectively, but for the sake of convenience and graphical compatibility with older sources we transcribe traditional ⟨*e *ẹ *ạ⟩ as *e *ę̣ *ą̣* respectively.

Mongolic (A. Kassian, G. Starostin)

Mongolic is a relatively recent group without a generally recognized internal classification (see Blažek 2019: 91–105 for overview). We adhere to the lexicostatistical classification offered by Gruntov and Mazo (2015: 212, a study partially based on the authors' own extensive field records) which is very close to the phonological and morphological classification by Rybatzki (2003: 387–389). Mongolic consists of three primary subgroups with minor clades inside:

1. Northern: [Khamnigan, Buriat], [Khalkha, Ordos, Oirat, Kalmyk, New Bargu, Old Bargu, Khoshut].

2. Southern: Middle Mongolic written sources, Mogholi, [Bonan, Dongxiang, East Yugur, Monguor-Minhe, Monguor-Huzhu, Kangjia].

3. Dagur.

Our Proto-Mongolic reconstruction is generally based on Nugteren 2011; Starostin, Dybo & Mudrak 2003: 149–156; Gruntov & Mazo 2015 with minor emendations where necessary.

Tungusic (A. Kassian, G. Starostin)

Internal classification of Tungusic is controversial. There is a nearly universal consensus among linguists that the group consists of four recent clades with an uncertain position of Kilen (a.k.a. Hezhe), see Blažek 2019: 106–117 for overview:

1. Evenic: Even, Evenki, Solon, Negidal, Oroqen.

2. Udiheic: Udihe, Oroch.

3. Nanaic: Nanai, Ulch, Orok.

4. Manchu-Jurchen: Manchu, Xibe, Jurchen(†).

The hierarchical structure of the four clades, however, remains debatable. The “classic” opinion is that the genealogical classification coincides with the geographical distribution that is the “**Amur**” languages (Nanaic, Udiheic) and Manchu-Jurchen form a distinct clade opposed to the ”Northern” languages (Evenic): **[[Amur, Manchu-Jurchen], Evenic]**, see, e.g., Tsintsius 1949; Benzing 1955; Doerfer 1978. This classification is based on a number of phonological and morphological traits and represents a somewhat impressionistic approach.

More recently, Vasilevich (1960) and Sunik (1997: 154) (followed by Robbeets 2015: 18) having applied the same methodology as the previous authors, proposed a different tree structure with Manchu-Jurchen as an outlier: **[[Evenic, Amur], Manchu-Jurchen]**. Bayesian lexicostatistics in Whaley & Oskolskaya 2020 suggests the same subgroupings. The earlier lexicostatistical classification in Vovin 1993 also produced a very similar result.

Finally, Kazama (2003) offers a formal classification based on a closed set of phonological innovations. According to Kazama, Manchu-Jurchen is indeed the first outlier, but Udiheic is included into the Northern subgroups: **[[[Evenic, Udiheic], Nanaic], Manchu-Jurchen]**. Georg (2004) and Janhunen (2012) relying on the historical phonology also suggest the [Evenic, Udiheic] clade, although they follow the traditional view and accept that Manchu-Jurchen is not an outlier, but forms a clade with Nanaic: **[Evenic, Udiheic], [Nanaic, Manchu-Jurchen]**.

In such an uncertain situation, we prefer to adhere to the oldest classification with two primary branches:

1. Southern, i.e., [[Nanaic, Udiheic], Manchu-Jurchen],

2. Northern, i.e., Evenic,

keeping in mind that, firstly, Manchu-Jurchen can actually be a first outlier, and, secondly, Udiheic can actually belong to the Northern branch.

Our Proto-Tungusic reconstruction is generally based on Tsintsius 1949; Starostin, Dybo & Mudrak 2003: 156–163 with minor emendations where necessary. Semantic reconstruction is based on data from main synchronic dictionaries of individual languages and from the wordlists in Kazama 2003. Tsintsius’ (1975; 1977) compendium was used as a secondary source.

Middle Korean (E. Logunova, G. Starostin)

The wordlist is adapted from Logunova 2020. The wordlist is based on texts of the so-called Late Middle Korean period, AD 15th-16th c., since earlier Korean texts are less numerous and do not provide us with necessary lexical data. As stated by Lee and Ramsey (2011: 100–101), the Late Middle Korean corpus reflects “an extremely homogeneous language <...> then spoken by the upper classes of the central region”.

Since our goal is to detect the etymological signal of maximum strength, we regularly replace attested Middle Korean forms with “Proto-Korean” (the term is provisional) reconstructions whenever there is internal Korean evidence that the older root form used to be phonologically different from its attested descendant. This applies, first and foremost, to the synchronic alternations *-r-/-t-* and *-β-/-p-* at the end of verbal stems. We follow Ramsey 1991: 227 and Starostin, Dybo & Mudrak 2003: 163 in reconstructing Proto-Korean **-d-* and **-b-* for these cases, e.g., Middle Korean *tùr- ~ tùt-* ‘to hear’ < Proto-Korean **tùd-*, Middle Korean *nǔ:β- ~ nǔ:p-* ‘to lie’ < Proto-Korean *nǔ:b-*. Martin (Martin 1992: 233–234) tends to reconstruct in such cases simply **-t-* and **-p-* which lenite > *r* and *β* respectively under specific conditions that are still unclear; note that the choice between **d* and **t* or between **b* and **p* is irrelevant for the transcription of consonant classes. An additional case is Proto-Korean **kàč-kàb-* > Middle Korean *kàs-kàβ-* ‘near’ (Lee & Ramsey 2011: 181). It must be stressed that such modifications of Middle Korean forms do not increase the number of *CC*-matches for our Altaic comparison.

Proto-Japonic (G. Starostin)

The Japonic family is commonly believed to consist of two branches: Japanese proper and Ryukyuan, whose splitting clearly predates the Old Japanese epoch (7th-8th centuries AD). Of these branches, the internal history of Ryukyuan is clearly less well understood and studied than that of Japanese proper, due to scarcity of historical sources, insufficient data, and later lexical influence from literary Japanese. Consequently, reconstruction of the Swadesh wordlist for Proto-Japonic is inevitably skewed in favor of Japanese, meaning that our "Proto-Japonic" is essentially equivalent to "Proto-Japanese" (the common ancestor of all Japanese dialects) in terms of phonology and choice of lexemes.

The reconstruction itself largely follows the version in Sergei Starostin (1991), whose differences in comparison with other versions, both older and newer, are largely insignificant from the point of view of *CC*-transcription (the most important differences usually concern reconstruction of vocalism and consonantal codas in triconsonantal structures, mostly irrelevant for the applied algorithm). Arguably the only significant difference between Starostin 1991 and such "conservative" variants as Vovin (2005), and Robbeets (2005; 2015; Robbeets & Bouckaert 2018) is the treatment of word-initial *y-*, reconstructed as **d-* in Starostin's version based on its reflection as such in the Yonaguni dialect of Ryukyuan. Since there is no general consensus on the issue, and since the transcription of the reconstructed phoneme as **d-* or **y-*, with both consonants belonging to different consonantal classes, may at least in theory influence the results of our calculations, we have generated an additional, more “conservative” set of reconstructions which is more close to the ones proposed by Martin (1987) and Vovin (1999) and also “restores” Proto-Japonic **y-*.

The following discrepancies which affect *CC*-transcription can be mentioned:

- ‘to burn’, **dák-* vs. **yák-,*
- ‘good’, **də-* vs. **yò-,*
- ‘mountain’, **dàmà* vs. **yàmà,*
- ‘night’, **duà* vs. **yuyà,*
- ‘to go’, **dúk-* vs. **yúk-,*
- Also **náŋ* vs. **ná* ‘name’ (Vovin 1999: 89).

Proto-Altaic (A. Kassian, G. Starostin, A. Dybo)

In order to better appreciate and understand the differences between the results of automated testing and previously conducted “manual” etymological research, we provide a list of previously generated Proto-Altaic reconstructions for all cases in which at least two of the intermediate reconstructions were judged cognate. The basic reference model for Proto-Altaic referred to in this paper remains Starostin, Dybo & Mudrak 2003, although in some cases the etymological solutions presented in that work have been modified or rejected (notes on particular etymologies are supplied below for each individual word). All etymologies are provided with relevant references to Robbeets 2005; Robbeets 2015; Robbeets & Bouckaert 2018 with further discussion.

When comparing forms of individual subgroups from the angle of classic comparative-historical linguistics, it makes sense to analyze triconsonantal stems *CCC* as *CC-C,* i.e., a bi-consonantal root modified with a certain fossilized suffix, even if there is no internal evidence of such an affixation. Thus S. Starostin et al. and Robbeets are apparently correct in treating Turkic **kuːrt* ‘worm’ as a cognate of Mongolic **kora-* ‘id.’; or Mongolic **kele-* ‘tongue’ as a cognate of Tungusic **xilŋü* ‘id.’. On the other hand, we believe that in absence of internal evidence it is more prudent not to analyze *CC*-stems arbitrarily as *C-C*, e.g., Mongolic **čaga-* ‘white’ and Tungusic **šaː-* ‘id.’ would be treated as unrelated, since there are no inner Mongolic indications that *-ga-* can be singled as a suffix (the correspondence of the initial consonants Mongolic **č-* / Tungusic **š-* is regular).

A special case concerns two etymologies, ‘star’ and ‘tooth’, in which the Mongolian forms are thought to contain the rare suffix **-du* which causes elimination of root-final *-l-* (**...l-du* > **...du*). Thus Mongolic **hodu* ‘star’ < potentially Pre-Mongolic **hol-du*, could be cognate to Turkic **yul-dɨrʸ* ‘id.’ (the correspondence Mongolic **h-* / Turkic **y-* can be regular). Mongolic **sidü* ‘tooth’ < potentially Pre-Mongolic **sil-dü*, could be cognate to Turkic **siːʎ* ‘id.’. The main obstacle for such an analysis is that the Altaic clusters **ld, *ʎd* are retained in Proto-Mongolic (Starostin, Dybo & Mudrak 2003: 84–85) and it seems unrealistic chronologically that a certain cluster is retained within roots, but gets simplified on the secondary morpheme boundaries. Various scenarios are possible (e.g., the Proto-Mongolic clusters **ld* can actually go back to Pre-Proto-Mongolic **lVd* with a vowel syncope).

CC-matches between the Altaic wordlists

Pairs highlighted with red represent chance similarities according to our current view on Altaic historical phonology. Non-highlighted pairs are considered to be true cognates.

Under the tables we offer etymologies which are thought to be true, but were not recognized by the algorithm due to discrepancies in consonant classes.

Table 1. Proto-Turkic / Proto-Mongolic, p = 7.8×10^{-5}

		Proto-Turkic	Proto-Mongolic	*CC*-skeleton
1)	black	**kara*	**kara*	*KR*
2)	dry	**kuːr*	**kahuray*	*KR*
3)	I	**bi*	**bi*	*PH*
4)	kill	**öl*	**ala*	*HL*
5)	long	**urʸɨ*	**urtu*	*HR*
6)	man	**eːr*	**ere*	*HR*
7)	seed	**ur*	**hüre*	*HR*
8)	that	**ti*	**te*	*TH*
9)	we	**bi*	**ba*	*PH*
10)	yellow	**siaːrɨg*	**sira*	*SR*
11)	worm	**kuːrt*	**kora*	*KR*

Cognates not recognized by the algorithm:

- fat: Trk **yaːg* / Mo **ehükü-n*

- heart: Trk *yürek / Mo *ǯirüke-n
- leaf: Trk *yapur-gak / Mo *labči-n ~ *nabči-n
- new: Trk *yaŋɨ ~ *yeŋi / Mo *sine ~ *sini
- stone: Trk *diaːʎ / Mo *čila-hu-n
- who: Trk *kim ~ *kem / Mo *ke-n
- wind: Trk *ye̯l / Mo *salki-n
- ? star: Trk *yul-dɨrʸ / Mo *hodu-n
- ? tooth: Trk. *siːʎ ~ *sɨʎ / Mo *sidü-n

Table 2. Proto-Turkic - Proto-Tungusic, $p = 8.8\times10^{-4}$

		Proto-Turkic	Proto-Tungusic	CC-skeleton
1)	I	*bi	*bi	PH
2)	liver	*biagɨr	*paːki	PK
3)	many	*öːk	*egdi	HK
4)	sleep	*uː	*aːw	HH
5)	that	*ti	*ta	TH
6)	thou	*si	*si	SH
7)	we	*bi	*buː	PH

Cognates not recognized by the algorithm:

- burn tr.: Trk *yak / Tng *deg-ǯe-gi-
- dog: Trk *ɨyt / Tng *ŋinda
- feather: Trk *yüg / Tng *dek-te
- full: Trk *doːl- / Tng *ǯalu-
- give: Trk *be̯ːr / Tng *buː-
- hand: Trk *elg / Tng *ŋaːla
- neck: Trk *boːyɨn / Tng *moŋgo-n
- stone: Trk *diaːʎ / Tng *ǯolo
- tail: Trk *kudruk / Tng *xürgü
- yellow: Trk *siaːrɨg / Tng *soː-

Table 3. Proto-Turkic - Middle Korean, p = 0.577

		Proto-Turkic	Middle Korean	*CC*-skeleton
1)	bark	**ka:p*	*kə̀p*	*KP*
2)	that	**ti*	*tyə́*	*TH*

Cognates not recognized by the algorithm:

- burn tr.: Trk **yak* / Ko *tʰʌ̀-y-ʔó-*
- head: Trk **baʎč* / Ko *mə̀rí*
- leaf: Trk **yapur-gak* / Ko *nípʰ*
- neck: Trk **bo:yɨn* / Ko *mòk*
- rain: Trk **yag-mur* / Mo *pí*
- star: Trk **yul-dɨrʸ* / Mo *pyə̌:r*
- stone: Trk **dia:ʎ* / Mo *tǒ:rh*
- tail: Trk **kudruk* / Mo *skòrí*

Table 4. Proto-Turkic - Proto-Japonic, p = 1.8×10^{-4} or 5.4×10^{-5}

		Proto-Turkic	Proto-Japonic	*CC*-skeleton
1)	bark	**ka:p*	**kapa*	*KP*
2)	black	**kara*	**kùruà*	*KR*
3)	burn tr.	**yak*	**dák- ~ *yák-*	*YK*
4)	I	**bi*	**bà*	*PH*
5)	sleep	**u:*	**úi*	*HH*
6)	this	**kö*	**kə́*	*KH*
7)	we	**bi*	**bà*	*PH*
8)	what	**ne:*	**nà*	*NH*

Cognates not recognized by the algorithm:

- dog: Trk **ɨyt* / Ja **ìnù*
- one: Trk **bir* / Ja **pitə*
- stand: Trk **dur* / Ja **tàt-*
- star: Trk **yul-dɨrʸ* / Ja **pə́sí*
- stone: Trk **dia:ʎ* / Ja **ísì*

Table 5. Proto-Mongolic - Proto-Tungusic, $p < 10^{-6}$

		Proto-Mongolic	Proto-Tungusic	*CC*-skeleton
1)	egg	**emdüge*	**umu:*	*HM*
2)	green	**nogoha*	**ɲog*	*NK*
3)	I	**bi*	**bi*	*PH*
4)	mouth	**ama*	**amŋa*	*HM*
5)	see	**üǯe*	**iče*	*Iʒ*
6)	stone	**čila*	**ǯolo*	*ʒL*
7)	that	**te*	**ta*	*TH*
8)	this	**e*	**e*	*HH*
9)	tongue	**kele*	**xilŋü*	*KL*
10)	we	**ba*	**bu:*	*PH*
11)	snake	**mogay*	**mü:ki*	*MK*
12)	thin	**nim*	**nem*	*NM*
13)	year	**hon*	**aɲŋa*	*HN*

Cognates not recognized by the algorithm:

- ashes: Mo **hüne-sün* / Tng **pulɲe-*
- hair: Mo **hüsü-n* ~ **hü-sün* / Tng **puɲe-*
- red: Mo **hula-han* / Tng **pula-*
- small: Mo **očü-ken* / Tng **ŋüši-*
- tree: Mo **modu-n* / Tng **moː*
- yellow: Mo **sira* / Tng **soː-*
- short: Mo **hokar* ~ **hakor* / Tng **poko-lo*

Table 6. Proto-Mongolic - Middle Korean, p = 0.085

		Proto-Mongolic	Middle Korean	*CC*-skeleton
1)	I	**na*	*nà*	*NH*
2)	leaf	**nabči*	*níp*ʰ	*NP*
3)	that	**te*	*tyə́*	*TH*
4)	this	**e*	*í*	*HH*

Cognates not recognized by the algorithm:

- bird: Mo **siba-hun* / Ko *sǎːy*
- bone: Mo **ya-sun* / Ko *spyə́*
- earth: Mo **sirahu* / Ko *hʌ̀rk*
- horn: Mo *eber / Ko *spɨ́r*
- red: Mo *hula-han / Ko *pɨ̀rk-*
- ? star: Mo **hodu-n* / Ko *pyə̌ːr*

Table 7. Proto-Mongolic - Proto-Japonic, p = 0.094 or 0.103

		Proto-Mongolic	Proto-Japonic	*CC*-skeleton
1)	black	**kara*	**kùruà*	*KR*
2)	I	**bi*	**bà*	*PH*
3)	we	**ba*	**bà*	*PH*

Cognates not recognized by the algorithm:

- blood: Mo **či-sun* / Ja **tí*
- bone: Mo **ya-sun* / Ja **pə̀nià*
- fish: Mo **ǯiga-sun* / Ja **iwua*
- road: Mo **mör* / Ja **mítí*
- stone: Mo **čila-hu-n* / Ja **ísì*
- ? star: Mo **hodu-n* / Ja **pə̀sí*

Table 8. Proto-Tungusic - Middle Korean, p = 0.234

		Proto-Tungusic	Middle Korean	*CC*-skeleton
1)	that	**ta*	*tyə́*	*TH*
2)	this	**e*	*í*	*HH*
3)	who	**ŋü:*	*nú*	*NH*

Cognates not recognized by the algorithm:

- burn tr.: Tng **deg-ǯe-gi-* / Ko *tʰʌ̀-y-ʔó-*
- foot: Tng **palga-n* / Ko *pár*
- hear: Tng **do:ldi:-* / Ko **tɨ̀d-*
- heart: Tng **miawan* / Ko *mʌ̀zʌ̀m*
- neck: Tng **moŋgo-n* / Ko *mòk*
- red: Tng **pula-* / Ko *pɨ̀rk-*
- stone: Tng **ǯolo* / Ko *tǒ:rh*

- sun: Tng **sigu:-n* / Ko *hʌ́y*
- tail: Tng **xürgü* / Ko *skòrí*
- two: Tng **ǯuwer* / Ko *tǔ:*
- go: Tng **ŋene-* / Ko *nyə́-*
- water: Tng **mu:* / Ko *mɨ́r*

Table 9. Proto-Tungusic - Proto-Japonic, p = 0.004 or 0.006

		Proto-Tungusic	Proto-Japonic	*CC*-skeleton
1)	burn tr.	**deg*	**dák- ~ *yák-*	*DK*
2)	I	**bi*	**bà*	*PH*
3)	knee	**peɲ*	**pínsá*	*PN*
4)	sleep	**a:w*	**úi*	*HH*
5)	water	**mu:*	**mí*	*MH*
6)	we	**bu:*	**bà*	*PH*
7)	near	**daga*	**tìkà*	*TK*

Cognates not recognized by the algorithm:

- dog: Tng **ŋinda* / Ja **ìnù*
- know: Tng **sa:-* / Ja **sír-*
- stone: Tng **ǯolo* / Ja **ísì*

Table 10. Middle Korean - Proto-Japonic, p = 0.011 or 0.026

		Middle Korean	Proto-Japonic	*CC*-skeleton
1)	bark	*kə̀p*	**kapa*	*KP*
2)	good	*tyǒːh*	**də̀*	*TH*
3)	many	*mǎːn*	**mana*	*MN*
4)	not	*àní*	**an*	*HN*
5)	swim	*hə̀y*	**ə̀yə̀*	*HH*
6)	that	*kɨ̀*	**ká*	*KH*
7)	thou	*nə̀*	**ná*	*NH*
8)	snake	*pʌ́yám*	**pàimV*	*PM*

Cognates not recognized by the algorithm:

- belly: Ko *pʌ́y* / Ja **pàrà*
- bone: Ko *spyə́* / Ja **pə̀nià*
- burn tr.: Ko *tʰʌ̀-y-ʔó-* / Ja **dák-*
- fingernail: Ko *tʰóp* / Ja **túmái*
- cloud: Ko *kúrùm* / Ja **kùmua*
- fire: Ko *pɨ́r* / Ja **pə̀-i*
- I: Ko *nà* / Ja **a*
- moon: Ko *tʌ́r* / Ja **tùkù-i*
- star: Ko *pyə̌ːr* / Ja **pə́sí*
- stone: Ko *tǒːrh* / Ja **ísì*
- tongue: Ko **hyət* / Ja **sìtà*
- water: Ko *mɨ́r* / Ja **mí-n*
- white: *hʌ́y-* / Ja **sìruà-*

Linguistic comments on individual Swadesh forms

Note on transcription. All linguistic data in the present document are encoded in the unified transcription system of the Global Lexicostatistical Database project, which is generally based on the IPA alphabet, with a few specific discrepancies, e.g., *c* stands for IPA *ʦ*, *š* for IPA *ʃ* (http://starling.rinet.ru/new100/UTS.htm). Traditional or orthographic representa-tions are enclosed in ⟨angle brackets⟩.

1. 'all'

Proto-Turkic. **baːr-ɨ* (Sevortyan et al. 1974–: vol. 2: 61; Dybo 2013: 27), attested in all subgroups. Meaning 'all (*omnis*)'. Derived from nominal predicative **baːr* 'existence, there is, there exists' with the help of the izafet suffix. Distinct from **bütü-n* 'all (*totus*)' (Sevortyan et al. 1974–: vol. 2: 152; Dybo 2013: 21), attested in Chuvash and the majority of Nuclear Turkic languages; a deverbative from **bütü-* to finish (intr.)'.

Proto-Mongolic. **bügü-de* (Gruntov & Mazo 2015: 216), attested as a basic term for 'all (*omnis*)' in the Northern subgroup and Middle Mongolic (Orlovskaya 1999: 66). This stem has also survived as Dagur *bugede* 'all' (Martin 1961: 127), but its exact meaning and status are unknown. Final *-de* can be a fossilized locative exponent. In some modern Northern lects, 'all (omnis)' is expressed by the stem **bükü* (Gruntov & Mazo 2015: 216) which can either be related to **bügü-de* with an irregular *k* ~ *g* fluctuation or represent a separate formation (e.g., Middle Mongolian **bükü* means 'every', Orlovskaya 1999: 66). Both **bügü-de* and **bükü* might be eventually derived from **büyi-* 'to be'. The second candidate is **kow* (Nugteren 2011: 406; Gruntov & Mazo 2015: 217) which is a basic term for 'all (*omnis*)' in Dagur (acc. to the examples in Martin 1961) and, extended with a *l*-suffix, in some Southern lects, namely Huzhu and Kangjia. In Khalkha, Kalmyk and Buriat, **kow* functions as a particle 'completely, entirely'. We take **bügü-de* and **kow* as synonyms. Inherited terms are frequently superseded with loans (Gruntov & Mazo 2015: 217).

Proto-Tungusic. An unstable item prone to interdialect borrowing, as truly noted in Kazama 2003: 57–58. The most reliable candidate is **gemu* (Tsintsius 1975: 179; Starostin, Dybo & Mudrak 2003: 539) which is attested in Manchu-Jurchen (Manchu *gemu*) and Nanaic (Nanai *xem*, Orok *gem*, Ulch *xem*), where it means 'all (omnis)'. At least in Manchu and Nanai, this one is a basic term for 'all (omnis)'. The irregular sound correspondences (*g-* ~ *x-*), however, suggest that these forms are unrelated to each other or some of them are non-inherited. We prefer to leave the slot empty.

Middle Korean. *tǎː* (Starostin, Dybo & Mudrak 2003: 1394), *mòt-á* ~ *mòt-ʌ́n* (Starostin, Dybo & Mudrak 2003: 957).

Proto-Japonic. **múina* (Starostin, Dybo & Mudrak 2003: 939), **múCí-nà* (Vovin 1999: 87). Attested in Japanese and Ryukyuan. Final **-na* is possibly the same suffixal component as in *suku-na* 'few', etc.

2. 'ashes'

Proto-Turkic. **kül* (Sevortyan et al. 1974–: vol. 5: 137; Dybo 2013: 75), attested in all subgroups.

Proto-Mongolic. **hüne-sün* (Nugteren 2011: 369; Gruntov & Mazo 2015: 217), attested as a basic term in all subgroups.

Proto-Tungusic. **pulɲe-* (Tsintsius 1977: 347; Starostin, Dybo & Mudrak 2003: 1170), attested as a basic term in all four subgroups, where it functions as a stem modified with various desemanticized suffixes.

Middle Korean. *čʌ́y* (Starostin, Dybo & Mudrak 2003: 1539).

Proto-Japonic. **páp(u)í* (Starostin, Dybo & Mudrak 2003: 1085), **pápÍ* (Vovin 1999: 87). Attested in Japanese and Ryukyuan.

Etymological notes. Mongolic **hüne-sün*, Tungusic **pulɲe-* possibly < Proto-Altaic **pʰoʎ́ɲe* (Starostin, Dybo & Mudrak 2003: 1170). The onset correspondence is regular, but the correspondence Mongolic *-*n*- / Tungusic *-*lɲ*- is less evident since the Altaic medial *lC*- and *ʎ́C*-clusters are generally retained in Mongolic (Starostin, Dybo & Mudrak 2003: 84) except for the assumed cluster *-*ʎ́ɲ*- supported only by this particular example.

3. 'bark'

Proto-Turkic. Formally **kaːp-uk* (Sevortyan et al. 1974–: vol. 5: 168; Tenishev 2001: 107; Dybo 2013: 90) is the best candidate, since this stem is attested as a basic designation of 'bark' in Chuvash and a number of Nuclear Turkic lects (Oghuz, Karluk, Kipchak), although in non-Chuvash it usually shows polysemy 'bark / crust / peel (of fruit)'. Apparently **kaːp-uk* is a regular deverbative from **kaːp-* 'to cover'. The second candidate is **kaðɨrʸ* (Sevortyan et al. 1974–: vol. 5: 211; Tenishev 2001: 106; Dybo 2013: 96) which serves as the basic term for 'bark' in Yakut-Dolgan and in some other Nuclear Turkic lects. Besides that, **kaðɨrʸ* is frequently attested as a *terminus technicus* 'bark taken off the tree, bark as material', thus in Chuvash, Bashkir dialects etc. where it is opposed to the reflexes of **kaːp-uk* with the basic meaning 'bark'. Such synchronic oppositions suggest the Proto-Turkic meaning 'bark taken off the tree, bark as material' for **kaðɨrʸ*. It is also likely that in Pre-Proto-Turkic, **kaðɨrʸ* meant 'bark (in general)', whereas **kaːp-uk* is a new formation introduced not long before the break-up of Proto-Turkic. We fill the slot with **kaːp-uk*.

Proto-Mongolic. **kolto-sun* (Gruntov & Mazo 2015: 218), attested as a basic term in Northern and Dagur. Superseded with **ara-sun* 'skin' (q.v.) in Southern.

Proto-Tungusic. **xura-kta* (Tsintsius 1977: 282; Starostin, Dybo & Mudrak 2003: 827), attested as a basic term in Evenic (e.g., Even), Udiheic (e.g., Udihe) and Nanaic (Nanai, Ulch); it means 'inner bark (of tree)' in Manchu. A deverbative from **xura-* 'to cover with bark' (Tsintsius 1977: 282).

Middle Korean. *kə̀p-čìr* (Starostin, Dybo & Mudrak 2003: 764).

Proto-Japonic. **kapa* (Starostin, Dybo & Mudrak 2003: 764, with uncertainty about tonal reconstruction), **kàpà* (Vovin 1999). With polysemy ʽskin / bark' reconstructible already for Proto-Japonic (see also ʽskin').
Attested in Japanese and Ryukyuan.

Etymological notes. Korean *kə̀p-čìr*, Japonic **kapa* < Proto-Altaic **kʰá:pʰà* (Starostin, Dybo & Mudrak 2003: 764; Robbeets 2005: 605–606; Robbeets & Bouckaert 2018). Turkic **ka:p-uk* is phonetically compatible with Korean *kə̀pʰ-* and Japonic **kapa*, but since **ka:p-uk* is an inner Turkic deverbative from 'to cover' we are likely to deal with a chance coincidence.

4. 'belly'

Proto-Turkic. **kạrɨm* ~ **kạrɨn* (Sevortyan et al. 1974–: vol. 5: 321; Tenishev 2001: 277; Dybo 2013: 101), attested as a basic term in Chuvash and in almost all Nuclear Turkic subgroups.

Proto-Mongolic. **kebeli* (Nugteren 2011: 408; Gruntov & Mazo 2015: 218), attested as a basic term in Southern and Dagur. In the Northern lects, this stem shifted to such meanings as 'paunch' or 'embryo', having been superseded with **gede-sün* whose original meaning was apparently 'stomach, intestines' (Nugteren 2011: 338; Gruntov & Mazo 2015: 218).

Proto-Tungusic. An unstable concept. The best candidate seems to be **uri* (Tsintsius 1977: 281; Starostin, Dybo & Mudrak 2003: 1064), attested as a basic term in Evenic (at least in Even and some Evenki dialects). There is also a suffixed stem **uri-ptun* 'apron', attested in Evenic and Nanaic (Ulch) which should prove the antiquity of the meaning 'belly' for **uri*. Distinct from **xemu-gde* 'intestines' (Tsintsius 1977: 451; Starostin, Dybo & Mudrak 2003: 775) which frequently shifted towards the general meaning 'belly'. In Manchu-Jurchen, an inherited term was superseded with a Mongolian loan.

Middle Korean. *pʌ́y* (Starostin, Dybo & Mudrak 2003: 1131).

Proto-Japonic. **pàrà* (Starostin, Dybo & Mudrak 2003: 1131; Vovin 1999: 87). Attested in Japanese proper. In Ryukyuan, the meaning 'belly' is expressed by reflexes of Proto-Japonic **bàtà* which means 'intestines' in Japanese proper (Starostin, Dybo & Mudrak 2003: 365); we assume a semantic innovation in Ryukyuan.

Etymological notes. Korean *pʌ́y*, Japonic **pàrà* < Proto-Altaic **pʰè:yló* (Starostin, Dybo & Mudrak 2003: 1131; Robbeets 2005: 400; Robbeets & Bouckaert 2018).

5. 'big'

Proto-Turkic. An unstable item. In Dybo 2013: 136, two main candidates for the status of Proto-Turkic 'big' are discussed. (1) **bạng*, meaning 'big' in Chuvash and 'elder, adult (esp. of cattle)' in Nuclear Turkic (Sevortyan et al. 1974–: vol. 7: 34; Dybo 2013: 127). (2) **ulug*, meaning 'big' in Tofa-Tuvinian, South Siberian, Karluk, Oghuz, Kipchak, Yakut-Dolgan, but also frequently attested with the specific meaning 'great' (Sevortyan et al. 1974–: vol. 1: 593; Tenishev 2001: 684; Dybo 2013: 120). In the Bulghar subgroup, **ulug* is attested in the medieval collocation "Great Tarkhan". Thus, the semantic reconstruction of Proto-Turkic **ulug* would be either 'big' or 'great'. Since the shift 'big' > 'great' seems to be more frequent cross-linguistically than *vice versa*, the meaning 'big' for **ulug* could be preferable and therefore this implies

that we should rule out the stem *bạng. Nevertheless we agree to fill the Proto-Turkic slot with two technical synonyms: *ulug and *bạng (note that the choice is irrelevant to further phylogenetic purposes). Both stem can be analyzed as *bạn-g and *ulu-g with the common adjective g-suffix.

Proto-Mongolic. *hike (Nugteren 2011: 545; Gruntov & Mazo 2015: 219), attested as a basic term in all three subgroups.

Proto-Tungusic. An unstable item (Kazama 2003: 113). The best candidate is *amba (Tsintsius 1975: 37; Kazama 2003: 113; Starostin, Dybo & Mudrak 2003: 295), having the generic meaning 'big' in Manchu-Jurchen and such related meanings in the Nanaic and Udiheic subgroups as Nanai 'great; enough; nobleman', Ulch 'very big; very', Oroch 'very'. A more widely spread word amba 'evil spirit' can be a tabooistic derivation < 'noble (man)' or even unrelated. The second candidate could be *sag-da- (Tsintsius 1977: 53; Kazama 2003: 113; Starostin, Dybo & Mudrak 2003: 1196), a basic term for 'big' in Udiheic and Kilen, but its meaning 'old; senior' in other subgroups as well as the synchronic polysemy 'big / old, senior' in Oroch point out that 'big' is an Udiheic innovation. No reliable candidates for the Proto-Evenic level can be proposed; cf. *pegdi (Tsintsius 1977: 359; Kazama 2003: 113; Starostin, Dybo & Mudrak 2003: 1083), a basic term for either 'big' or 'many' in Evenki depending on the dialect. The forms of the shape *daːi* 'big' (Tsintsius 1975: 190) in Nanaic might be a Chinese loan. Other isolated candidates are even weaker.

Middle Korean. kʰɨ́- (Starostin, Dybo & Mudrak 2003: 832).

Proto-Japonic. *ə̀pə̀- (Starostin, Dybo & Mudrak 2003: 514), *òpò (Vovin 1999: 87). Attested in Japanese and Ryukyuan.

6. 'bird'

Proto-Turkic. *kuĺ (Sevortyan et al. 1974–: vol. 6: 180; Tenishev 2001: 168; Dybo 2013: 137), attested as a basic term in all subgroups except for Chuvash (where it is still retained in the fixed collocation 'beasts and birds') and Yakut (where it shifted towards the meaning 'duck'). Note the stem *sɨb-čuk (Dybo 2013: 139, differently in previous sources) which can be safely reconstructed with the meaning 'small bird' for Proto-Turkic. Since *-čuk is a diminutive suffix, *sɨb- might actually be a Pre-Proto-Turkic root for 'bird'; such a scenario would fit external etymological connection of Turkic *sɨb-.

Proto-Mongolic. *siba-hun (Nugteren 2011: 488; Gruntov & Mazo 2015: 219), attested as a basic term in Northern and Southern. In Dagur, it shifted towards the meaning 'falcon' having been superseded with a Tungusic loan.

Proto-Tungusic. *gasa (Tsintsius 1975: 143; Kazama 2003: 29; Starostin, Dybo & Mudrak 2003: 532), attested as a basic term in Manchu (opposed to onomatopoeic cecike 'small bird') and Nanaic and Udiheic lects: Nanai 'bird; duck', Udihe 'bird; duck', Kilen 'bird'. In Ulch and Orok, it has the more specific meaning 'water-bird (duck etc.)'. In the Evenic subgroup, *gasa can mean 'crane' or 'swan'. It is most natural that such a wide range of meanings - duck, crane, swan - originated from generic semantics, i.e. 'bird', which is an additional additional argument in favor of *gasa. The primary root *gasa was superseded with *deg-i 'bird' (Tsintsius 1975: 228–229; Starostin, Dybo & Mudrak 2003: 1359) in Evenic - a transparent new formation from *deg- 'to fly' q.v.

Middle Korean. *sǎ:y* (Starostin, Dybo & Mudrak 2003: 1288).

Proto-Japonic. **tərí* (Starostin, Dybo & Mudrak 2003: 1463), **tórí* (Vovin 1999: 87). Attested in Japanese and Ryukyuan.

Etymological notes. We follow Dybo (2013: 139) in treating Mongolic **siba-hun* 'bird', Korean *sǎ:y* 'bird' and potentially Pre-Proto-Turkic **sɨb-* 'bird' as related < Altaic **siba-* with an occasional weakened reflex of **-b-* in Korean: **siba-i* > *sǎ:y* (see Starostin, Dybo & Mudrak 2003: 36 where such cases are discussed). A different etymological analysis is offered in Starostin, Dybo & Mudrak 2003: 1257, 1288, where the Turkic data are not fully accounted for. Robbeets (2005: 777), following Martin 1966: 226; Martin 1996: 38; Whitman 1985: 233, compares Korean *sǎ:y* with Japonic *sagi* 'heron'. However, this etymology is not mentioned in her later paper (Robbeets & Bouckaert 2018).

7. 'to bite'

Proto-Turkic. **ɨsɨr* (Sevortyan et al. 1974–: vol. 1: 671; Dybo 2013: 157), meaning 'to bite' in all Nuclear Turkic subgroups except for Altay, not attested in Bulghar. In Chuvash, 'to bite' is expressed by **yir-t* which means 'to tear (to pieces)' in Nuclear Turkic (Dybo 2013: 162). The stem represents an intensive in *-t-* from Proto-Turkic **yɨr* 'to tear; to split lengthwise' (Sevortyan et al. 1974–: vol. 4: 203), thus the Chuvash meaning 'to bite' should be secondary. Distinct from Proto-Turkic **da:la-* 'to tear with teeth'.

Proto-Mongolic. There are two Common Mongolic verbs which can be translated as 'to bite': **kaǯa* (Nugteren 2011: 401; Gruntov & Mazo 2015: 220) and **ǯahu* (Nugteren 2011: 383; Gruntov & Mazo 2015: 220). As stated by Gruntov and Mazo and confirmed by the main lexicographic sources, **kaǯa* rather means 'to bite, pierce with teeth', whereas **ǯahu* usually means 'to grab with teeth, hold with teeth'. The same semantic opposition is likely to be reconstructed for Proto-Mongolic. According to our semantic specifications we fill the slot with **kaǯa*.

Proto-Tungusic. An unstable concept, especially in the Nanaic and Udiheic subgroups. An appropriate candidate is **kik-* (Tsintsius 1975: 391–392; Starostin, Dybo & Mudrak 2003: 677) attested as a basic term in Evenki, Solon, Negidal and as a second synonym for 'to bite' in Even, thus it can be safely reconstructed at least for the Proto-Evenic level. The second candidate is **sia-* (Tsintsius 1977: 69; Starostin, Dybo & Mudrak 2003: 1246) meaning 'to bite' in Manchu and 'to chew' in other subgroups. We treat **kik-* and **sia-* as technical synonyms.

Middle Korean. *mɨ̀r-* (Starostin, Dybo & Mudrak 2003: 943).

Proto-Japonic. **kàm-* (Starostin, Dybo & Mudrak 2003: 662, Vovin 1999: 87). Attested in Japanese and Ryukyuan.

8. 'black'

Proto-Turkic. **kara* (Sevortyan et al. 1974–: vol. 5: 286; Tenishev 2001: 592; Dybo 2013: 167), retained everywhere.

Proto-Mongolic. **kara* (Nugteren 2011: 404; Gruntov & Mazo 2015: 220), attested as a basic term in all three subgroups. The stem is widely spread and deeply rooted in Mongolic. Although borrowing from Turkic cannot be excluded, there are no specific arguments from phonology, morphology, or internal distribution to unequivocally prefer areal borrowing from Turkic **kara* 'black' to a scenario of common linguistic ancestry.

Proto-Tungusic. **saka-riːn* ~ **saka-liːn* (Tsintsius 1977: 56; Kazama 2003: 127), attested as a basic term in Jurchen, Nanaic and Udiheic. The second candidate is **koŋna-* 'black' (Tsintsius 1975: 413) in Evenic, without further Tungusic etymology. If Manchu-Jurchen is indeed the first outlier, **saka-riːn* ~ **saka-liːn* has the advantage, but it is more prudent to treat **saka-riːn* ~ **saka-liːn* and **koŋna-* as technical synonyms.

Middle Korean. *kə̆ːm-* (Starostin, Dybo & Mudrak 2003: 852).

Proto-Japonic. **kùruà-* (Starostin, Dybo & Mudrak 2003: 651), **kùrwò* < **kura-Cu* (Vovin 1999: 87). Attested in Japanese and Ryukyuan.

Etymological notes. Turkic **kara,* Mongolic **kara*, Japonic **kùruà-* < Proto-Altaic **kàru* (Starostin, Dybo & Mudrak 2003: 651; Robbeets 2005: 660; Robbeets & Bouckaert 2018), correspondences seem regular.

9. 'blood'

Proto-Turkic. **kiaːn* (Sevortyan et al. 1974–: vol. 5: 251; Dybo 2013: 170), retained everywhere.

Proto-Mongolic. **či-sun* (Nugteren 2011: 304; Gruntov & Mazo 2015: 220), attested as a basic term in all three subgroups.

Proto-Tungusic. **seːg-kse* (Tsintsius 1977: 138–139; Kazama 2003: 22; Starostin, Dybo & Mudrak 2003: 1224), attested as a basic term in all four subgroups. A regular deverbative from relict **seːgV-* 'to bleed, flow (of blood)' retained in some Evenic lects.

Middle Korean. *pʰí* (Starostin, Dybo & Mudrak 2003: 359).

Proto-Japonic. **tí* (Starostin, Dybo & Mudrak 2003: 401, Vovin 1999: 87). Attested in Japanese and Ryukyuan.

Etymological notes. Mongolic **či-sun*, Japonic **tí* < Proto-Altaic **či̯úː-(nu)* (Starostin, Dybo & Mudrak 2003: 50, 401; Robbeets 2005: 402, 861; Robbeets & Bouckaert 2018), correspondences seem regular.

10. 'bone'

Proto-Turkic. **siŋök* (Sevortyan et al. 1974–: vol. 7: 357; Tenishev 2001: 260; Dybo 2013: 172), retained in all subgroups. It is likely that the Proto-Turkic meaning of **siŋök* should be reconstructed as 'bone (in general), tubular bone' as opposed to a more specific term **kemük* 'spongy bone' (Sevortyan et al. 1974–: vol. 5: 36; Tenishev 2001: 261; Dybo 2013: 174).

Proto-Mongolic. **ya-sun* (Nugteren 2011: 544; Gruntov & Mazo 2015: 221), attested as a basic term in all three subgroups.

Proto-Tungusic. The plain stem **giram* is only retained in Manchu where it means 'corpse' (Norman 1978: 108), whereas the meaning 'bone' is expressed with two derived stems: **giram-ksa* in Nanaic, Udiheic and Manchu-Jurchen and **giram-na* in Evenic (Tsintsius 1975: 154; Kazama 2003: 23; Starostin, Dybo & Mudrak 2003: 546). The original meaning 'bone' for **giram* (later shifted to 'corpse' in Manchu) follows from such derivatives as, e.g., Manchu *gira-tu* 'big-boned (of livestock)' or Evenki *giram-u-* 'to ache (of bones)'.

Middle Korean. *spyə́*, possibly < Proto-Korean prefixed **s=pyə* (Starostin, Dybo & Mudrak 2003: 1132).

Proto-Japonic. **pə̀nià* (Starostin, Dybo & Mudrak 2003: 1130), **pone* (Vovin 1999: 87). Attested in Japanese and Ryukyuan.

Etymological notes. Provisionally we follow Starostin, Dybo & Mudrak 2003: 1130 in treating Mongolic **ya-sun*, Korean *spyə́* and Japonic **pə̀nià* as related < Altaic *p^hèyńé*, although the whole etymology faces difficulties, e.g., initial *s-* in Korean (a fossilized prefix? see notes on *spɨr* 'horn') or almost total reduction of the consonant skeleton in Mongolic. Robbeets (Robbeets 2005: 400, 530–531; Robbeets & Bouckaert 2018) connects the Korean form with the Japonic one, leaving the Mongolic form beyond the comparison.

11. 'breast (chest)'

Proto-Turkic. **gökür^y* or its diminutive **gökr^y-ek* > **gökr-ek* (Sevortyan et al. 1974–: vol. 3: 54, 5: 136; Tenishev 2001: 272; Dybo 2013: 178) are retained as a basic term for 'chest, breast (without sex differentiation)' in the majority of subgroups incl. Chuvash and Ancient Turkic (Clauson 1972: 712, 714).

Proto-Mongolic. **čeheǯi* (Nugteren 2011: 300; Gruntov & Mazo 2015: 221), attested as a basic term in Northern and Southern, meaning 'chest (in general, applicable to both men and women)'. Distinct from phonetically similar **ebčehü-n* (Nugteren 2011: 321; Gruntov & Mazo 2015: 221), whose original meaning was rather 'sternum, breast bone' as proposed by Gruntov and Mazo; morphological analysis of **ebčehü-n* is not clear and it is possible that historically **čeheǯi* and **ebčehü-n* are cognate stems. Distinct from **köke-n* 'female breast' (Nugteren 2011: 425; Gruntov & Mazo 2015: 221).

Proto-Tungusic. **tüŋe-n* (Tsintsius 1977: 184), retained as a basic term in all four subgroups. Distinct from **xuku-n* 'female breast', formally derived from **xuku-* 'to suck (breast)' (Tsintsius 1977: 254–255; Starostin, Dybo & Mudrak 2003: 713). For instance, in some Evenki dialects, **xuku-n* has acquired the generic meaning 'human breast, chest', whereas **tüŋe-n* shifted to the meaning 'chest of animal'.

Middle Korean. *kàsʌ́m* (Starostin, Dybo & Mudrak 2003: 769).

Proto-Japonic. **múnà-i* (Starostin, Dybo & Mudrak 2003: 928). Generic term for both male and female breast / chest; attested in Japanese and Ryukyuan. There is also a specific separate term for female breast, clearly of expressive origin: **tì, *tìtí* (Starostin, Dybo & Mudrak 2003: 409), **ti, *titi* (Vovin 1999: 87).

12. ‘to burn (tr.)’

Proto-Turkic. **yak* (Sevortyan et al. 1974–: vol. 4: 81; Tenishev 2001: 362; Dybo 2013: 187), attested as ‘to burn (tr.)’ in Nuclear Turkic: Oghuz, Kipchak as well as in some Ancient Turkic sources (Clauson 1972: 897). The antiquity of this verb is proven by the deverbative stem **yak-tu* ‘light’, attested in Chuvash and Nuclear Turkic (Sevortyan et al. 1974–: vol. 4: 62). In light of **ya(-)n* ‘to burn (intr., of things)’ and **ya(-)l-ɨn* ‘flame’, Räsänen (1957: 155) analyzes **yak* as **ya-k* and postulates a unique causative suffix **-k*, not attested elsewhere. This is not excluded, but needs additional evidence and seems unnecessary in view of external Altaic etymology. In many Turkic lects, the meaning ‘to burn (tr.)’ is expressed by causatives from various verbs ‘to burn (intr.)’. The most widespread causatives stems are **yan-tur* ‘to burn (tr.)’ (Dybo 2013: 188) and **köŋ-tur* ‘to burn (tr.)’ (Dybo 2013: 189), derived from **yan* ‘to burn, burn down (intr., of things)’ and **köŋ* ‘to burn (intr., of fire, firewood)’ respectively. Formally **yan-tur* is a candidate for a Proto-Turkic term ‘to burn (tr.)’, since this causative is attested in Chuvash as well as in Nuclear Turkic (Oghuz, Kipchak, Karluk), but equally well **yan-tur* might represent parallel innovations in individual subgroups or lects.

Proto-Mongolic. **sita-ha* (Nugteren 2011: 493; Gruntov & Mazo 2015: 221), attested as a basic term in all three subgroups. A regular causative from Common Mongolic **sita* ‘to burn (intr.)’.

Proto-Tungusic. **deg-ǯe-gi-* (Tsintsius 1975: 281–282; Starostin, Dybo & Mudrak 2003: 469), attested as a basic term in all four subgroups. A causative from **deg-ǯe-* ‘to burn (intr.)’.

Middle Korean. *tʰʌ̀-y-ʔó-* (Starostin, Dybo & Mudrak 2003: 469).

Proto-Japonic. **dák-* (or **yák-*) (Starostin, Dybo & Mudrak 2003: 469; Vovin 1999: 87).

Etymological notes. Turkic **yak*, Tungusic **deg-ǯe-gi-*, Korean *tʰʌ̀-y-ʔó-*, Japonic **dák-* < Proto-Altaic **dékà* (Starostin, Dybo & Mudrak 2003: 469), correspondences seem regular. Robbeets (Robbeets 2005: 391, 847; Robbeets 2015: 139–140; Robbeets & Bouckaert 2018) accepts this etymology, but does not include the Tungusic form into the comparison.

13. ‘fingernail’

Proto-Turkic. **dɨrŋa-k* (Sevortyan et al. 1974–: vol. 3: 348; Tenishev 2001: 258; Dybo 2013: 193), retained in all subgroups. Sometimes contaminated with the unrelated verb **dɨrma* ‘to scratch’ and its derivative **dɨrma-k* which also has the meaning ‘fingernail, claw’.

Proto-Mongolic. **kimu-sun* (Nugteren 2011: 413; Gruntov & Mazo 2015: 222), attested as a basic term in all three subgroups.

Proto-Tungusic. **xosi:-kta* (Tsintsius 1977: 26–27; Starostin, Dybo & Mudrak 2003: 813), attested as a basic term with polysemy ‘fingernail / claw’ in Evenic, Udiheic and Nanaic and only as ‘claw’ in Manchu; a deverbative from **xosi:-* ‘to scrape, scratch’. Manchu *xitaxu:n* ‘fingernail’ might originate from **kiata-kun* and correspond to a Nanai word for ‘bar, ingot’ (Starostin, Dybo & Mudrak 2003: 632).

Middle Korean. *tʰóp* (Starostin, Dybo & Mudrak 2003: 1445).

Proto-Japonic. **túmái* (Starostin, Dybo & Mudrak 2003: 1445), **túmá-Ci* (Vovin 1999: 89). Attested in Japanese and Ryukyuan.

Etymological notes. Starostin, Dybo & Mudrak 2003: 1445 treat Korean *tʰóp* and Japonic **túmá-i* as cognates < Proto-Altaic **tʰi̯úpʰo*, assuming a certain *n*-suffix in Pre-Proto-Japonic and the (occasional?) assimilation **túmá-* < **túpá-n-*. Although there is no direct evidence for such a nasal suffix in the Japonic stem, Martin 1966: 200 offers several additional instances for the correspondence Korean *p* / Japonic **m*, which makes the whole comparison acceptable. Robbeets (Robbeets 2005: 113; Robbeets & Bouckaert 2018) does not accept this etymology, treating Japonic **túmá-i* as a deverbative from Japonic **tuma-* 'to pick (something) up, pluck'; however, such a semantic shift seems typologically unusual (the nominal meaning 'claw, nail' is frequently connected to the verbal meaning 'to scratch', cf. the Tungusic example below, but we are not aware of any examples of the development 'pluck smth.' > 'fingernail').

14. 'cloud'

Proto-Turkic. **bulɨt* (Sevortyan et al. 1974–: vol. 2: 262; Tenishev 2001: 24; Dybo 2013: 197), retained in all subgroups. External comparison suggests the morphological analysis **bulɨ-t*.

Proto-Mongolic. **ehüle-n* (Nugteren 2011: 334; Gruntov & Mazo 2015: 222), attested as a basic term in all three subgroups.

Proto-Tungusic. **tuge-kse* (Tsintsius 1977: 208–209; Starostin, Dybo & Mudrak 2003: 1467), attested as a basic term in all four subgroups. Derived from **tuge* 'winter' (Tsintsius 1977: 204–205).

Middle Korean. *kúrùm* (Starostin, Dybo & Mudrak 2003: 835).

Proto-Japonic. **kùmua* (Starostin, Dybo & Mudrak 2003: 835), **kùmù[C]a* (Vovin 1999: 87). Attested in Japanese and Ryukyuan.

Etymological notes. Korean *kúrùm*, Japonic **kùmua* < Proto-Altaic **kʰòlmV* (Starostin, Dybo & Mudrak 2003: 835), correspondences seem regular. Robbeets does not mention this comparison in Robbeets & Bouckaert 2018. However, she accepts it in her earlier work (Robbeets 2005: 309, 337).

15. 'cold'

Proto-Turkic. **sogɨ-k* (Sevortyan et al. 1974–: vol. 7: 272; Tenishev 2001: 15; Dybo 2013: 200), meaning 'cold (of things)' in all Nuclear Turkic subgroups. Because of minor vowel irregularities in some Karluk and Kipchak lects, it is proposed in Tenishev & Dybo 2006: 110; Dybo 2013: 201 to reconstruct a second synonymous Proto-Turkic stem **sa̧gu-k* 'cold (of things)' which is intertwined with **sogɨ-k* in some Nuclear Turkic subgroups. However, such a solution seems somewhat artificial. We prefer to reconstruct a single stem **sogɨ-k* 'cold (of things)', a standard deverbative from Proto-Turkic **sogɨ* 'to become cold'. Chuvash *sivə* 'cold' apparently belongs here, although Chuvash *-i-* is irregular regardless of whether the protoform is reconstructed as **sogɨ-k* or **sa̧gu-k*. Distinct from **dum-lɨk* 'cold (of weather)' attested in Ancient Turkic and Yakut (Tenishev 2001: 14; Dybo 2013: 203).

Proto-Mongolic. **köyi-ten* (Nugteren 2011: 424; Gruntov & Mazo 2015: 222), attested as a basic term in all three subgroups.

Proto-Tungusic. The following lexical oppositions can be reconstructed for individual subgroups. (1) Evenic **gil-či* ~ **gil-si* 'cold (of objects)' (Tsintsius 1975: 151; Starostin, Dybo & Mudrak 2003: 545) vs. **xiŋüː-n-* 'cold (of weather)' (Kazama 2003: 120; Tsintsius 1975: 321; Starostin, Dybo & Mudrak 2003: 803), a deverbative from Proto-Tungusic **xiŋüː-* 'to freeze (intr.)'. (2) Nanaic: **gil-či* ~ **gil-si* 'cold (of objects)' vs. **ɲuŋde-* ~ **ɲeŋde-* 'cold (of weather)' (Kazama 2003: 120; Tsintsius 1975: 653; Starostin, Dybo & Mudrak 2003: 1015). The stem **ɲeŋde-* means 'serene and frosty (of weather)' in Evenic. (3) Udiheic **gil-či* ~ **gil-si* 'cold (of objects)' vs. **xiŋüː-n-* 'cold (of weather)'. (4) Manchu *šaxuːrun* 'cold', applicable to both objects and weather, theoretically it might be cognate to Evenki *čig-* 'to freeze, get cold' (Tsintsius 1977: 389, 423; Starostin, Dybo & Mudrak 2003: 1336), although the guttural correspondence is irregular. It is more likely that Manchu *šaxuːrun* 'cold' is a local derivation from Manchu *šaxuː-n* 'whitish, pale, dull white'. Out of all these forms, **gil-či* seems the most appropriate candidate since its root is not attested elsewhere and therefore the derivation can be ancient.

Middle Korean. *čʰʌ́-* (Starostin, Dybo & Mudrak 2003: 436).

Proto-Japonic. **túm-* (Starostin, Dybo & Mudrak 2003: 1385). Attested only in Japanese proper (without Ryukyuan). This root, applicable to objects (such as 'water', etc.) is commonly opposed to Proto-Japonic **sàmù-* 'cold (of weather)', which is the item selected for the wordlist in Vovin 1999: 87. Our semantic definitions require the choice of **túm-* rather than **sàmù-*. In Ryukyuan, the old equivalent was probably replaced by **peye-si-* (Thorpe 1983: 273), transparently derived from the Proto-Japonic verb **pìyá-* 'to freeze' (Starostin, Dybo & Mudrak 2003: 348).

16. 'to come'

Proto-Turkic. **geyl* (Sevortyan et al. 1974–: vol. 3: 14; Dybo 2013: 205), retained in all subgroups.

Proto-Mongolic. **ire* (Nugteren 2011: 376; Gruntov & Mazo 2015: 223), attested as a basic term in all three subgroups.

Proto-Tungusic. An unclear situation with two verbs in a criss-crossed configuration (Kazama 2003: 106). (1) **ǯi-* ~ **di-* (Tsintsius 1975: 255; Starostin, Dybo & Mudrak 2003: 1536), a basic term in Manchu-Jurchen and Nanaic (Ulch, Nanai), probably not attested in other languages. (2) **eme-* (Tsintsius 1977: 452; Starostin, Dybo & Mudrak 2003: 503), a basic term in Evenic and some Udiheic lects (Udihe, Oroch), probably not attested in other languages. If Manchu-Jurchen is indeed the first outlier, **ǯi-* ~ **di-* has the advantage, but it is more prudent to treat **ǯi-* ~ **di-* and **eme-* as technical synonyms.

Middle Korean. *ó-* (Starostin, Dybo & Mudrak 2003: 1050).

Proto-Japonic. **kə̀-* (Starostin, Dybo & Mudrak 2003: 538), **kò-* (Vovin 1999: 87). Attested in Japanese and Ryukyuan.

Etymological notes. Starostin, Dybo & Mudrak 2003: 58, 538 treat Turkic **geyl* and Japonic **kə̀-* as cognates < Proto-Altaic **gèle*, assuming an irregular loss of *l* in the Proto-Japonic verbal paradigm. This is not

excluded, but in light of the rarity of this correspondence it may be more prudent to keep these forms apart. Robbets (2005: 385; Robbeets & Bouckaert 2018), however, accepts the Turkic-Japonic comparison.

17. 'to die'

Proto-Turkic. **öl* (Sevortyan et al. 1974–: vol. 1: 525; Dybo 2013: 208), retained in all subgroups.

Proto-Mongolic. **üke* (Nugteren 2011: 540; Gruntov & Mazo 2015: 223), attested in all three subgroups, although usually with impolite connotations, whereas the neutral meaning 'to die' is expressed by various euphemistic new formations.

Proto-Tungusic. **bu-* (Kazama 2003: 103; Tsintsius 1975: 98–99; Starostin, Dybo & Mudrak 2003: 386), attested as a basic term in all four subgroups. The variant *bur-* observed in some paradigmatic forms in certain languages can hardly be reconstructed as the original shape of the Proto-Tungusic root (*pace* Starostin, Dybo & Mudrak 2003) since such an almost total loss of **-r* is unmotivated and unparalleled in the Tungusic languages. This *-r* should rather be analyzed as an occasional suffixal extension used to eliminate an uncommon *CV*-type root structure.

Middle Korean. *čùk-* (Starostin, Dybo & Mudrak 2003: 450).

Proto-Japonic. **sín-* (Starostin, Dybo & Mudrak 2003: 1292), **sin-* (Vovin 1999: 87). Attested in Japanese and Ryukyuan.

18. 'dog'

Proto-Turkic. **iyt* (Sevortyan et al. 1974–: vol. 1: 385; Tenishev 2001: 188; Dybo 2013: 211), retained in all subgroups except for South Siberian.

Proto-Mongolic. **nokai* (Nugteren 2011: 462; Gruntov & Mazo 2015: 223), attested as a basic term in all three subgroups. Theoretically might be analyzed as **no-kai* with a non-productive desemantized suffix **-kai* (Nugteren 2011: 261).

Proto-Tungusic. **ŋinda* (Kazama 2003: 35; Tsintsius 1975: 661–662; Starostin, Dybo & Mudrak 2003: 1029), attested as a basic term in all four subgroups.

Middle Korean. *kàhí* (Starostin, Dybo & Mudrak 2003: 645).

Proto-Japonic. **ìnù* (Starostin, Dybo & Mudrak 2003: 1029, Vovin 1999: 87). Attested in Japanese and Ryukyuan.

Etymological notes. Turkic **iyt*, Tungusic **ŋinda*, Japonic **ìnù* < Proto-Altaic **ŋìndó* (Starostin, Dybo & Mudrak 2003: 1029), formally correspondences are regular, although additional supporting instances for **-nd-* collected by S. Starostin et al. are scant. Robbeets (Robbeets & Bouckaert 2018), reconstructs the Proto-Tungusic form as **ina*, connecting it with the Japonic form.

19. ‘to drink’

Proto-Turkic. **ič* (Sevortyan et al. 1974–: vol. 1: 391; Dybo 2013: 216), retained in all subgroups.

Proto-Mongolic. **uhu* (Nugteren 2011: 536; Gruntov & Mazo 2015: 223), attested as a basic term in all three subgroups.

Proto-Tungusic. **umi-* (Kazama 2003: 94; Tsintsius 1977: 266; Starostin, Dybo & Mudrak 2003: 1499), attested as a basic term in all four subgroups.

Middle Korean. *màs-í-* (Starostin, Dybo & Mudrak 2003: 1499).

Proto-Japonic. **nə̀m-* (Starostin, Dybo & Mudrak 2003: 877), ш (Vovin 1999: 87). Attested in Japanese and Ryukyuan.

Etymological notes. According to Starostin, Dybo & Mudrak 2003: 1499, Tungusic **umi-,* Korean *mà-sí-* < Proto-Altaic **umV* (correspondences are regular except for the dropping of the initial vowel in *mà-sí-* which, however, sporadically occurs in Korean). From the methodological point of view, however, we prefer to adhere to the internal Korean etymology of *màsí-* and treat it as a denominative stem *màs-í-*. Note that the additional difficulty with S. Starostin et al.’ *mà-sí-* is that it implies a zigzag development of the meaning of the Korean root: Proto-Altaic generic ‘to eat’ > Proto-Korean polite ‘to eat’ > Middle Korean generic ‘to eat’.

20. ‘dry’

Proto-Turkic. **kuːr* (Sevortyan et al. 1974–: vol. 6: 154; Dybo 2013: 218). The nominal (adjectival) stem **kuːr* is only retained as relicts in the Turkic lects and never with the basic function ‘dry (of things)’: Chuvash *xɵr* ‘dry (of things)’ in some fixed collocations (Skvortsov 1985: 547), Ancient Oghuz *qur* ‘dry’, Kyrgyz *qur* ‘vain, ungrounded’, Turkish dial. *kur* ‘hard dry soil’. The denominative verb **kurɨ* ‘to become dry’ is more widespread (Chuvash and some Nuclear Turkic subgroups). The majority of synchronic expressions for ‘dry’ in the Turkic languages represent various suffixed stems derived from this stem (Dybo 2013: 218).

Proto-Mongolic. **kahuray* (Nugteren 2011: 434; Gruntov & Mazo 2015: 223), attested as a basic term in Northern, Southern (East Yugur) and Dagur. The second candidate is **kohu-sun* (Nugteren 2011: 416; Gruntov & Mazo 2015: 224), attested as the basic term ‘dry’ in the majority of the Southern lects, apparently a new formation regularly derived from the Proto-Mongolic verb **kohu* ‘to dry (intr.)’ (Nugteren 2011: 416). It is possible that the phonetically similar Common Mongolic adjective **kohu-sun* ‘empty’ attested in all three subgroups (Nugteren 2011: 417) represents a more archaic derivative from the same verb, although the details are not clear.

Proto-Tungusic. **xolga-* ‘to dry (intr.)’ (Tsintsius 1977: 12–13; Starostin, Dybo & Mudrak 2003: 834), a stable Tungusic verb from which various participle stems with the adjectival function ‘dry’ are attested in all four subgroups.

Middle Korean. *mʌ̀rʌ̀-* (Starostin, Dybo & Mudrak 2003: 909).

Proto-Japonic. **kàwà-(ra)-k-* (Starostin, Dybo & Mudrak 2003: 801), **káw(V)rá-k-* (Vovin 1999: 87). Attested in Japanese and Ryukyuan.

Etymological notes. Turkic **kuːr*, Tungusic **kahuray* < Proto-Altaic **kʰiówarV*. In Starostin, Dybo & Mudrak 2003: 801, reconstructed as **kʰióbarV*, but the weakened reflexes of the medial labial point to something like **-w-* rather than normal **-b-*, see Starostin, Dybo & Mudrak 2003: 36 where such cases and the possibility of Proto-Altaic **-w-* are discussed. S. Starostin et al. add Japonic **kàwà-* here, but since *-ra-* is clearly suffixal in light of Old Japanese *kawa-ku*, etc., it might be more prudent to keep the Japonic form apart. Despite the irregular sound correspondence, Robbeets 2005: 339 accepts the whole Turkic-Tungusic-Japonic etymology.

21. 'ear'

Proto-Turkic. **kulga-k* (Sevortyan et al. 1974–: vol. 6: 124; Tenishev 2001: 204; Dybo 2013: 222), retained in all subgroups. Can be analyzed as a deverbative **kul-ga(-)k* from an unattested verb with the common deverbal suffix **-gak* (Räsänen 1957: 125).

Proto-Mongolic. **čiki-n* (Nugteren 2011: 302; Gruntov & Mazo 2015: 224), attested as a basic term in all three subgroups.

Proto-Tungusic. **sian* (Kazama 2003: 17; Tsintsius 1977: 70–71; Starostin, Dybo & Mudrak 2003: 1517), attested as a basic term in all four subgroups.

Middle Korean. *kúy* (Starostin, Dybo & Mudrak 2003: 847).

Proto-Japonic. **mìmì* (Starostin, Dybo & Mudrak 2003: 895, Vovin 1999: 87). Attested in Japanese and Ryukyuan.

Etymological notes. According to Starostin, Dybo & Mudrak 2003: 847, the Turkic stem is to be analyzed as **kul-gak* and compared with Korean *kuy* < Altaic **kʰùːylu* (the cluster **yl* was introduced to explain Korean **y* instead of expected *r*), further to Mongolic **kul(i)-ki* 'earwax; middle ear'. This is not excluded, but since the analysis **kul-gak* implies a verbal status of the original Turkic root, we do not accept Turkic **kulgak* and Korean *kuy* as a full-fledged lexicostatistic match. Robbeets (Robbeets & Bouckaert 2018) accepts the Turkic-Mongolic comparison.

22. 'earth'

Proto-Turkic. **topra-k* (Tenishev 2001: 99; Dybo 2013: 229), retained as a basic term for 'earth (soil)' in all subgroups except for Yakut-Dolgan. Distinct from the stable Proto-Turkic stem **yẹr* 'earth as land, place or ground' (Sevortyan et al. 1974–: vol. 4: 191; Tenishev 2001: 53; Dybo 2013: 227), retained in all subgroups.

Proto-Mongolic. **sirahu* (Nugteren 2011: 492; Gruntov & Mazo 2015: 224), attested as ‘earth (soil)’ in Northern and Southern; Dagur *baləg* ‘soil’ can be of Turkic origin. Distinct from Common Mongolic *gaǯar* ‘earth (territory)’ (Nugteren 2011: 336; Gruntov & Mazo 2015: 224).

Proto-Tungusic. An unstable concept. The best candidate is **turV* (Tsintsius 1977: 217–218; Starostin, Dybo & Mudrak 2003: 1465) meaning ‘earth (soil, territory)’ in Evenic (Even, Negidal, Evenki dialects) with the suffixed derivative *tur-qa* ‘lump of earth’ in Nanai, the latter form proves that the semantics ‘soil’ is ancient enough. For the Nanaic and Udiheic subgroups, **na:* (Tsintsius 1975: 572–573; Starostin, Dybo & Mudrak 2003: 962) can be safely reconstructed as a generic term for ‘earth’ which includes soil, territory, ground, Earth, etc. In Manchu-Jurchen and Evenic (Negidal), **na:* has a narrower meaning: ‘earth (territory)’ (e.g., ‘land’, ‘field’), thus ‘earth (territory)’ could be a Proto-Tungusic meaning of **na:* later expanded in Nanaic and Udiheic. Manchu-Jurchen **bia-gun* with the general meaning ‘earth, ground, soil’ (Tsintsius 1975: 89; Starostin, Dybo & Mudrak 2003: 322) is a derivative from **bia* ‘place (in a dwelling)’ (Tsintsius 1975: 78; Starostin, Dybo & Mudrak 2003: 322) and thus also looks like an innovation. Besides the aforementioned **na:*, note the second Proto-Tungusic term for ‘earth (territory), place’: **buga* (Tsintsius 1975: 100; Starostin, Dybo & Mudrak 2003: 347). Also note **tuka-(la)* (Tsintsius 1977: 207) which should be reconstructed with the meaning ‘clay’ (or ‘mud’?), but in some lects (Evenki, Negidal, Nanai) it acquires the meaning ‘soil’ at least as a secondary synonym.

Middle Korean. *hʌ̀rk* (Starostin, Dybo & Mudrak 2003: 1269).

Proto-Japonic. **tùtì* (Starostin, Dybo & Mudrak 2003: 1423, Vovin 1999: 87). Attested only in Japanese proper. The main Ryukyuan equivalent for this concept is **mita* (Thorpe 1983: 281), possibly connected with Old Japanese *ni* ‘earth, clay', *ni-ta* ‘soggy ground’ through dissimilation, implying a semantic widening (‘sticky / soggy soil’ > ‘soil’ in general).

Etymological notes. Mongolic **sirahu*, Korean *hʌ̀rk* < Proto-Altaic **syárʸi* (Starostin, Dybo & Mudrak 2003: 1269). Correspondences seem regular, assuming fossilized velar suffixes in Mongolic and Korean. Robbeets does not consider this etymology.

23. ‘to eat’

Proto-Turkic. **ye:y* (Sevortyan et al. 1974–: vol. 1: 333; Dybo 2013: 234), retained in all subgroups.

Proto-Mongolic. **ide* (Nugteren 2011: 374; Gruntov & Mazo 2015: 225), attested as a basic term in all three subgroups.

Proto-Tungusic. **ǯep-* (Kazama 2003: 93; Tsintsius 1975: 279–280; Starostin, Dybo & Mudrak 2003: 1530), attested as a basic term in all four subgroups.

Middle Korean. *mə̀k-* (Starostin, Dybo & Mudrak 2003: 950).

Proto-Japonic. **kùp-* (Starostin, Dybo & Mudrak 2003: 667), **kup-* (Vovin 1999: 87). Attested in Japanese and Ryukyuan. Modern Japanese *tabe-ru* is a well-known semantic innovation from the meaning ‘to present, offer’.

Etymological notes. In Starostin, Dybo & Mudrak 2003: 1530, Turkic _*ye:y_ and Tungusic _*ǯep-_ are taken as cognates < Altaic _*ǯe:_ with a unique suffix _*-p-_ in Tungusic _*ǯe-p-_. We prefer to treat these forms as unrelated at the current stage of research. Robbeets does not accept this etymology as well.

24. 'egg'

Proto-Turkic. _*yɨmur-tga_ (Sevortyan et al. 1974–: vol. 4: 250; Tenishev 2001: 149; Dybo 2013: 238), retained in all subgroups. Apparently contaminated with _*yum-ar-(lak)_ 'round 3D' (q.v.). Note that the observed vowel reflexes prevent the direct derivation from _*yum-ar-(lak)_ 'round 3D', despite the typological plausibility of such a scenario.

Proto-Mongolic. _*emdüge-n_ (Nugteren 2011: 473; Gruntov & Mazo 2015: 225), attested as a basic term in all three subgroups. Can be analyzed as _*emdü-ge-n_ with a common nominal suffix.

Proto-Tungusic. _*umu:-kta_ ~ _*umu:-kan_ (Kazama 2003: 29; Tsintsius 1977: 269; Starostin, Dybo & Mudrak 2003: 1499), two different derivatives (_*-kta_ in Evenic, Nanaic, Udiheic, _*-kan_ in Manchu-Jurchen) from the verb _*umu:-_ 'to lay eggs' (Tsintsius 1977: 269; Starostin, Dybo & Mudrak 2003: 1498) retained in Evenki. Note that theoretically Evenki _umu:-_ 'to lay egg' can be a back-formation from _umu:-kta_ 'egg'. According to Starostin, Dybo & Mudrak 2003: 599, to be separated from _*umu-_ 'nest, burrow', although etymological connections between 'egg' and 'nest' are common cross-linguistically.

Middle Korean. _àrh_ (Starostin, Dybo & Mudrak 2003: 490).

Proto-Japonic. There is no separate Japanese root with the meaning ʽegg'; in most occurrences, this meaning was likely expressed by the word _*kúa_ (Starostin, Dybo & Mudrak 2003: 742), _*kwo_ (Vovin 1999: 87) ʽchild, offspring' or various secondary compounds containing this stem (_*kápí-kúa_, lit. ʽshell-child', _*táma-kúa_, lit. ʽball-child'). The common Ryukyuan equivalent is _*ko-ga_, where _*-ga_ is a diminutive suffix (Thorpe 1983: 282).

Etymological notes. Mongolic _*emdü(-)ge-n_ and Tungusic _*umu:-kta_ ~ _*umu:-kan_ can be cognates < Altaic _*úmu-(tki)_, thus Starostin, Dybo & Mudrak 2003: 1499. Morphological details are not entirely clear, but the comparison seems acceptable, especially if we assume a fossilized suffix _*-d-_ in Mongolic and some secondary morphological processes in Tungusic. In Starostin, Dybo & Mudrak 2003: 1499, Turkic _*yɨmur-tga_ 'egg' is also adduced here with initial _*y-_ under the influence of _*yum-ar-(lak)_ 'round'. This is indeed not excluded, but the unprovable assumption of contamination should formally prevent us from regarding the Turkic form as cognate. Robbeets does not consider this etymology.

25. 'eye'

Proto-Turkic. _*gör-s_ (Sevortyan et al. 1974–: vol. 3: 60; Tenishev 2001: 209; Dybo 2013: 241), retained in all subgroups. An old derivative from the verb _*gör_ 'to see' (q.v.).

Proto-Mongolic. _*nidü-n_ (Nugteren 2011: 459; Gruntov & Mazo 2015: 225), attested as a basic term in all three subgroups. Theoretically can be analyzed as _*ni-dü-n_ with the rare desemanticized suffix _*-du(-)n_.

The underlying idea that the starting Pre-Proto-Mongolic form was **nil-dün*, allegedly cognate to **nil-busun ~ *nil-musun* 'tear', is not likely, however, because **nilbusu-n ~ *nilmusu-n* actually means 'tear, saliva, mucus' (Nugteren 2011: 457) being apparently a regular derivative from **nilbu* 'to spit'. Cf. the similar cases: **hodu-n* (< **hol-du-n*?) 'star', **modu-n* (< **mo(r)-du-n*?) 'tree', **sidü-n* (< **sil-dü-n*?) 'tooth'.

Proto-Tungusic. **iasa* (Kazama 2003: 16; Tsintsius 1975: 291–292; Starostin, Dybo & Mudrak 2003: 981), attested as a basic term everywhere (in the majority of lects, the old plural form with the ending *-l* is used). The protoform is reconstructed as **ɲiasa* by S. Starostin et al., but Doerfer 1995: 252–253 plausibly claims that the modern nasal onset forms (with *n-, ɲ- or ŋ-*) are restricted to the compact area of Central Nanai dialects and thus should be considered an inner Nanai innovation (on the other hand, note that the emergence of the Central Nanai nasal forms lacks convincing explanation). There are also some pieces of evidence that the Jurchen word for 'eye' is to be read with a nasal onset (Dybo & Starostin 2008: 224–227), at the moment, however, we accept Vovin's (2005: 83–84) criticism and transcribe the Jurchen with initial *ya-*. Both Starostin, Dybo & Mudrak 2003: 981 and Doerfer 1995: 252–253 single out a rare desematicized suffix **-sa* in this stem, although with different argumentation.

Middle Korean. *nún* (Starostin, Dybo & Mudrak 2003: 981).

Proto-Japonic. **mài-(N)* (Starostin, Dybo & Mudrak 2003: 981), **mà-n* (Vovin 1999: 87). Attested in Japanese and Ryukyuan. The only evidence for a final nasal comes from Ryukyu (forms such as Hateruma *miŋ*), but it is so scarce that even Thorpe reconstructs just **me* for Proto-Ryukyuan (Thorpe 1983: 284).

Etymological notes. Starostin, Dybo & Mudrak 2003: 981 reconstruct Proto-Altaic **n̩ià̯:* which yields Mongolic **nidü-*, Tungusic **iasa ~ *ɲiasa*, Korean *nún*, Japonic **mài*. Such an analysis is not excluded, if one assumes (unique) fossilized suffixes modifying a *CV*-root. We prefer, however, to keep these forms apart. Especially note that the Tungusic form is likely to be reconstructed as **iasa* without nasal onset which makes the Tungusic *comparandum* even more dubious. Robbeets (2005: 250, 267) rejects the etymological connection of Japanese *me* 'eye' with the aforementioned Altaic forms.

26. 'fat'

Proto-Turkic. **ya:g* (Sevortyan et al. 1974–: vol. 4: 58; Tenishev 2001: 453; Dybo 2013: 248), attested as a basic term in Chuvash and Nuclear Turkic (e.g., in Yakut and Tofa-Tuvinian).

Proto-Mongolic. **ehükü-n* (Nugteren 2011: 334; Gruntov & Mazo 2015: 225), attested as a basic term in all three subgroups.

Proto-Tungusic. The most widespread term is **ximu:-kse* (Tsintsius 1975: 313–314; Starostin, Dybo & Mudrak 2003: 800), meaning 'fat (in general)' or more widely 'fat, oil' in Evenic, Nanaic and Udiheic and '(vegetable) oil' in Manchu-Jurchen. The stem is a deverbative from **ximu:-* 'to smear (with fat); to melt' attested in Evenic, Nanaic and Udiheic. The second candidate is the root **nim-* (Tsintsius 1975: 314, 594, 595; Starostin, Dybo & Mudrak 2003: 978), from which various derivatives are attested, first of all, **nimu-kse* > Manchu *niməŋgi* 'fat' (probably a result of contamination with Manchu *iməŋgi* 'oil' < **ximu:-kse*) and **nim-ne* > 'intestines, intestinal contents' in Evenki, 'intestinal fat' in Even. It is not excluded that

Manchu retains the original opposition between ‘fat’ and ‘oil, liquid fat’, i.e., **nim-* was actually a Proto-Tungusic root for ‘fat (in general)’ sparsely retained in modern languages having been absorbed with the deverbative **ximu:-kse* ‘oil, liquid (rendered) fat’ after the Manchu split-off, but formally **ximu:-kse* is a more appropriate Proto-Tungusic candidate for this meaning.

Middle Korean. *kɨ̀rɨ́m* (Starostin, Dybo & Mudrak 2003: 800).

Proto-Japonic. **àmpùr-à* (Tower of Babel project), **à(n)púrá* (Vovin 1999: 87). Attested in Japanese and Ryukyuan. Probably derived from **ampu-r-* ‘to roast’.

Etymological notes. Turkic **ya:g*, Mongolic **ehü(-)kü-n* < Proto-Altaic **iá:gi* (Starostin, Dybo & Mudrak 2003: 597). Correspondences are formally regular, although the reflexes of the assumed Altaic diphthong are tangled and the instances are scant. Additionally, Starostin, Dybo & Mudrak 2003: 800 reconstruct Proto-Altaic **k^{h}yár^{y}me* > Tungusic **ximu:-kse*, Korean *kɨ̀rɨ́m*. We prefer to reject this etymology since, first, the specific cluster *r^{y}m* is weakly supported by evidence and, second, in the case of both forms, Tungusic **ximu:-kse* and Korean *kɨ̀rɨ́m*, we are probably dealing with old deverbatives. Robbeets does not consider this etymology.

27. ‘feather’

Proto-Turkic. There are two main candidates. The first one is **tü:k* (Tenishev 2001: 150; Dybo 2013: 258), attested as a basic term for ‘feather’ in Chuvash and Nuclear Turkic (Yakut, Tofa-Tuvinian, Oghuz, Kipchak, Altay). It shows polysemy ‘feather / down / fur’ in both Chuvash and the majority of Nuclear Turkic lects. The second candidate is **yüg* (Tenishev 2001: 197; Dybo 2013: 259) attested as ‘feather’ in general or specifically as ‘flight feather, quill’ in some Nuclear Turkic subgroups: Tuvinian, South Siberian (Khakas, Shor, Western Yugur), Turkish dialects as well as in some Ancient Turkic sources: Karakhanid, Middle Kipchak, Old Ottoman (Clauson 1972: 910). Such a distribution of **yüg* probably excludes an areal origin of this root. Since the shift ‘fur’ > ‘down’ > ‘feather’ is common cross-linguistically, the most probable scenario is the Proto-Turkic opposition **yüg* ‘feather’ / **tü:k* ‘fur, down’, with latter **tü:k* latter expanding its meaning towards the generic semantics ‘fur, down, feather’ independently or under contact influence in many subgroups including Chuvash.

Proto-Mongolic. **hödü-n* (Nugteren 2011: 360; Gruntov & Mazo 2015: 225), attested as a basic term in all three subgroups. Theoretically can be analyzed as **hö-dü-n* with the rare desemantized suffix **-du(-)n*.

Proto-Tungusic. **dek-te* (Tsintsius 1975: 231; Starostin, Dybo & Mudrak 2003: 468), attested as a basic term at least in Evenic, Nanaic and Udiheic (usually modified with desemanticized suffixes and with polysemy ‘feather / wing’, sometimes only as ‘wing’). In Manchu, *detxe* is glossed as ‘pinion; arrow feathers’, but such collocations as, e.g., *xexe detxe* ‘the smaller feathers on a bird’s wing’, lit. ‘female feathers’ prove that Manchu *detxe* meant simply ‘feather’ in the recent past. Apparently derived from **deg-* ‘to fly’ q.v. thus to be analyzed as a suffixed stem **dek-te*.

Middle Korean. *číč^h* (Starostin, Dybo & Mudrak 2003: 1335).

Proto-Japonic. **pánái* (Starostin, Dybo & Mudrak 2003: 1186), **pánÉ* (Vovin 1999: 88). Attested in Japanese and Ryukyuan.

Etymological notes. Turkic **yüg*, Tungusic **dek-te* < Proto-Altaic **dégì* (Starostin, Dybo & Mudrak 2003: 468). Correspondences are regular except for Turkic **-ü-* instead of expected **-e-*. Robbeets does not consider this etymology.

28. 'fire'

Proto-Turkic. **hoːt* (Sevortyan et al. 1974–: vol. 1: 483; Tenishev 2001: 356; Dybo 2013: 262), retained in all subgroups.

Proto-Mongolic. **gal* (Nugteren 2011: 337; Gruntov & Mazo 2015: 226), attested as a basic term in all three subgroups.

Proto-Tungusic. **toga* (Kazama 2003: 44; Tsintsius 1977: 190; Starostin, Dybo & Mudrak 2003: 1450), attested as a basic term in all four subgroups.

Middle Korean. *pɨr* (Starostin, Dybo & Mudrak 2003: 1172).

Etymological notes. Korean *pɨr* and Japonic **pə̀-i* as cognates < Proto-Altaic **pʰòre* (Starostin, Dybo & Mudrak 2003: 1172). Despite the fact that loss of medial *-r-* in Japonic seems irregular, the Japonic-Korean pair is widely accepted by experts as a likely cognate match (see Robbeets 2005: 404; Robbeets & Bouckaert 2018 for references); even Vovin (2010: 107), known for his skeptical position on Japonic-Korean relationship, acknowledges the correspondences between the two forms as regular. Various explanations for the absence of *-r-* in the Japonic form have been proposed; e.g., S. Starostin et al. assume a *gV*-suffix in Pre-Proto-Japonic with further simplification of the cluster *-rg-* followed by regular deletion of *-g-*.

29. 'fish'

Proto-Turkic. **baːlɨk* (Sevortyan et al. 1974–: vol. 2: 59; Tenishev 2001: 177; Dybo 2013: 266), retained in all subgroups.

Proto-Mongolic. **ǯiga-sun ~ *ǯigal-sun* (Nugteren 2011: 379; Gruntov & Mazo 2015: 226), attested as a basic term in all three subgroups.

Proto-Tungusic. Each subgroup has its own candidate for the status of the Proto-Tungusic term for 'fish'. (1) **xolsa* (or **xol-sa* with a rare desemanticized suffix?) (Kazama 2003: 34; Tsintsius 1977: 14; Starostin, Dybo & Mudrak 2003: 848), meaning 'fish' in Evenic and 'boiled fish' in Nanaic and Udiheic. (2) **sugǯa-nsa* (Kazama 2003: 34; Tsintsius 1977: 118–119; Starostin, Dybo & Mudrak 2003: 1316), meaning 'fish' in Nanaic and Udiheic and 'salmon' in Evenic. (3) **liamba-xa* (Kazama 2003: 34; Tsintsius 1975: 496; Starostin, Dybo & Mudrak 2003: 874), meaning 'fish' in Manchu-Jurchen as well as in Kilen and some Nanai dialects, derived from **liamba* '(a k. of) salmon' attested in Evenic and Nanaic. Topologically **li-amba-xa* has the advantage since it is attested in two subgroups, but its transparent derivative nature and

very scant attestation outside Manchu-Jurchen suggest that **liamba-xa* might be a new formation and that the Nanai form is actually a Manchu loan. It is not easy to make a choice between **xolsa* and **sugǯa-nsa*. The advantage of the stem **sugǯa-nsa* is that its generic meaning 'fish' is attested in two groups (Nanaic and Udiheic). Note that its meaning 'salmon' in Evenic gives no hint, since the semantic shift between 'fish' and a name of the fish species basic for the given region can happen in both directions. Differently with **xolsa* and its two meanings 'fish' and 'boiled fish': in this case only the direction 'fish' > 'boiled fish' seems typologically normal, not *vice versa*. Since Nanaic and Udiheic (where **xolsa* means 'boiled fish') either form a distinct clade or heavily influence each other, the most probable scenario is that the Proto-Tungusic term for 'fish' was **xolsa* which was retained in Evenic, shifted towards the specific meaning 'boiled fish' in Nanaic-Udiheic, and was lost in Manchu-Jurchen.

Middle Korean. *kòkí* (Starostin, Dybo & Mudrak 2003: 734). The same word as 'meat' q.v.; a Late Middle Korean introduction for the more archaic root $t^{h}i$ 'fish' (Vovin 2000: 147–148).

Proto-Japonic. **íwuá* ~ **díwuá* (Starostin, Dybo & Mudrak 2003: 477), **(d)íwó* (Vovin 1999: 88). Attested in Japanese and Ryukyuan. No known historic or dialectal variant of Japonic distinguishes between the onsets **i-* and **di-* (**yi-* in the *y*-version of the reconstruction).

Etymological notes. Mongolic **ǯiga-*, Korean $t^{h}i$, Japonic **íwuá* < Proto-Altaic **di̯ági* (Starostin, Dybo & Mudrak 2003: 477). Correspondences seem regular. Robbeets (Robbeets & Bouckaert 2018) accepts the Mongolic-Japonic comparison.

30. 'to fly'

Proto-Turkic. **uč* (Sevortyan et al. 1974–: vol. 1: 612; Dybo 2013: 269), retained in all subgroups except for Yakut-Dolgan.

Proto-Mongolic. **nis* (Nugteren 2011: 458; Gruntov & Mazo 2015: 226), attested as a basic term in all Northern and Southern. Dagur *dərdə* 'to fly, fly up; to swim' originates from **dehe-re* 'above, upper' (Nugteren 2011: 314) with an additional suffix; the general meaning 'to fly' is obviously secondary here.

Proto-Tungusic. **deg-* (Kazama 2003: 94; Tsintsius 1975: 228–229; Starostin, Dybo & Mudrak 2003: 1359), attested as a basic term in all four subgroups.

Middle Korean. *nʌ̀r-* (Starostin, Dybo & Mudrak 2003: 974).

Proto-Japonic. **tə́mp-* (Starostin, Dybo & Mudrak 2003: 1365), **tónp-* (Vovin 1999: 88). Attested in Japanese and Ryukyuan.

31. 'foot'

Proto-Turkic. **hada-k* (Sevortyan et al. 1974–: vol. 1: 103; Tenishev 2001: 288; Dybo 2013: 272), final **-k* can be singled out as a common nominal suffix. Attested as a basic term for 'foot' (sometimes with polysemy 'foot / leg') in all subgroups except for Tofa-Tuvinian and Altay. Distinct from Proto-Turkic or

at least Proto-Nuclear Turkic *bu:t* ‘leg’ (Sevortyan et al. 1974–: vol. 2: 280; Tenishev 2001: 282; Dybo 2013: 274).

Proto-Mongolic. **köl* (Nugteren 2011: 425; Gruntov & Mazo 2015: 226), attested as a basic term in all three subgroups. Probably to be reconstructed with polysemy ‘foot / leg’.

Proto-Tungusic. We reconstruct the following opposition for Proto-Tungusic: **palga-n* ‘foot’ (Tsintsius 1977: 312; Starostin, Dybo & Mudrak 2003: 1075) vs. **begdi* ‘leg’ (Tsintsius 1975: 118–119; Starostin, Dybo & Mudrak 2003: 380). This opposition is frequently retained in non-Manchu-Jurchen subgroups. In Manchu, it was simplified in favor of **begdi* with polysemy ‘leg / foot’, whereas **palga-n* ‘foot’ was lost. Of course, if Manchu-Jurchen is a first outlier, it is not excluded that actually **begdi* meant ‘leg / foot’ in Proto-Tungusic and **palga-n* is a later introduction after Manchu-Jurchen split off.

Middle Korean. *pár* (Starostin, Dybo & Mudrak 2003: 1075).

Proto-Japonic. **àsì* (Starostin, Dybo & Mudrak 2003: 292). Attested only in Japanese proper, with the neutralized meaning ʻfoot, legʼ. In Ryukyuan, the basic equivalent for the same neutralized meaning is **pagi* (Thorpe 1983: 289) = Old Japanese *pagyi* ʻlower leg, shankʼ, going back to Proto-Japonic **pànkì* for which Vovin (1999: 88) somehow assumes the meaning ʻfootʼ. It may be argued that the best way is to reconstruct the semantic opposition **àsì* ʻfootʼ / **pànkì* ʻlegʼ for Proto-Japonic, with both meanings syncretized independently of each other in Japanese and Ryukyuan.

Etymological notes. Tungusic **palga-n*, Korean *pár* < Proto-Altaic **pàlgà* (Starostin, Dybo & Mudrak 2003: 1075; Robbeets 2005: 400; Robbeets & Bouckaert 2018). Correspondences are regular.

32. ‘full’

Proto-Turkic. **do:l-* (Sevortyan et al. 1974–: vol. 3: 257; Tenishev 2001: 288; Dybo 2013: 278), the verb ‘to be(come) full’ from which various suffixal derivatives with the meaning ‘full’ are used in Turkic languages.

Proto-Mongolic. **dehür-* (Nugteren 2011: 321; Gruntov & Mazo 2015: 227), the verb ‘to be full’ from which participles with the adjectival meaning ‘full’ are derived: **dehür-eŋ* in Northern and Southern and **dehür-ku* in Dagur.

Proto-Tungusic. The verb **ǯalu-* ‘to fill (intr.)’ and its participle **ǯalu-m* (Tsintsius 1975: 247; Starostin, Dybo & Mudrak 2003: 390) are attested as a basic terms in all four subgroups.

Middle Korean. *kʌ̀tʌ̀k-* (Starostin, Dybo & Mudrak 2003: 674).

Proto-Japonic. **mìt-* (Starostin, Dybo & Mudrak 2003: 917, Vovin 1999: 88). Attested in Japanese and Ryukyuan.

Etymological notes. Turkic **do:l-*, Tungusic **ǯalu-* < Proto-Altaic **čá:lo* (Starostin, Dybo & Mudrak 2003: 390). Correspondences seem regular. Robbeets does not consider this etymology.

33. ‘to give’

Proto-Turkic. **bẹ:r* (Sevortyan et al. 1974–: vol. 2: 114; Dybo 2013: 280), attested in all subgroups.

Proto-Mongolic. **ög* (Nugteren 2011: 472; Gruntov & Mazo 2015: 227), attested as a basic term in all three subgroups.

Proto-Tungusic. **bu:-* (Kazama 2003: 108; Tsintsius 1975: 99; Starostin, Dybo & Mudrak 2003: 353), attested as a basic term in all four subgroups.

Middle Korean. *čú-* (Starostin, Dybo & Mudrak 2003: 399).

Proto-Japonic. **átá-pá-* (Starostin, Dybo & Mudrak 2003: 603), **ata[-]pa-Ci* (Vovin 1999: 88). Attested in Japanese proper. Largely replaced in the modern language with innovations such as *kudasa-ru* 'to give to 1st p.' (< 'to lower') and *age-ru* 'to give to 2nd / 3rd p.' (< 'to raise') respectively. In Ryukyuan, the main equivalent for this meaning is **kure* (Thorpe 1983: 290; Starostin, Dybo & Mudrak 2003: 825), whose cognates are also attested in the meaning 'to give to 1st p.' beginning from the Middle Japanese period; lack of attestation in Old Japanese, however, would make this a highly dubious entry in the wordlist. Nevertheless it seems more prudent to keep **átá-pá-* and **kure* as technical synonyms for Proto-Japonic.

Etymological notes. Turkic **bẹ:r*, Tungusic **bu:-* < Proto-Altaic **bió:ré* (Starostin, Dybo & Mudrak 2003: 353). Normally Altaic **-r-* is retained in Tungusic (Starostin, Dybo & Mudrak 2003: 24), but in Starostin, Dybo & Mudrak 2003: 54, several etymologies are offered where Altaic **-r-* is lost in Tungusic after a long vowel, i.e., in the roots of the shape *CV:r-* (e.g., ‘to know’, ‘water’, ‘yellow’ q.v.). Robbeets (Robbeets & Bouckaert 2018) keeps Turkic and Tungusic forms apart, comparing Tungusic **bu:-* with Japonic **(w)ura-* ‘to sell’ and Korean **pʌlʌ-kʌ-* ‘to sell’.

34. ‘good’

Proto-Turkic. The first candidate is **ẹd-gü* (Sevortyan et al. 1974–: vol. 1: 245, 248, 329; Dybo 2013: 282), an adjective derived from the Proto-Turkic substantive **ẹd* ‘thing, property, good’. Attested as a basic term for ‘good’ in some Nuclear Turkic groups: Yakut, Tofa-Tuvinian, Oghuz, Kipchak. The second candidate is **yak-(i)š-i* (Sevortyan et al. 1974–: vol. 4: 63; Dybo 2013: 284), attested as basic ‘good’ in South Siberian, Oghuz, Karluk, Kipchak, Altay, an adjectival deverbative from **yak*, **yak-iš* ‘to near to, approach; to be suitable’ (Sevortyan et al. 1974–: vol. 4: 81). No reliable inherited terms in Chuvash. Both competing stems, **ẹd-gü* and **yak-iš-i*, looks like new formations, although **yak-iš-i* is formed according to a more productive model than **ẹd-gü* which makes **ẹd-gü* a more reliable candidate for the status of the Proto-Turkic (or at least Proto-Nuclear Turkic) term for ‘good’. Nevertheless we prefer to treat **ẹd-gü* and **yak-iš-i* as technical synonyms.

Proto-Mongolic. **sahi-n* (Nugteren 2011: 480; Gruntov & Mazo 2015: 227), attested as a basic term in all three subgroups.

Proto-Tungusic. **aya-* (Kazama 2003: 129; Tsintsius 1975: 18–20; Starostin, Dybo & Mudrak 2003: 279), attested as a basic term in non-Manchu-Jurchen subgroups. In Manchu-Jurchen, this word was superseded with a Mongolian loan, but the root is retained in some derivatives such as ‘beautiful’ and ‘to save from’.

Middle Korean. *tyŏ:h-* (Starostin, Dybo & Mudrak 2003: 478).

Proto-Japonic. **də̀-* (**yə̀-*) (Starostin, Dybo & Mudrak 2003: 478), **dò-* (**yò-*) (Vovin 1999: 88). Attested in Japanese and Ryukyuan.

Etymological notes. Korean *tyŏ:h-*, Japonic **də̀-* < Proto-Altaic **di̯òge* (Starostin, Dybo & Mudrak 2003: 353; Robbeets 2005: 399), correspondences seem regular.

35. 'green'

Proto-Turkic. **gö:k* (Sevortyan et al. 1974–: vol. 3: 66; Tenishev 2001: 60, 604; Dybo 2013: 290), attested as 'green' (frequently with polysemy 'green / blue') in all Nuclear Turkic subgroups and as 'blue' in Chuvash. In order to resolve the polysemy 'green / blue' a new formation **ya:ʎ-ɨl* 'green' from **ya:ʎ* 'fresh, moist, unripe' was introduced in many Nuclear Turkic languages including some Ancient Turkic sources (Sevortyan et al. 1974–: vol. 4: 161; Clauson 1972: 978; Dybo 2013: 289).

Proto-Mongolic. **nogoha-n* (Nugteren 2011: 461; Gruntov & Mazo 2015: 227), attested as a basic term for 'green' in all three subgroups, sometimes with polysemy 'green / (light) blue' as in Dagur. The second candidate is **köke* (Nugteren 2011: 424; Gruntov & Mazo 2015: 227) which more frequently means 'blue', but nevertheless is attested with polysemy 'blue / green' in all three subgroups. Gruntov and Mazo (2015: 228) state that the reflexes of **nogoha-n* in the meaning 'green' are normally applicable to new grass, whereas the reflexes of **köke* in the meaning 'green' are normally applicable to mature grass. According to our general specifications, a term denoting the color of fresh, newly sprung grass, i.e. **nogoha-n*, should be taken. The second argument against **köke* is that it suspiciously resembles Proto-Turkic **gö:k* 'blue; green' and actually might be an early Turkic loan.

Proto-Tungusic. The main candidate is **ɲog-* (Tsintsius 1975: 601–603; Starostin, Dybo & Mudrak 2003: 875), its derivatives mean 'green' in Manchu, Nanaic and Udiheic (Oroch). These are (1) **ɲog-(g)ǯo* 'green; blue' or 'green; blue; yellow' in Nanaic and Udiheic, modified with the common suffix **-gǯV*. (2) **ɲog-aŋgia-(n)* 'green' in Manchu-Jurchen, e.g., Manchu *ɲowaŋgʸan* 'green'. Surprisingly this suffixed stem also occurs in Nanai as *noŋgʸä:(n)* 'green; blue', probably a Manchu loan. The root final **-g-* is revealed by Manchu *ɲowaŋgʸan* 'green' (with **VgV > VwV*), from Oroch suffixless *ɲogo* 'greenish, bluish' (Arsenyev 2008: 88) (besides regular Oroch *ɲogǯo* 'green, blue' < **ɲog-(g)ǯo*), and from such derivatives as Udihe *ɲogbö* 'grass'. In Starostin, Dybo & Mudrak 2003: 875, the root **ɲog-* is reconstructed as **ʎog-* with the unique occurrence of a phoneme **ʎ* in order to compare it with the Even-Evenki stem **lugdV-* 'dark' (Tsintsius 1975: 506, 650), such a comparison, however, is not obligatory, whereas the implied total shift **l > n* (or **ʎ > ɲ* in our case) in all languages except for Evenki looks strange. The second candidate for 'green' is **ču:-ri:-* (Tsintsius 1977: 417; Starostin, Dybo & Mudrak 2003: 454), attested in Evenic as a basic term for 'green; blue'. Further cf. such Evenic stems as *ču-la-* 'blue' or 'blue; green' (Tsintsius 1977: 412), **ču:-l-ba-* 'greenery' (Tsintsius 1977: 412), **ču:-tu:-* 'to be blue (of sky)' (Tsintsius 1977: 418). Since it is unlikely that the aforementioned Evenic stems are unrelated to each other, we postulate a root **ču:-* modified with rare petrified suffixes. No traces of **ču:-* outside Evenic. If Manchu-Jurchen is indeed the first outlier, **ɲog-* has the advantage, but we prefer to treat **ɲog-* and **ču:-* as technical synonyms.

Middle Korean. *pʰɨr-ɨ-* (Starostin, Dybo & Mudrak 2003: 1169).

Proto-Japonic. **àwə-* (Starostin, Dybo & Mudrak 2003: 278), **àwò-* (Vovin 1999: 88). Attested in Japanese and Ryukyuan, with polysemy ʻblue / green' in most dialects.

Etymological notes. Mongolic **nogoha-n*, Tungusic **ɲog-* < Proto-Altaic **nioga* (Starostin, Dybo & Mudrak 2003: 875 with incorrect lambdacized reconstruction: Tungusic **ʎog-* and Altaic **ʎioga*). Correspondences seem regular. Robbeets does not consider this etymology.

36. 'hair'

Proto-Turkic. **sač* (Sevortyan et al. 1974–: vol. 7: 216; Tenishev 2001: 197; Dybo 2013: 296), retained in all subgroups, meaning 'head hair'. Distinct from **kɨlk* 'a single hair' (Sevortyan et al. 1974–: vol. 6: 204; Tenishev 2001: 196; Dybo 2013: 294) and **tü:k* 'fur'.

Proto-Mongolic. **hüsü-n* ~ **hü-sün* (Nugteren 2011: 371; Gruntov & Mazo 2015: 228), attested as a basic term in all three subgroups.

Proto-Tungusic. The lexical opposition **ɲu:ri-kte / *xiɲŋa-(kta)* can be safely reconstructed for non-Manchu-Jurchen. The stem **ɲu:ri-kte* (Tsintsius 1975: 648; Kazama 2003: 16; Starostin, Dybo & Mudrak 2003: 993) means 'head hair' in Evenic and Udiheic and 'hair (probably in general)' in Nanaic. The second word, **xiɲŋa-(kta)* (Tsintsius 1975: 317; Starostin, Dybo & Mudrak 2003: 788), means 'body hair; fur' (optionally also 'fine feathers, down') in Evenic and Udiheic and 'fur' in Nanaic; its Manchu cognate means 'down, fluff'. Thus **ɲu:ri-kte* is a reliable candidate for the Proto-Tungusic meaning 'head hair' or even 'hair (in general)'. Differently in Manchu-Jurchen, where 'hair (in general); fur' is denoted with the help of **puɲe-* (Tsintsius 1977: 303; Starostin, Dybo & Mudrak 2003: 1186), lacking transparent cognates in other subgroups. Since Manchu-Jurchen can be a first outlier, we accept **ɲu:ri-kte* and **puɲe-* as technical synonyms.

Middle Korean. *mərí* (Starostin, Dybo & Mudrak 2003: 910).

Proto-Japonic. **kàmì* (Starostin, Dybo & Mudrak 2003: 760). Attested in Japanese proper; specifically ʻhead hair' as opposed to **kái* ʻbody hair' (Starostin, Dybo & Mudrak 2003: 788 = **ká-Ci* in Vovin 1999: 88, since Vovin takes the meaning ʻbody hair' rather than ʻhead hair' for his wordlist). In Ryukyuan, **ke* (Thorpe 1983: 293) seems to refer to both types of hair, but this is probably the result of semantic neutralization.

Etymological notes. Mongolic **hü-sü*, Tungusic **puɲe-* < Proto-Altaic **pʰúɲe* (Starostin, Dybo & Mudrak 2003: 1186). Correspondences seem regular, see Starostin, Dybo & Mudrak 2003: 62 for Altaic **-ɲ-* > Mongolic *Ø* before the *su*-suffix. Robbeets does not consider this etymology.

37. 'hand'

Proto-Turkic. **elg* (Sevortyan et al. 1974–: vol. 1: 260; Tenishev 2001: 251; Dybo 2013: 303), attested as a basic term in Chuvash and a number of Nuclear Turkic subgroups (Yakut, Oghuz, Karluk) including

Ancient Turkic sources; in other Nuclear Turkic subgroups, its derivative ‘mitten’ has survived. Distinct from **kol* ‘arm’ (Sevortyan et al. 1974–: vol. 6: 37; Tenishev 2001: 244; Dybo 2013: 305).

Proto-Mongolic. **gar* (Nugteren 2011: 337; Gruntov & Mazo 2015: 228), attested as a basic term in all three subgroups.

Proto-Tungusic. **ŋaːla* (Tsintsius 1975: 656–657; Kazama 2003: 19; Starostin, Dybo & Mudrak 2003: 1024), attested as a basic term in all four subgroups. Doerfer 1995: 254 claims that this stem is to be analyzed as **ŋaː-la* with the same root as in the verb **ŋaː-di-* meaning ‘to call (by voice)’ in Udiheic and ‘to call (by voice or hand movement)’ in Evenic, but such a solution does not seem obligatory.

Middle Korean. *sòn* (Starostin, Dybo & Mudrak 2003: 1520).

Proto-Japonic. **tà-i* (Starostin, Dybo & Mudrak 2003: 1351), **tà-Ci* (Vovin 1999: 88). Attested in Japanese and Ryukyuan.

Etymological notes. Turkic **elg*, Tungusic **ŋaːla* < Proto-Altaic **ŋàːli* (Starostin, Dybo & Mudrak 2003: 1024). Correspondences seem regular, assuming a fossilized *g*-suffix in Turkic. Robbeets does not consider this etymology.

38. ‘head’

Proto-Turkic. **baʎč* (Sevortyan et al. 1974–: vol. 2: 85; Tenishev 2001: 194; Dybo 2013: 315), retained in all subgroups.

Proto-Mongolic. **heki-n* (Nugteren 2011: 352; Gruntov & Mazo 2015: 228), meaning ‘head (anatomic)’ in Southern (Middle Mongolic, Mogholi), Dagur and as a relict in some fixed expressions in Oirat (Northern group) (Gruntov & Mazo 2015: 229); this stem is also attested in many Mongolic lects in such metaphoric meanings as ‘beginning’, ‘source’. The antiquity of the anatomic semantic for **heki-n* may be argumented by four facts: (1) it is attested in all the three subgroups; (2) it is attested in ancient sources; (3) it is attested in geographically peripheral modern languages (Mogholi, Oirat, Dagur) around the core Mongolic area; (4) the semantic shift ‘head’ > ‘beginning’ is much more common than vice versa. The second candidate is **tolu-gai* (Nugteren 2011: 522; Gruntov & Mazo 2015: 229), widely attested with the anatomic meaning ‘head’ in the Northern subgroup as well as in some adjacent Southern lects. This seems a trivial lexical replacement of the Northern subgroup which has later spread onto the contacted Southern lects. The third candidate is **terihu-n* (Nugteren 2011: 519; Gruntov & Mazo 2015: 229), widely attested with the anatomic meaning ‘head’ in the Southern subgroup including Middle Mongolic sources. The common Mongolic meaning of **terihu-n* is, however, ‘first; chief; beginning’ as attested in all three subgroups. Despite the typological rarity of the semantic development ‘first, beginning’ > ‘head’, it is most likely that the anatomic semantic of **terihu-n* is an innovation of the Southern subgroup - possibly of areal origin (in light of the fact that **heki-n* is also attested as ‘anatomic head’ in Middle Mongolic).

Proto-Tungusic. The first candidate is **dili* (Tsintsius 1975: 205–206; Kazama 2003: 16; Starostin, Dybo & Mudrak 2003: 476), attested as a basic term in non-Manchu-Jurchen subgroups, but in Manchu it means ‘the base of the horn on deer, roe’. The second candidate is **irgü* (Tsintsius 1975: 326; Starostin, Dybo &

Mudrak 2003: 622) which means ‘head’ in Manchu-Jurchen and ‘brain’ in other subgroups. If Manchu-Jurchen is indeed a first outlier both stems are equally appropriate. We accept them as technical synonyms.

Middle Korean. *mərí* (Starostin, Dybo & Mudrak 2003: 910).

Proto-Japonic. **kàsìrà* (Starostin, Dybo & Mudrak 2003: 660, Vovin 1999: 88). Attested in Japanese proper. In Ryukyuan dialects, the meaning is highly unstable and no single Proto-Ryukyuan equivalent can be easily reconstructed.

Etymological notes. Turkic **baʎč*, Korean *mərí* < Proto-Altaic **méʎʒu* (Starostin, Dybo & Mudrak 2003: 910). Correspondences seem regular. Robbeets does not consider this etymology.

39. ‘to hear’

Proto-Turkic. **e̥ʎit* (Sevortyan et al. 1974–: vol. 1: 318; Dybo 2013: 327), attested as a basic term in Chuvash and in the majority of Nuclear Turkic subgroups. Can be analyzed as **e̥ʎi-t* with the common suffix **-t* whose main function is causativity.

Proto-Mongolic. **sono-s-* (Nugteren 2011: 500; Gruntov & Mazo 2015: 229), attested as a basic term in all three subgroups. Final **-s* can be detached as an old suffix, if Mongolic **soni-n* ‘news (< *‘heard’); new, newsworthy, interesting’ (Nugteren 2011: 501) is related.

Proto-Tungusic. **doːldiː-* (Tsintsius 1975: 214–215; Kazama 2003: 80; Starostin, Dybo & Mudrak 2003: 1384), attested as a basic term in all four subgroups.

Middle Korean. *tìr- ~ tìt-* < Proto-Korean **tìd-* (Starostin, Dybo & Mudrak 2003: 1384).

Proto-Japonic. **kí-k-* (Starostin, Dybo & Mudrak 2003: 847, Vovin 1999: 88). Attested in Japanese and Ryukyuan. Final *-k-* is detachable as a common verbal suffix.

Etymological notes. Tungusic **doːldiː-*, Korean **tìd-* < Proto-Altaic **tùːʎdi* (Starostin, Dybo & Mudrak 2003: 1384). The only instance of the hypothetical cluster **ʎd*, but the Tungusic-Korean connection seems likely in any case. Robbeets does not consider this etymology.

40. ‘heart’

Proto-Turkic. **yürek* (Sevortyan et al. 1974–: vol. 4: 270; Tenishev 2001: 276; Dybo 2013: 332), retained in all subgroups except for Chuvash, where it was replaced with a deverbative from **diːri* ‘to live’.

Proto-Mongolic. **ǯirüke-n* (Nugteren 2011: 394; Gruntov & Mazo 2015: 230), attested as a basic term in all three subgroups. Suspiciously close to Proto-Turkic **yürek* ‘heart’, thus might be a Pre-Proto-Turkic loan into Proto-Mongolic before the assumed Proto-Turkic shift **ǯ* > Proto-Turkic **y*.

Proto-Tungusic. **miawan* (Tsintsius 1975: 533–534; Starostin, Dybo & Mudrak 2003: 928), attested as a basic term in all four subgroups. Manchu *ɲaman* ‘heart’ shows the regular shift **miV > ɲV* (Doerfer 1995:

256) and the occasional assimilation *w > m. In Starostin, Dybo & Mudrak 2003: 928, the Proto-Tungusic form is reconstructed as *miaɲam* in order to account *miawan* (Evenic, Nanaic, Udiheic) and *ɲaman* (Manchu-Jurchen) and further compare it with Korean *mʌɲʌm* 'heart'.

Middle Korean. *mʌ̀zʌ̀m* possibly < Proto-Korean *mʌɲʌm* (Starostin, Dybo & Mudrak 2003: 928).

Proto-Japonic. **kə̀kə̀rə* (Starostin, Dybo & Mudrak 2003: 713), **kòkòró* (Vovin 1999: 88). Attested in Japanese and Ryukyuan.

Etymological notes. Turkic **yürek*, Mongolic **ǯirüke-n* < Proto-Altaic **ǯùrVkʰe* (Starostin, Dybo & Mudrak 2003: 1555). Correspondences seem regular. An early borrowing from Proto-Turkic into Proto-Mongolian or *vice versa* can be suspected because of full coincidence of the three-consonant skeletons (from a systemic point of view, Turkic **y* is a voiced counterpart of Proto-Turkic *č*), but there is no positive evidence for a loan scenario. In Starostin, Dybo & Mudrak 2003: 928, Tungusic **miawan* is compared with Proto-Korean **mʌɲʌm* < Proto-Altaic **mi̯óɲù*. Since we now reconstruct the Proto-Tungusic form as **miawan*, the comparison seems less tenable. Robbeets does not consider these etymologies.

41. 'horn'

Proto-Turkic. **buyŋurʸ* (Sevortyan et al. 1974–: vol. 2: 243; Tenishev 2001: 148; Dybo 2013: 335), retained in all subgroups.

Proto-Mongolic. **eber* (Nugteren 2011: 322; Gruntov & Mazo 2015: 230), attested as a basic term in all three subgroups.

Proto-Tungusic. **xüye* (Tsintsius 1975: 298–299; Starostin, Dybo & Mudrak 2003: 815), attested as a basic term in all four subgroups (in Manchu-Jurchen, the suffixed stem **xüye-kte* occurs).

Middle Korean. *spɨ́r*, possibly from Proto-Korean prefixed **s=pɨr* (Starostin, Dybo & Mudrak 2003: 607).

Proto-Japonic. **tùnuá* (Tower of Babel project), **tùnwò* (Vovin 1999: 88). Attested in Japanese and Ryukyuan. The word is sometimes suspected to be of Austronesian origin (Starostin 1991: 114), but is nevertheless perfectly reconstructible for the Proto-Japonic level and may be admitted into formal comparison.

Etymological notes. We follow Starostin, Dybo & Mudrak 2003: 607 in analyzing *spɨ́r* as **s=pɨr* with a petrified prefix **s-*, which allows to compare the root *pɨr* with Mongolic **eber* < Proto-Altaic **i̯opʰérV*. The problem of the origin of Korean initial clusters **sp-, *st-, *sk-* is beyond the scope of the present study, but in our dataset, cf. Middle Korean *s(-)pyə̀* 'bone', *s(-)kòrí* 'tail'.

42. 'I'

Proto-Turkic. nom. **bi*, obl. **bẹ-n-* (Sevortyan et al. 1974–: vol. 2: 243; Dybo 2013: 335), this paradigm was retained in Bulghar, having been simplified in favor of the oblique stem **bẹn* in Nuclear Turkic.

Proto-Mongolic. **bi*, gen. **mi-n-* (< **bi-n*), obl. **na-ma-* (Nugteren 2011: 281; Gruntov & Mazo 2015: 230; Poppe 1955: 209), attested in all three subgroups.

Proto-Tungusic. **bi*, obl. **mi-n-* (< **bi-n-*) (Tsintsius 1975: 79; Starostin, Dybo & Mudrak 2003: 341), attested as a basic term in all four subgroups.

Middle Korean. *nà* (Starostin, Dybo & Mudrak 2003: 1024), no suppletion.

Proto-Japonic. **bà-* (Starostin, Dybo & Mudrak 2003: 341), **bàn[u]* (Vovin 1999: 88), no synchronic suppletion. This is the only 1st sg. p. morpheme present in the absolute majority of both the historically attested and modern forms of Japanese and Ryukyuan; **bà-* is clearly the root, to which various suffixal extensions are attached (e.g. *wa-re, wa-ta-* in Japanese, **ba-nu* in Ryukyuan etc.). However, as a synonymous form it would be advisable to also include **a* (Starostin, Dybo & Mudrak 2003: 1024), well attested in Old Japanese and also seen in some Ryukyuan dialects; some particularities of the usage of this pronominal stem in Old Japanese hint at a possible oblique or possessive stem origin for it.

Etymological notes. Turkic **bi*, Mongolic **bi*, Tungusic **bi*, Japonic **bà-* < Proto-Altaic direct stem **bì* (Starostin, Dybo & Mudrak 2003: 341). Mongolic **na-ma-*, Korean *nà*, Japonic **a* < Proto-Altaic oblique stem **ŋa* (Starostin, Dybo & Mudrak 2003: 1024). Due to vocalic features, Robbeets (Robbeets 2005: 380; Robbeets & Bouckaert 2018) compares Japonic **wa-* 'I' (in her reconstruction) with the Proto-Mongolic 1st person plural exclusive pronoun **ba-n-*, keeping Turkic **bi*, Mongolic **bi*, Tungusic **bi* as a separate etymological entry.

43. 'to kill'

Proto-Turkic. **öl-ɨr ~ *öl-dɨr* (Sevortyan et al. 1974–: vol. 1: 525; Dybo 2013: 339), a regular causative from **öl* 'to die' (q.v.).

Proto-Mongolic. **ala-* (Nugteren 2011: 267; Gruntov & Mazo 2015: 230), attested as a basic term in all three subgroups.

Proto-Tungusic. **waː-* (Tsintsius 1975: 127–129; Kazama 2003: 95; Starostin, Dybo & Mudrak 2003: 512), attested as a basic term in all four subgroups. A unique occurence of the Proto-Tungusic phoneme **w* (since all languages have initial *w-* in this verb, it is hard to reconstruct something else here).

Middle Korean. *čùk-í-* (Starostin, Dybo & Mudrak 2003: 450), causative from *čùk-* 'to die'.

Proto-Japonic. **kə̀rə-s-* (Starostin, Dybo & Mudrak 2003: 671), **kórós-* (Vovin 1999: 88). Attested in Japanese and Ryukyuan. Final *-s-* is clearly detachable as a causative suffix.

44. 'knee'

Proto-Turkic. **diyr^{y}* (Sevortyan et al. 1974–: vol. 3: 336; Tenishev 2001: 284; Dybo 2013: 341), attested in all subgroups except for Yakut.

Proto-Mongolic. **ebü-düg* (Nugteren 2011: 323; Gruntov & Mazo 2015: 230), used as a basic term in Northern and Southern, not attested in Dagur. Derived from **eb-ke-* ‘to bend’. Distinct from **toyig* ‘knee cap’ (Starostin, Dybo & Mudrak 2003: 1460) (Northern only). Dagur **tolčig* ~ **twalčig* ‘knee’ lacks convincing morphological analysis and etymology (Gruntov & Mazo 2015: 231).

Proto-Tungusic. There are two candidates. (1) **peɲ-ŋen* (Tsintsius 1977: 366; Starostin, Dybo & Mudrak 2003: 1131), a basic term in Evenic, Nanaic and Udiheic, without Manchu-Jurchen cognates. (2) **topVg-* (Tsintsius 1977: 189; Starostin, Dybo & Mudrak 2003: 1459), a basic term in Manchu, without transparent cognates in other subgroups. If Manchu-Jurchen is indeed the first outlier, these candidates are equally probable. We prefer to treat them as technical synonyms.

Middle Korean. *mùrɨ̀p*^{h} ~ *mùrùp*^{h} ~ *mùròp*^{h} (Starostin, Dybo & Mudrak 2003: 938).

Proto-Japonic. **pínsá* (Starostin, Dybo & Mudrak 2003: 345, Vovin 1999: 88). Attested in Japanese. The most common Ryukyuan equivalent for ʽknee’ is a form reconstructed in Starostin, Dybo & Mudrak 2003: 1459 as Proto-Japonic **tu(m)pu-*, but the actual reflexes rather suggest a trisyllabic **tubusi(N)* in Proto-Ryukyuan (**tubusin* in Vovin 1999: 88) which may be analyzed as a compound with the second element = Proto-Japonic **pusi* ʽknot, joint’ (first element is unclear). Given that Proto-Ryukyuan **pisa* ʽfoot, leg, knee’ (Thorpe 1983: 299), related to Proto-Japanese **pínsá*, also exists, although its precise semantics in Proto-Ryukyuan is hard to reconstruct, Ryukyuan **tubusi(N)* is most likely an innovation and has to be excluded from comparison.

45. ‘to know’

Proto-Turkic. **bil* (Sevortyan et al. 1974–: vol. 2: 137; Dybo 2013: 348), retained in all subgroups. The verb has a general meaning ‘to know’ in Chuvash and some Nuclear languages, but in many Nuclear languages it shows a narrow semantics ‘to know that…’. If **bil* is to be reconstructed as ‘to know (that…)’, not ‘to know (in general)’, a possible candidate for ‘to know (smth., smb.)’ is **ta̝nu* (Dybo 2013: 350), meaning ‘to know (smth., smb.)’ in Nuclear Turkic and as a nominal derivative ‘witness’ in Chuvash.

Proto-Mongolic. **mede-* (Nugteren 2011: 442; Gruntov & Mazo 2015: 231), attested as a basic term in all three subgroups. Meaning ‘to know (in general)’.

Proto-Tungusic. **sa:-* (Tsintsius 1977: 49–51; Kazama 2003: 111; Starostin, Dybo & Mudrak 2003: 1219), attested as a basic term in all four subgroups.

Middle Korean. *ă:r-* (Starostin, Dybo & Mudrak 2003: 293).

Proto-Japonic. **sír-* (Starostin, Dybo & Mudrak 2003: 1219, Vovin 1999: 88).

Etymological notes. Tungusic **sa:-*, Japonic **sír-* < Proto-Altaic **sá:r*^{y}*i* (Starostin, Dybo & Mudrak 2003: 1219; Robbeets 2005: 303). Altaic **-r-* is lost in Tungusic after a long vowel, i.e., in the roots of the shape *CV:r-* (e.g., ‘to give’, ‘water’, ‘yellow’ q.v.).

46. ‘leaf’

Proto-Turkic. **yapur-gak* (Sevortyan et al. 1974–: vol. 4: 130; Tenishev 2001: 111; Dybo 2013: 351), final **-gak* is a common nominal suffix. Attested as a basic term for ‘leaf (of tree)’ in all Nuclear Turkic subgroups except for Tofa-Tuvinian, South Siberian and Altay (retained in Altay as ‘leaf of grass’). In Chuvash, a new formation from **yaːʎ* ‘fresh, moist, unripe’ is used (Dybo 2013: 353). In Tofa-Tuvinian, South Siberian and Altay, superseded with **bür* ‘seed; bud’ (Tenishev 2001: 114; Dybo 2013: 352).

Proto-Mongolic. **labči-n* ~ **nabči-n* (Nugteren 2011: 450; Gruntov & Mazo 2015: 231), attested as a basic term in all three subgroups. Apparently the reconstruction with initial **l-* is more justified than the traditional one with **n-*, but we accept both forms as equiprobable items for formal analysis.

Proto-Tungusic. **xabda-nsa* (Tsintsius 1975: 5; Kazama 2003: 37; Starostin, Dybo & Mudrak 2003: 764), attested as a basic term in all four subgroups. The root itself is retained in Manchu within the verb *abda-la-* ~ *abta-la-* ‘to break off (branches), to prune’, but it remains unclear whether **xabda-* was a verbal root or a nominal one in Proto-Tungusic.

Middle Korean. *níp*^{h} (Starostin, Dybo & Mudrak 2003: 874).

Proto-Japonic. **pá* (Starostin, Dybo & Mudrak 2003: 1111, Vovin 1999: 88). Attested in Japanese and Ryukyuan.

Etymological notes. Turkic **yapur-gak*, Mongolic **labči-n* ~ **nabči-n*, Korean *níp*^{h} < Proto-Altaic **liàp*^{h}*à* (Starostin, Dybo & Mudrak 2003: 874). Correspondences seem regular, assuming fossilized *r-* and *č*-suffixes in Turkic and Mongolic. Robbeets does not consider this etymology.

47. ‘to lie’

Proto-Turkic. **ya̞t* (Sevortyan et al. 1974–: vol. 4: 156; Dybo 2013: 354), attested as a basic term in all Nuclear Turkic subgroups. Chuvash *vɨrt* ‘to lie’ probably goes back to a suffixed stem **or-t* from the bound root **or* ‘place’ or ‘to be in place’ (Dybo 2013: 356), although morphological details are not entirely clear since the verbal suffix **-t* normally forms causatives.

Proto-Mongolic. **kebte-* (Nugteren 2011: 450; Gruntov & Mazo 2015: 231), attested as a basic term in all three subgroups.

Proto-Tungusic. Not reconstructible, see notes on ‘to sleep’.

Middle Korean. *nŭːβ-* < Proto-Korean **nŭːb-* (Starostin, Dybo & Mudrak 2003: 964).

Proto-Japonic. **na-* (Starostin, Dybo & Mudrak 2003: 964), **ná-* (Vovin 1999: 88). Attested in Japanese and Ryukyuan.

Etymological notes. Starostin, Dybo & Mudrak 2003: 964 treat Korean **nŭːb-* and Japonic **na-* as cognates < Proto-Altaic **néː*, assuming a fossilized labial suffix in Korean. This is not excluded, but it seems more prudent to keep these forms apart. Note that this suffix has probably nothing to do with the productive

suffix *-*b*- which forms verb-like adjectives (Lee & Ramsey 2011: 180–181). Robbeets (Robbeets 2005: 386; Robbeets & Bouckaert 2018) rejects the Korean-Japonic etymology.

48. ‘liver’

Proto-Turkic. **biagɨr* (Sevortyan et al. 1974–: vol. 2: 17; Tenishev 2001: 278; Dybo 2013: 357), retained in all subgroups.

Proto-Mongolic. **heli-gen* (Nugteren 2011: 353; Gruntov & Mazo 2015: 232), attested as a basic term in all three subgroups.

Proto-Tungusic. **pa:ki-n* (Tsintsius 1977: 310; Starostin, Dybo & Mudrak 2003: 1092), attested as a basic term in all four subgroups.

Middle Korean. *kàn*, borrowed from Middle Chinese 肝 *kân*.

Proto-Japonic. **kìmuà* (Starostin, Dybo & Mudrak 2003: 775), **kímwò* (Vovin 1999: 88). Attested in Japanese and Ryukyuan.

Etymological notes. Turkic **biagɨr*, Mongolic **pa:ki-n* < Proto-Altaic **pià:ki* (Starostin, Dybo & Mudrak 2003: 1092). Correspondences seem regular, assuming a fossilized *r*-suffix in Turkic. Robbeets does not consider this etymology.

49. ‘long’

Proto-Turkic. **urʸɨ-m* (Sevortyan et al. 1974–: vol. 1: 570; Dybo 2013: 359), derived from the verb **urʸ(a)* ‘to stretch (intr.), be long’. Retained in all subgroups.

Proto-Mongolic. **urtu* (Nugteren 2011: 534; Gruntov & Mazo 2015: 232), attested as a basic term in all three subgroups.

Proto-Tungusic. **ŋo:li-mi-* (Tsintsius 1975: 664–665; Kazama 2003: 117; Starostin, Dybo & Mudrak 2003: 1035), attested as a basic term in all four subgroups.

Middle Korean. *kǐ:r-* (Starostin, Dybo & Mudrak 2003: 695).

Proto-Japonic. **nànkà-* (Starostin, Dybo & Mudrak 2003: 1035, Vovin 1999: 88). Attested in Japanese and Ryukyuan.

Etymological notes. Turkic **urʸɨ-m*, Mongolic **urtu* < Proto-Altaic **iurʸo* (Starostin, Dybo & Mudrak 2003: 623; Robbeets & Bouckaert 2018), correspondences seem regular, assuming a fossilized *t*-suffix in Mongolic. Additionally Starostin, Dybo & Mudrak 2003: 1035 treat Tungusic **ŋo:li-mi-* and Japonic **nànkà-* as cognates < Proto-Altaic **ŋò:la*, supposing a fossilized velar suffix in Japonic. However, the assumed development *lk* > *nk* in Proto-Japonic is not confirmed by additional data and we prefer to keep

Tungusic **ŋo:li-mi-* and Japonic **nànkà-* apart. Robbeets (2005: 318, 350) accepts the Tungusic-Japonic comparison, but later (Robbeets & Bouckaert 2018) rejects it.

50. 'louse'

Proto-Turkic. **biyt* (Sevortyan et al. 1974–: vol. 2: 151; Tenishev 2001: 182; Dybo 2013: 361), retained in all subgroups.

Proto-Mongolic. **böhe-sün* (Nugteren 2011: 287; Gruntov & Mazo 2015: 232), attested as a basic term in all three subgroups.

Proto-Tungusic. There are two candidates in complementary distribution. (1) **kumke* (Tsintsius 1975: 430; Starostin, Dybo & Mudrak 2003: 738), a basic term in Evenic and Udiheic. (2) **ti-kte* (Tsintsius 1977: 179; Starostin, Dybo & Mudrak 2003: 1425), a basic term in Nanaic and Manchu. Cf. the cognate verbs 'to search for lice in one's hair': **ti:-le-* (Evenic) and **ti:-na-* (Oroch) (Tsintsius 1977: 181). We take **kumke* and **ti-kte* as technical synonyms. Note that if Udiheic indeed belong to the Northern subgroup and Manchu-Jurchen is the first outlier, **ti-kte* has the advantage, whereas **kumke* would be a Northern innovation.

Middle Korean. *ní* (Starostin, Dybo & Mudrak 2003: 966).

Proto-Japonic. **sìrámí* (Starostin, Dybo & Mudrak 2003: 1263, Vovin 1999: 88). Attested in Japanese and Ryukyuan.

51. 'man'

Proto-Turkic. **e:r* (Sevortyan et al. 1974–: vol. 1: 297; Tenishev 2001: 303; Dybo 2013: 367), retained in all subgroups.

Proto-Mongolic. **ere* (Nugteren 2011: 331; Gruntov & Mazo 2015: 232), the root is attested in all three subgroups. Apparently to be reconstructed with polysemy 'man / male (of animals)'. In some modern languages, the bare root **ere* is retained with the meaning 'male', whereas the meaning 'man' is expressed with the help of suffixed stems, e.g., Khamnigan *ore-gtei* 'man'. The reverse strategy is observed in Dagur: *ər* 'man', *ər-gun* 'husband; male (of animals)'. Cf. the same situation with **eme* 'woman; female'.

Proto-Tungusic. Two technical synonyms: **kaka* and **xüse*, see notes on 'person'.

Middle Korean. *nàmčìn*, borrowed from Middle Chinese 男人 *nʌm-ɲin*. The original term was probably *àtʌ́r*, retained in Late Middle Korean with the meaning 'son'.

Proto-Japonic. **bə̀* (Starostin, Dybo & Mudrak 2003: 335) **bò* (Vovin 1999: 88). The simple root means 'male' in general; words for 'man' are formed from it in different ways depending on the dialect (usually **bə̀-nə-kua* or **bə̀-tu-kua*, where **kua* = 'child').

Etymological notes. Turkic **eːr*, Mongolic **ere* < Proto-Altaic **ǻːri* (Starostin, Dybo & Mudrak 2003: 312). Correspondences seem regular. The difference in root structure makes the scenario of borrowing from Turkic into Mongolic unlikely. Robbeets does not consider this etymology.

52. 'many'

Proto-Turkic. **köp* (Sevortyan et al. 1974–: vol. 5: 107; Dybo 2013: 370), attested in all subgroups except for Chuvash (no inherited term?) and Yakut; in Ancient Turkic sources it means 'much, abundant', more rarely 'many' (Clauson 1972: 686). The second candidate is **öːk-üš* 'many' which is widespread in Ancient Turkic sources (Clauson 1972: 118) as well as in the Yakut subgroup. An unclear criss-crossed configuration. The stem **öːk-üš* is a regular deverbative from **öːk* 'to heap up, accumulate', thus the primary stem **köp* should have the advantage over **öːk-üš*; on the other hand, due to the particular importance of Ancient Turkic as a data source we treat **köp* and **öːk-üš* as synonyms (note that the choice is irrelevant to further phylogenetic purposes).

Proto-Mongolic. **olan* (Nugteren 2011: 467; Gruntov & Mazo 2015: 233), attested as a basic term in all three subgroups.

Proto-Tungusic. **egdi* (Tsintsius 1977: 359–360; Kazama 2003: 122; Starostin, Dybo & Mudrak 2003: 495), attested as a basic term in Evenic (Negidal), Nanaic and Udiheic. Jurchen *amba-la* 'many' is a secondary derivative from **amba* 'big' (q.v.); Manchu *labdu* 'many; much' < **labdu* (Tsintsius 1975: 485; Kazama 2003: 122; Starostin, Dybo & Mudrak 2003: 859) should also be an innovation.

Middle Korean. *há-* and *mǎːn-hʌ́-* (Starostin, Dybo & Mudrak 2003: 901). Although it has been suggested (Robbeets 2005: 312) that the source of the second form might be the Chinese numeral 萬 *wàn* (Middle Chinese *mwə̀n*) '10.000', such a scenario is not highly likely since the Chinese word has always meant 'many' (or 'all') only in certain idiomatic collocations (e.g. 萬物 *wàn wù* '10.000 things' = 'all that exists'), and it is not clear how it could have been assimilated into basic Korean usage in the neutral and unbound meaning 'many'. More likely, this is just an example of chance sound coincidence.

Etymological notes. Korean *mǎːn-*, Japonic **mana-i-* < Proto-Altaic **mana* (Starostin, Dybo & Mudrak 2003: 901). Correspondences seem regular. Robbeets (2005: 312; Robbeets & Bouckaert 2018) rejects this etymology, treating Korean *mǎːn-* as a Chinese loanword ("a Sino-Korean adjectival noun *¨MAN* 'myriad'"), for which see notes on the Korean list.

53. 'meat'

Proto-Turkic. **et* (Sevortyan et al. 1974–: vol. 1: 311; Tenishev 2001: 455; Dybo 2013: 374), attested in all subgroups.

Proto-Mongolic. **mika-n* (Nugteren 2011: 443; Gruntov & Mazo 2015: 233), attested as a basic term in all three subgroups.

Proto-Tungusic. **ul-* (Tsintsius 1977: 262; Starostin, Dybo & Mudrak 2003: 1495), attested as a basic term in non-Manchu-Jurchen subgroups with various desemanticized suffixes: **ul(e)-se* in Evenic and Nanaic, **ul(e)-kse* in Nanaic, **ul(e)-kte* in Udiheic. The second candidate is **ɲali-* (Tsintsius 1975: 340, 630; Starostin, Dybo & Mudrak 2003: 985), meaning 'meat' in Manchu-Jurchen and 'raw' in other subgroups. Since the direction of the semantic shift 'raw' > 'meat' seems much more natural than *vice versa*, **ɲali-* can be ruled out.

Middle Korean. *kòkí* (Starostin, Dybo & Mudrak 2003: 735).

Proto-Japonic. **sìsì* (Starostin, Dybo & Mudrak 2003: 1332, Vovin 1999: 88). Attested in Japanese and Ryukyuan. Replaced by the Chinese borrowing *niku* in most modern Japanese dialects.

54. 'moon'

Proto-Turkic. **aɲ* (Sevortyan et al. 1974–: vol. 1: 98; Tenishev 2001: 55; Dybo 2013: 376), retained in all subgroups. Attested languages mostly point to **-y*; nasal **-ɲ* is restored on the basis of Tofa-Tuvinian nasalization and an Ancient Turkic (Cuman) form recorded in Old Russian sources as *aːn*^{y} ~ *aːn*. Proto-Chuvash **oузк* contains a diminutive suffix.

Proto-Mongolic. **sara-n* (Nugteren 2011: 483; Gruntov & Mazo 2015: 233), attested as a basic term in Northern and Southern and as 'month' in Dagur.

Proto-Tungusic. **biaga* (Tsintsius 1975: 78–79; Kazama 2003: 47), attested as a basic term in all four subgroups.

Middle Korean. *tʌ́r* (Starostin, Dybo & Mudrak 2003: 1435).

Proto-Japonic. **tùkùi* (Starostin, Dybo & Mudrak 2003: 1435), **tùkú-* (Vovin 1999: 89). Attested in Japanese and Ryukyuan.

Etymological notes. Korean *tʌ́r*, Japonic **tùkù-i* < Proto-Altaic **t*^{h}*i̯òlgu* (Starostin, Dybo & Mudrak 2003: 1435; Robbeets 2005: 336), correspondences seem regular.

55. 'mountain'

Proto-Turkic. **daːg* (Sevortyan et al. 1974–: vol. 3: 117; Tenishev 2001: 94, 111; Dybo 2013: 378), attested in all subgroups except for Yakut.

Proto-Mongolic. **ahula* (Nugteren 2011: 275; Gruntov & Mazo 2015: 233), attested as a basic term in all three subgroups. Distinct from **kada* 'cliff, rock' (Nugteren 2011: 398).

Proto-Tungusic. **xureː* (Tsintsius 1977: 289; Kazama 2003: 42; Starostin, Dybo & Mudrak 2003: 843), meaning 'mountain' in non-Manchu-Jurchen. Its diminutive derivative **xureː-ke* means 'hill' in Nanai and 'stone; rock, cliff' in Manchu-Jurchen. Cf. the stem **ala-n* (Tsintsius 1975: 27–28; Kazama 2003: 42; Starostin, Dybo & Mudrak 2003: 292), meaning 'mountain' in Manchu-Jurchen and 'mountain pass; to

cross (a mountain)’ in Evenic. The plain stem **ala* is attested with the meanings ‘small mountain, hill; mountain pass; to cross (a mountain)’.

Middle Korean. *mǒ:yh* (Starostin, Dybo & Mudrak 2003: 956).

Proto-Japonic. **dàmà* (**yàmà*) (Starostin, Dybo & Mudrak 2003: 464, Vovin 1999: 89). Attested in Japanese and Ryukyuan. Vovin (1999: 89) adds **tàka-Ci* as a synonym, since this word may indeed mean ‘mountain’ in Japanese (not Ryukyuan), but its primary meaning is still clearly adjectival (‘high’), so we do not formally take it into consideration.

56. ‘mouth’

Proto-Turkic. **agɨr*^{y} (Sevortyan et al. 1974–: vol. 1: 81; Tenishev 2001: 224; Dybo 2013: 381), attested as ‘mouth’ in all subgroups except for Chuvash (where it means ‘mouth of river’) and probably Yakut-Dolgan (where it means ‘lip(s); muzzle of gun’). Since the shifts ‘mouth’ > ‘mouth of river’ or ‘mouth’ > ‘muzzle of gun’ are very common typologically and the shift ‘mouth’ > ‘lip’ is also attested cross-linguistically, **agɨr*^{y} is the main candidate for Proto-Turkic ‘mouth’. It is likely that historically this stem is to be analyzed as **ag-ɨr*^{y} with the dual suffix which should imply the Pre-Proto-Turkic meaning ‘lip’ for **ag-*.

Proto-Mongolic. **ama-n* (Nugteren 2011: 269; Gruntov & Mazo 2015: 234), attested as a basic term in all three subgroups.

Proto-Tungusic. **amŋa* (Tsintsius 1975: 38–39; Kazama 2003: 18; Starostin, Dybo & Mudrak 2003: 296), attested as a basic term in all four subgroups. The metathesized form *aŋma* ‘mouth’ attested in Nanaic and Udiheic (sometimes as a doublet of *amŋa*) can be plausibly explained by contamination with **aŋa* ‘hole’ (thus S. Starostin et al.). The form *aŋma* which spread across the central area is not a sufficient reason for reconstructing Proto-Tungusic **aŋma* (*pace* Doerfer 1995: 256)

Middle Korean. *íp* (Starostin, Dybo & Mudrak 2003: 589).

Proto-Japonic. **kútí* (Starostin, Dybo & Mudrak 2003: 682), **kútú-Ci* (Vovin 1999: 89). Attested in Japanese and Ryukyuan.

Etymological notes. Mongolic **ama-n*, Tungusic **amŋa* < Proto-Altaic **ámo* (Starostin, Dybo & Mudrak 2003: 296; Robbeets 2005: 478–479; Robbeets & Bouckaert 2018). Correspondences seem regular, assuming a fossilized *ŋ*- or *g*-suffix in Tungusic.

57. ‘name’

Proto-Turkic. **iat* (Sevortyan et al. 1974–: vol. 1: 198; Dybo 2013: 390), attested as ‘name’ in all subgroups.

Proto-Mongolic. **nere* (Nugteren 2011: 455; Gruntov & Mazo 2015: 234), attested as a basic term in all three subgroups.

Proto-Tungusic. **gerbü:* (Tsintsius 1975: 180–181; Kazama 2003: 68; Starostin, Dybo & Mudrak 2003: 541), attested as a basic term in all four subgroups.

Middle Korean. *ìrh-úm* (Starostin, Dybo & Mudrak 2003: 973).

Proto-Japonic. **ná* (Starostin, Dybo & Mudrak 2003: 888, Vovin 1999: 89). Attested in Japanese and Ryukyuan (Hateruma nàN allows Starostin et al. to suggest *náŋ as a possible reconstruction, but the evidence for an archaic root-final nasal is too flimsy).

Etymological notes. Starostin, Dybo & Mudrak 2003: 973 treat Korean *ìrh-úm* 'name' as a derivative from *nìrʔ-* 'to say', further to Mongolic **nere* 'name' < Proto-Altaic **nére*. Internal Korean evidence suggests, however, that *ìrh-úm* and *nìrʔ-* are unrelated, thus the comparison between Korean *ìrh-úm* 'name' and Mongolic **nere* 'name' is to be ruled out (in any case the Korean name is a deverbative and this match would be a root cognacy at best). Robbeets does not consider the Mongolic-Korean etymology.

58. 'neck'

Proto-Turkic. **bo:yɨn* (Sevortyan et al. 1974–: vol. 2: 180; Tenishev 2001: 233; Dybo 2013: 392), attested in all subgroups.

Proto-Mongolic. **küǯü-hün* (Nugteren 2011: 435; Gruntov & Mazo 2015: 234), attested as a basic term in all three subgroups.

Proto-Tungusic. **moŋgo-n* (Tsintsius 1975: 546; Starostin, Dybo & Mudrak 2003: 939), attested as a basic term in non-Manchu-Jurchen subgroups. In Manchu, **moŋgo-n* means 'front part of neck; throat', but Manchu retains several suffixed derivatives, such as 'bottle with a narrow mouth and long neck' or 'ornamental neckband worn by women', which should prove that the original Manchu meaning of **moŋgo-* was 'neck'. Manchu-Jurchen *meyfen* 'neck' (Tsintsius 1975: 538) is of unclear origin. It is proposed in Starostin, Dybo & Mudrak 2003: 939 to analyze **moŋgo-n* as **moɲ-gan* and Manchu-Jurchen *meyfen* as < **moɲ-pen* with the virtual root **moɲ-* 'neck' extended with rare if not unique suffixes.

Middle Korean. *mòk* (Starostin, Dybo & Mudrak 2003: 939).

Proto-Japonic. *kúmpí* (Starostin, Dybo & Mudrak 2003: 718), *kúnpí* (Vovin 1999: 89). Attested in Japanese and Ryukyuan.

Etymological notes. Turkic **bo:yɨn*, Tungusic **moŋgo-n*, Korean *mòk* < Proto-Altaic **mó:ɲo* (Starostin, Dybo & Mudrak 2003: 939). One should assume fossilized velar suffixes in Tungusic and Korean. The Turkic and Tungusic protoforms are likely related, but it is not entirely clear how the Altaic medial nasal element must be reconstructed. Robbeets does not consider this etymology.

59. 'new'

Proto-Turkic. **yaŋɨ* ~ **yeŋi* (Sevortyan et al. 1974–: vol. 4: 124; Tenishev 2001: 85; Dybo 2013: 404), attested in all subgroups.

Proto-Mongolic. **sine* ~ **sini* (Nugteren 2011: 496; Gruntov & Mazo 2015: 234), attested as a basic term in all three subgroups.

Proto-Tungusic. **xirke* (Tsintsius 1975: 328; Kazama 2003: 121), attested as a basic term in all four subgroups. The plain root **xirke* is used in Manchu-Jurchen and the suffixed stem **xirke-ken* in other subgroups. In Starostin, Dybo & Mudrak 2003: 808, it is unconvincingly derived from **xirki* 'deer (in the summer/autumn period)'.

Middle Korean. *sáy* (Starostin, Dybo & Mudrak 2003: 1510).

Proto-Japonic. **nípí-* (Starostin, Dybo & Mudrak 2003: 964). Attested in Japanese and Ryukyuan. For some reason, not listed in Vovin 1999: 89, where **àrà-ta-* (Starostin, Dybo & Mudrak 2003: 315) is instead offered as the Proto-Japanese equivalent for ʽnew'. However, this second stem is most likely an innovation, given its limited distribution (not reconstructible for Proto-Ryukyuan), complex morphological structure, and possible connections to the verb **àrà-pa-* ʽto appear'.

Etymological notes. Turkic **yaŋɨ*, Mongolic **sine* < Proto-Altaic **zèyṇa* (Starostin, Dybo & Mudrak 2003: 1510). Correspondences seem regular, assuming a fossilized *k/g*-suffix in Turkic. Robbeets does not consider this etymology.

60. 'night'

Proto-Turkic. The most interesting candidate is **yɨr-* (Dybo 2013: 408), which means 'night' in some Chuvash dialects and 'north' in Ancient Turkic (Clauson 1972: 954). Since the meaning shift 'night' > 'north' is common cross-linguistically, but not *vice versa*, **yɨr-* looks like an archaism in Chuvash, almost totally lost in the Nuclear Turkic branch. The second candidate is **tün* (Sevortyan et al. 1974–: vol. 3: 315; Tenishev 2001: 81; Dybo 2013: 406), attested as a basic term for 'night' in almost all Nuclear Turkic subgroups (the main exception is Oghuz), but absent in Chuvash. Finally there exists the stem **gẹːč-e* (Sevortyan et al. 1974–: vol. 3: 40; Tenishev 2001: 82; Dybo 2013: 407), attested as a basic term for 'night' in some Chuvash dialects (also 'evening'), Oghuz, Karluk (also 'evening'), Kipchak. In other lects it means 'evening'. In Ancient Turkic sources, **gẹːč-e* is attested as both 'night' and 'evening' (Clauson 1972: 694; Tenishev 2001: 82). The stem **gẹːč-e* is a regular derivative from Proto-Turkic **gẹːč* 'late, belated' which makes this candidate for the status of Proto-Turkic or Proto-Nuclear Turkic 'night' weaker than the primary stem **tün*. The second argument against **gẹːč-e* is that **gẹːč-e* seems to be the main candidate for the meaning 'evening' in Proto-Turkic (the stem **iŋir* is rather to be reconstructed as 'dusk, twilight', not 'evening' pace Tenishev 2001: 81). Thus we reconstruct the opposition **tün* 'night' and **gẹːč-e* 'evening' (the latter tends to shift towards the meaning 'night' due to interdialectal influence) at least for Nuclear Turkic. As for the Proto-Turkic slot, we fill it with two forms: **yɨr-* and **tün*.

Proto-Mongolic. **söni* (Nugteren 2011: 504; Gruntov & Mazo 2015: 234), attested as a basic term in all three subgroups.

Proto-Tungusic. **dolbo* (Tsintsius 1975: 213–214; Kazama 2003: 50; Starostin, Dybo & Mudrak 2003: 484), attested as a basic term in all four subgroups.

Middle Korean. *pám* (Starostin, Dybo & Mudrak 2003: 1078).

Proto-Japonic. **duà* (**yuà*) (Starostin, Dybo & Mudrak 2003: 484), **dùCà* (Vovin 1999: 89).

Etymological notes. Starostin, Dybo & Mudrak 2003: 484 treat Tungusic **dolbo* and Japonic **duà* as cognates < Proto-Altaic **dùle*, assuming a *gV*-suffix in Pre-Proto-Japonic which caused the loss of *l* (and a fossilized *bV*-suffix in Tungusic). This is not excluded, but the etymology necessitates too many unprovable assumptions for the moment. Robbeets (2005: 942) reconstructs initial *y*- in the Japonic form and keeps it apart from Tungusic **dolbo*.

61. 'nose'

Proto-Turkic. The main candidate is **bur-un* (Sevortyan et al. 1974–: vol. 2: 269; Tenishev 2001: 214; Dybo 2013: 409), attested as a basic term for 'nose' in the majority of the Nuclear Turkic subgroups (Yakut, South Siberian, Oghuz, Karluk, Kipchak, Altay) as well as in Ancient Turkic sources. It can be analyzed as **bur-un*, an instrumental deverbative from **bur* 'to smell (intr.)'. The second and weaker candidate is Chuvash **səmsa* 'nose' (Dybo 2013: 414) which lacks Nuclear Turkic *comparanda* and most likely represents a Mongolic loan. Virtual Turkic **sumsV* (> Chuvash **səmsa*) and Tungusic **soŋgi* 'nose' (q.v.) are sometimes thought to be cognates < Proto-Altaic **suma* (Starostin, Dybo & Mudrak 2003: 1317), but even assuming a fossilized *g*-suffix in Tungusic, clusters of the shape *-mg-, -mk-* are expected to be retained in Tungusic.

Proto-Mongolic. **kaŋ-bar* (Nugteren 2011: 396; Gruntov & Mazo 2015: 234), attested as a basic term in all three subgroups. Phonological and morphological reconstruction requires comments. The attested forms point to two different shapes of the stem: either **kabar* (Southern) or **kamar* (Northern and Dagur). Such a fluctuation *b* ~ *m* is irregular. Starostin et al. (2003: 806) plausibly propose to explain it as different results of simplification of an uncommon cluster **ŋb*, reconstructing **kaŋ-bar*, where **-bar* is an instrumental affix (Poppe 1955: 201) and **kaŋ-* is a relic bound root 'to smell(?)', cf., e.g., its derivatives in Classical Mongolic *qaŋqul-* 'to emit a strong odor, to smell', *qaŋsa-* 'to speak through the nose', *qaŋsiyar-* 'bridge of the nose' (Lessing 1960: 929–930). The semantic development 'nose' as an instrument of smelling is normal cross-linguistically.

Proto-Tungusic. The best candidate is **ŋiaksa* (Tsintsius 1975: 587; Kazama 2003: 17; Starostin, Dybo & Mudrak 2003: 1030), attested as a basic term in Nanaic (Lower Amur Nanai, Ulch, Orok), Udiheic (Udihe, Oroch) and some peripheral Evenic lects (Solon, Arman Even, plus it means 'bear's nose' in Negidal). In the majority of Evenic lects, it was superseded with **xoŋo-kto* (Tsintsius 1977: 22; Kazama 2003: 17; Starostin, Dybo & Mudrak 2003: 806), a basic term in Evenki ('nose of human, mammal, bird; bow of boat; toe of shoe'), Even ('nose of human, mammal'), Negidal ('nose of human, mammal, bird; bow of boat; toe of shoe') which probably spread via dialect contacts. This is a trivial derivative from **xoŋo*, attested in Evenic with the meaning 'bow of boat', its diminutive **xoŋo-ko* is attested in Nanaic (Ulch, Orok) and Manchu as 'bow of boat' or simply 'end (of an object)'. In Jurchen, 'nose' is expressed with the help of **soŋgi*, whose derivatives *soŋgi-xa, soŋgi-n* mean 'tip of nose' in Manchu (Tsintsius 1977: 61; Starostin, Dybo & Mudrak 2003: 1317), without further etymology. In Manchu, it was superseded with **oporo*

(Tsintsius 1977: 22; Kazama 2003: 17; Starostin, Dybo & Mudrak 2003: 607) of unclear origin, later probably borrowed in Nanai (most dialects) and Kilen as basic terms for ‘nose’. If Manchu-Jurchen is indeed the first outlier, both **ŋiaksa* and **soŋgi* are equally probable candidates, so we take them as synonyms.

Middle Korean. *kóh* (Starostin, Dybo & Mudrak 2003: 807).

Proto-Japonic. **páná* (Starostin, Dybo & Mudrak 2003: 1185, Vovin 1999: 89).

Etymological notes. According to Starostin, Dybo & Mudrak 2003: 806, Mongolic **kaŋ-bar* and Korean *kóh* < Proto-Altaic **kʰi̯oŋa*. This etymology faces certain difficulties, both phonological (loss of **ŋ* in Korean) and semantic (the Mongolic stem is likely to be derived from a verb ‘to smell’). Thus, at best, we are dealing with parallel development in Mongolic and Korean. Robbeets does not consider this etymology.

62. ‘not’

Proto-Turkic. **=mV* (Dybo 2013: 417), a verbal suffix of negation of assertion, attested in all subgroups (in Nuclear Turkic it is also used for prohibitive, although in Chuvash negation of assertion *=ma* is opposed to the prohibitive particle *an*).

Proto-Mongolic. As noted by Poppe (1955: 287–289, 290) there are two main exponents of negation of assertion which are well attested in ancient and modern languages and thus can be safely reconstructed for Proto-Mongolic: the negative verb *ese-* ‘not to be’ (Nugteren 2011: 333; Gruntov & Mazo 2015: 235) and the particle **üle* (Nugteren 2011: 541; Gruntov & Mazo 2015: 235). **ese-* may be generally associated with the past tense, whereas **üle* is characteristic of the present tense, but more detailed analysis and reconstruction are needed. The third candidate is the particle **ügei* (Nugteren 2011: 539; Gruntov & Mazo 2015: 235) usually associated with nominal forms (Poppe 1955: 289).

Proto-Tungusic. In non-Manchu-Jurchen subgroups, the basic way to express negation of assertion is the construction with the negative copula **e-* (Tsintsius 1977: 432; Starostin, Dybo & Mudrak 2003: 488) and a participle. The same negative copula **e-* is used for prohibitive. Negation in nominal phrases is expressed with the help of the particles **aːn, *aːn-či* ‘there is no’ (Tsintsius 1975: 41, 60; Starostin, Dybo & Mudrak 2003: 300). Differently in Manchu, where negation of assertion is expressed with the help of the enclitic *aku* attached to participles (Tsintsius 1975: 60) and prohibitive is expressed with the help of the particle *ume*. The origin of Manchu *aku* is not clear, cf. Starostin, Dybo & Mudrak 2003: 300, where *aku* is probably analyzed as **aːn-ku* with the irregular simplification *nk* > *k*. If Manchu-Jurchen is indeed the first outlier, **e-* and **aku* are equiprobable candidates, so we take them as technical synonyms.

Middle Korean. *àní* (Starostin, Dybo & Mudrak 2003: 300).

Proto-Japonic. **nà- ~ *-(a)n- ~ *ìná* (Starostin, Dybo & Mudrak 2003: 300, with notes on separate functions of the different variants), **-an[a]-* (Vovin 1999: 89). Attested in Japanese and Ryukyuan. All known morphological variants of the Japonic negation probably go back to the same root, but it is difficult to reconstruct a single original shape, so it is necessary to take at least the variants **na-* and and **an-* as formal “synonyms”.

Etymological notes. Mongolic **ese-* is traditionally compared with Tungusic **e-* < Altaic **e* (Starostin, Dybo & Mudrak 2003: 488), but the element *-se-* in the Mongolic form remains unexplained, making this monovocalic comparison more dubious. Korean *ànɨ́*, Japonic **(a)n(a)* < Proto-Altaic **à:ni* (Starostin, Dybo & Mudrak 2003: 300). Robbeets (2015: 183–191, 192–202; Robbeets & Bouckaert 2018) accepts both etymologies.

63. ‘one’

Proto-Turkic. **bir* (Sevortyan et al. 1974–: vol. 2: 146; Dybo 2013: 421), attested in all subgroups.

Proto-Mongolic. **nike-n* ~ **nige-n* (Nugteren 2011: 460; Gruntov & Mazo 2015: 235), attested as a basic term in all three subgroups.

Proto-Tungusic. **emu* ~ **umu* (Tsintsius 1977: 270–272; Kazama 2003: 53; Starostin, Dybo & Mudrak 2003: 505), attested as a basic term in all four subgroups.

Middle Korean. *hʌ̀n* (Starostin, Dybo & Mudrak 2003: 1280).

Proto-Japonic. **pitə* (Starostin, Dybo & Mudrak 2003: 407), **pito* (Vovin 1999: 89). Attested in Japanese and Ryukyuan.

Etymological notes. Turkic **bir*, Japonic **pitə* < Proto-Altaic **bi̯uri* (Starostin, Dybo & Mudrak 2003: 364; Robbeets 2005: 407; Robbeets & Bouckaert 2018), correspondences seem regular except for the fact that the distribution between Japonic **r* and **t* as an outcome of Proto-Altaic **r* remains unclear.

64. ‘person’

Proto-Turkic. **kiʎi* (Sevortyan et al. 1974–: vol. 5: 78; Tenishev 2001: 325; Dybo 2013: 422), attested in all Nuclear Turkic subgroups, but absent in Chuvash. Chuvash **ʆɨn* ‘person’ lacks reliable Nuclear Turkic cognates (cf. Dybo 2013: 423).

Proto-Mongolic. **kühün* ~ **kümün* (Nugteren 2011: 437; Gruntov & Mazo 2015: 236), attested as a basic term in all three subgroups.

Proto-Tungusic. The adjacent concepts ‘person’ and ‘man’ are relatively unstable in Tungusic. Note that, in the Evenic subgroup, inherited terms for these meanings were almost completely replaced with reflexes of the virtual form **beye* (Tsintsius 1975: 122–123; Starostin, Dybo & Mudrak 2003: 335), likely to have been borrowed from Mongolic **beye* ‘body; self’ (Nugteren 2011: 281). Say, in Nanai *beye* means simply ‘body, figure; self’, but in the Evenic lects its meaning was expanded with the trivial semantic shift ‘body > person, man’: Negidal ‘person; man; body’, Evenki ‘person; man; husband’, Even ‘person; man’. If Tungusic **beye* is ruled out as a loan, an appropriate candidate for ‘person’ is **ɲiari* (Tsintsius 1975: 598–599; Starostin, Dybo & Mudrak 2003: 1013), meaning ‘person’ in Nanaic, Udiheic and Manchu-Jurchen. This root is also attested in the Evenic subgroup as ‘man; young man, youth’ (Even *ɲari*, probably not a basic term for ‘man’ in that language) and as the suffixed stem **ɲiari-bi:* ‘young man, youth’ (Evenki, Negidal).

The concept ‘man’ is more problematic. A possible candidate for Proto-Tungusic ‘man’ is **kaka* (Tsintsius 1975: 459, unconvincingly connected with **aka-* ‘elder brother’ in Starostin, Dybo & Mudrak 2003: 281): it means ‘man’ in Manchu-Jurchen, having been retained in Udiheic as the name of a mythic hero (master of rain and thunder) or simply as the adjective ‘brave, courageous’ in Nanaic. The second candidate for ‘man’ is **xüse* (Tsintsius 1975: 332; Starostin, Dybo & Mudrak 2003: 829). Its common Nanaic meaning is ‘male (of animal)’: indeed in Ulch or Orok **xüse* acquires the additional meaning ‘man’; the Nanai collocation for ‘man’, *xuse nay*, lit. ‘male person’, suggests that the meaning ‘male’ should be primary at least for the Proto-Nanaic level. In the Evenic subgroup, the suffixed stem **xüse-gde* ‘hunter’ is retained (Tsintsius also glosses these Evenic forms as ‘person’, but synchronous dictionaries do not confirm it). When choosing between **kaka* and **xüse*, the former seems a more reliable candidate for ‘man’, whereas **xüse* could be reconstructed as ‘male (of animal)’, but nevertheless we provisionally prefer to fill the slot ‘man’ with both forms, **kaka* and **xüse*.

Middle Korean. *sǎːr-ʌ́m* (Starostin, Dybo & Mudrak 2003: 1511).

Proto-Japonic. **pítə̀* (Starostin, Dybo & Mudrak 2003: 1103), **pítò* (Vovin 1999: 89). Attested in Japanese and Ryukyuan.

65. ‘rain’

Proto-Turkic. **yag-mur* (Sevortyan et al. 1974–: vol. 4: 57; Tenishev 2001: 25; Dybo 2013: 425), attested in all subgroups and in Ancient Turkic sources. The cognate verb is **yag* ‘to rain’, but the morpheme **mur* is not attested elsewhere. In some Nuclear Turkic lects, regular deverbatives **yag-ɨn* or **yag-iš* ‘rain’ are introduced instead.

Proto-Mongolic. **kura* (Nugteren 2011: 433; Gruntov & Mazo 2015: 236), attested as a basic term in all three subgroups. In the majority of the Northern lects, was superseded by or coexists with **boruha-n* ‘rain’ (Nugteren 2011: 285; Gruntov & Mazo 2015: 236). The original Proto-Mongolic meaning of **boruha-n* was probably ‘snow storm (vel sim.)’ as attested in Middle Mongolian. Mogholi *bɔrɔn* ‘rain’ was likely reborrowed from Persian.

Proto-Tungusic. There are two candidates. (1) **tük-de* (Tsintsius 1977: 175; Kazama 2003: 45; Starostin, Dybo & Mudrak 2003: 1442), used as a basic term in all non-Manchu-Jurchen subgroups, not attested in Manchu-Jurchen. A deverbative from **tük-* ‘to fall’ (Tsintsius 1977: 177–178). (2) **aga* (Tsintsius 1975: 11; Kazama 2003: 45; Starostin, Dybo & Mudrak 2003: 273), a basic term in Manchu-Jurchen, not attested elsewhere. If Manchu-Jurchen is indeed the first outlier, the primary stem **aga* clearly has the advantage, but because of the unresolved issue with classification it is more prudent to treat **aga* and **tük-de* as technical synonyms.

Middle Korean. *pí* (Starostin, Dybo & Mudrak 2003: 1146).

Proto-Japonic. **àmâi* (Starostin, Dybo & Mudrak 2003: 1025), **àmâ-Ci* (Vovin 1999: 89). Attested in Japanese and Ryukyuan, with very common polysemy ‘sky / rain’.

Etymological notes. Turkic **yag-*, Korean *pí* < Proto-Altaic **pʰi̯àge* (Starostin, Dybo & Mudrak 2003: 1146). Correspondences seem regular. The original semantics of the Turkic root **yag* could be either verbal or nominal. The second element *mur* in Turkic **yag-mur* 'rain' is explained by Starostin, Dybo & Mudrak 2003: 935 as a relic reflex of Proto-Altaic **mi̯ù:ri* 'water' which is not excluded. Robbeets does not consider this etymology.

66. 'red'

Proto-Turkic. **kɨrʸ-ɨl* (Sevortyan et al. 1974–: vol. 6: 194; Tenishev 2001: 602; Dybo 2013: 428), attested in all subgroups. Derived from the verb **kɨ:rʸ* 'to become red-hot' (Sevortyan et al. 1974–: vol. 6: 187; Dybo 2013: 428) with irregular shortening of the root vowel.

Proto-Mongolic. **hula-han* (Nugteren 2011: 363; Gruntov & Mazo 2015: 236), attested as a basic term in all three subgroups.

Proto-Tungusic. **pula-* (Tsintsius 1977: 343–344; Kazama 2003: 128; Starostin, Dybo & Mudrak 2003: 1109), apparently a verbal root 'to be red', which only survived in various participles and deverbatives with the meanings 'red', 'reddish', 'a k. of berry', 'fox' and so on (traces of this verb appear in almost all Tungusic languages). Specific adjectival stems meaning 'red' are based on **pula-* in Evenic, Udiheic (Udihe) and Manchu-Jurchen, although suffixal patterns differ even between the Evenic lects. In addition, there is also the stem **se:g-(g)ǯe* (Tsintsius 1977: 136; Kazama 2003: 128; Starostin, Dybo & Mudrak 2003: 1224), a deverbative from the relict verbal stem **se:gV-* 'to bleed, flow (of blood)'; see further notes on **se:g-kse* 'blood'. This is a basic term for 'red' in Nanaic and also in Oroch, but its specific meaning 'blood-red' in Evenki apparently reflects the original semantics.

Middle Korean. *pìrk-* (Starostin, Dybo & Mudrak 2003: 1109).

Proto-Japonic. **áká-* (Starostin, Dybo & Mudrak 2003: 598), **àkà-* (Vovin 1999: 89).

Etymological notes. Mongolic **hula-han*, Tungusic **pula-*, Korean *pìrk-* < Proto-Altaic **puli* (Starostin, Dybo & Mudrak 2003: 1109; Robbeets 2005: 406–407; Robbeets & Bouckaert 2018). Correspondences seem regular, assuming a fossilized *k*-suffix in Korean.

67. 'road'

Proto-Turkic. **yo:l* (Sevortyan et al. 1974–: vol. 4: 217; Tenishev 2001: 531; Dybo 2013: 430), attested as a basic term in all subgroups except for Tofa-Tuvinian.

Proto-Mongolic. An interesting case with two main candidates. (1) **mör* 'road' (Nugteren 2011: 448; Gruntov & Mazo 2015: 236), a basic term for 'road' in the Southern subgroup, usually with polysemy 'road / track, trace', meaning simply 'track, trace, trail', 'path', 'furrow (in the field)' in the Northern subgroup. (2) **kargui* (Gruntov & Mazo 2015: 236), meaning 'road' in some Northern lects (Khamnigan, Buriat, as a relict in Khalkha), probably not attested elsewhere. Note that the majority of the Northern lects has a Turkic loan for this concept, whereas Dagur has a transparent innovation (Gruntov & Mazo 2015: 237).

The first term, **mör*, has the advantage in two respects. First, it is attested in two subgroups and thus is more ancient from the formal point of view. Second, the semantic shift ‘road’ > ‘path’ is very common cross-linguistically, so it is likely that **mör* meant ‘road; path; track, trace’ in Proto-Mongolic having narrowed into the meanings ‘track, trace’, ‘path’ in the Northern subgroup.

Proto-Tungusic. **pokta* (Tsintsius 1977: 331; Kazama 2003: 41; Starostin, Dybo & Mudrak 2003: 1166), attested as a basic term in non-Manchu-Jurchen subgroups and retained in Manchu as the derivative *oktoron* ‘hare tracks’ (if this form exists). In Manchu-Jurchen, superseded with virtual **ǯugu-n* (Tsintsius 1975: 269; Kazama 2003: 41), apparently a derivative from a Mongolian loan, cf. Evenki *ǯuɣu*, Solon *ǯug* ‘direction’ < Mongolian **ǯüg* ‘direction, side’ (Nugteren 2011: 394). Less convincingly in Starostin, Dybo & Mudrak 2003: 1542, where Manchu-Jurchen **ǯugu-n* is derived from Tungusic **ǯuku-* ‘corner’ (Tsintsius 1975: 262) with an unusual semantic shift and irregular voicing **k* > *g*.

Middle Korean. *kìrh* (Starostin, Dybo & Mudrak 2003: 546).

Proto-Japonic. **mítí* (Starostin, Dybo & Mudrak 2003: 930). Attested in Japanese and Ryukyuan. According to Robbeets (2005: 95), Vovin (2010: 200) and other specialists, the word may be analyzed as **mi-ti* (containing the productive honorific prefix **mi-*) due to the “simple” root **ti* occurring in such compound forms as *ti-mata* ‘road fork’, etc. However, the bisyllabic form is clearly reconstructible for Proto-Japonic (due to Ryukyuan evidence), and there is no internal Japanese evidence for the form **miti* being originally applied to anything specifically deserving of honor (e.g. “large /Imperial/ road”, etc.). These considerations require the acceptance of the bisyllabic variant as primary, while monosyllabic **ti* may either be a different root or the result of later morphological reanalysis of **miti* as **mi-ti* already after the disintegration of Japonic.

Etymological notes. Mongolic **mör*, Japonic **mítí* < Proto-Altaic **mi̯óri* (Starostin, Dybo & Mudrak 2003: 930), correspondences seem regular except for the fact that the distribution between Japonic **r* and **t* as an outcome of Proto-Altaic **r* remains unclear. Robbeets (2005: 95, 403) analyzes the Japonic form as a prefixed derivative from **ti* ‘road’ and keeps it apart from the Mongolic one; however, only the bisyllabic form **mítí* is safely reconstructible for Proto-Japanese-Ryukyuan, and the original prefixal status of **mí-* is rather dubious..

68. ‘root’

Proto-Turkic. **dạmor* (Sevortyan et al. 1974–: vol. 3: 143; Tenishev 2001: 107; Dybo 2013: 433) means ‘blood vessel’ in all subgroups and has the additional meaning ‘root’ in Chuvash and the majority of the Nuclear Turkic subgroups (Tofa-Tuvinian, South Siberian, Karluk, Kipchak, as a relic in Altay), thus the polysemy ‘blood vessel / root’ should be reconstructed for Proto-Turkic.

Proto-Mongolic. There are two Proto-Mongolic terms intertwined between the lects and subgroups, **ünde-sün* (Nugteren 2011: 541; Gruntov & Mazo 2015: 237) and **hiǯa-hur* (Nugteren 2011: 362; Gruntov & Mazo 2015: 237). As noted by Gruntov and Mazo, the two terms are synchronically opposed in Dagur and some Khalkha dialects as ‘root in the soil’ (**ünde-sün*) and ‘root above the ground’ (**hiǯa-hur*). This is probably the key to the abnormal distribution of the two stems and the aforementioned semantic opposition is to be projected onto the Proto-Mongolic level. We fill the slot with **ünde-sün* ‘root in the soil’.

Proto-Tungusic. Not a very stable concept. An additional difficulty is that it is not always clear whether the gloss 'root' in a source refers to a botanical term or expresses the metaphoric meaning 'origin, basis'. The main candidate is **daga* (Tsintsius 1975: 188–189; Starostin, Dybo & Mudrak 2003: 1348). In its plain form, it is attested as a basic term in Manchu (*da:* 'root of tree, plant') and maybe in archaic Oroch (if Leontovich's *taha* 'root' is to be amended to *daha*); as not a basic term in Negidal (*da:* 'root, butt of trunk; beginning'). Its various suffixal derivatives are used as basic words for 'root' in Nanaic (Nanai *da:-čan*, Ulch *da:-ni*, Orok *da:-tan*). Further Evenki *daɣa-ča:n* 'root, stub, butt of tree' (not a basic term). A weaker candidate is **ŋü:ŋte* (Tsintsius 1975: 662; Starostin, Dybo & Mudrak 2003: 1033), attested in non-Manchu-Jurchen subgroups. It means 'root' in general in Evenic (everywhere) and Udiheic (Udihe), and specifically 'thin root (of tree, plant)' or 'root of plant' in Nanaic (Nanai, Orok) and Udiheic (Oroch). The most parsimonious solution is to reconstruct Proto-Tungusic **daga* 'root (of tree)' and **ŋü:ŋte* 'root (of plant)'.

Middle Korean. *pùrhúy* (Starostin, Dybo & Mudrak 2003: 1182).

Proto-Japonic. **nài* (Starostin, Dybo & Mudrak 2003: 965), **nE* (Vovin 1999: 89). Attested in Japanese and Ryukyuan. An additional candidate is **mə̀tə̀* (Starostin, Dybo & Mudrak 2003: 1033), **mòtò* (Vovin 1999: 89), but it is weaker for two reasons: (a) it is more often used in the abstract sense of 'foundation, basis' and (b) it is notably missing in Ryukyuan.

69. 'round'

Proto-Turkic. Very unstable items prone to borrowing (see the table in Dybo 2013: 445). The lexical opposition 'round 2D' / 'round 3D' is characteristic of the majority of Turkic lects and thus is likely to be reconstructed for Proto-Turkic. Usually synchronic terms for 'round' represent new formations from 'disk, wheel', 'to turn around' etc. Out of these roots, one one worth mentioning is **tekör* 'disc' (Dybo 2013: 438), phonetically similar to **tegir-* 'to surround' (Sevortyan et al. 1974–: vol. 2: 178; Dybo 2013: 440); both serve as a base for 'round (usually 2D)' in various Nuclear Turkic lects. Cf. also the bound root **yum* (Sevortyan et al. 1974–: vol. 4: 246; Dybo 2013: 441), whose various derivatives means 'round (usually 3D)' in Nuclear Turkic; the original meaning of **yum* is unclear, but such a derivative as **yum-gaq* 'round 3D' (attested in Ancient Turkic) suggests its substantive status, thus probably 'ball *vel sim.*'. We prefer to leave the slot empty.

Proto-Mongolic. Not reconstructible. Usually expressed with either loans or etymological isolates (Gruntov & Mazo 2015: 237).

Proto-Tungusic. An unstable item which cannot be reconstructed with certainty. The lexical opposition 'round 3D' / 'round 2D' is characteristic of non-Manchu-Jurchen subgroups, but expressions differ between individual languages. The most widespread pattern for 'round 2D' is various deverbatives from **murV-* 'to walk around, go right around' (Tsintsius 1975: 559–560; Starostin, Dybo & Mudrak 2003: 955) attested in Evenic (Even *mere:-ti*, Evenki *muru-me*, Negidal *meye-l-meye-l*) and Nanaic (Nanai *muru-muru*, Orok *moro-li*) as well as in Manchu (*mur-gʸen*). Inconsistency in suffixes, however, suggests that we are dealing with a series of late introductions. The majority of other terms for 'round 2D' or '3D' also represent various new formations.

Middle Korean. *tùr-y-ə́β-* (Starostin, Dybo & Mudrak 2003: 1379).

Proto-Japonic. **márə̀* (Starostin, Dybo & Mudrak 2003: 955), **márú* ~ **máró* (Vovin 1999: 89).

70. 'sand'

Proto-Turkic. **kum* ~ **kum-ak* (Sevortyan et al. 1974–: vol. 6: 133; Tenishev 2001: 102; Dybo 2013: 447), attested as a basic term in all Nuclear Turkic subgroups. The second candidate is **kạyɨr* (Sevortyan et al. 1974–: vol. 5: 217; Tenishev 2001: 97; Dybo 2013: 448), meaning 'sand' in Chuvash, some Kipchak lects and an Ancient Turkic language (Karakhanid). In Kipchak and Karakhanid, **kạyɨr* coexists with **kum* in meaning 'sand', the synchronic difference is unclear. In other Nuclear Turkic subgroups, **kạyɨr* can denote 'gravel', 'coarse sand' etc. Most likely the Proto-Nuclear Turkic meaning of **kạyɨr* was 'gravel, coarse sand' as opposed to basic **kum* 'sand'. The existence of **kum-ak* 'sand' in Bulghar is proven by the Bulghar loan *homok* 'sand' in Hungarian. In light of this, **kum* can be safely posited as the basic term in Proto-Turkic.

Proto-Mongolic. **ele-sün* (Nugteren 2011: 327; Gruntov & Mazo 2015: 238), attested as a basic term mainly in the Northern subgroup, but also in Middle Mongoilan sources and as a relic in Dagur.

Proto-Tungusic. The main candidate seems to be **xoɲi-* (Tsintsius 1977: 20; Starostin, Dybo & Mudrak 2003: 839) modified with various desemanticized suffixes and attested as a basic term for 'sand' in all four subgroups: Evenic (Even *oni-ŋ*), Udiheic (Udihe *oɲo-kto* 'sand', further Oroch *oɲo-kto* 'dirt'), Nanaic (Orok *xoŋo-kto*) and finally Manchu *yoŋ-ga-n* 'sand' (*ŋg* regularly < **ɲ-g*). In Tsintsius 1975: 320; Starostin, Dybo & Mudrak 2003: 588, this Manchu word is assigned to **(x)iŋa:* 'coarse sand', but such a solution is clearly less preferable both semantically and phonetically (**i* should yield Manchu *i*, not *o*). The second candidate is **siru-* (Tsintsius 1977: 96; Starostin, Dybo & Mudrak 2003: 1269) which is also widely attested as a basic term for 'sand': Evenic (Negidal, Evenki) Udiheic (Oroch), Nanaic (Nanai, Ulch) and Jurchen *sirxe* 'sand'. Besides the criss-crossed configuration, the main difficult with **siru-* is that it is readily borrowed from one lect to another as follows from the coexistence of different variants in one language: Oroch *siya* ~ *siru* 'sand', Nanai *siro-n* ~ *siya:-n* 'sand', Ulch *siru* ~ *siya-n* 'sand' (normally **r* > *y* in Udihe-Oroch and Negidal and > *r* elsewhere). If Jurchen *sirxe* is a suffixed cognate of other forms, we have to treat **xoɲi-* and **siru-* as technical synonyms. Otherwise, if the Jurchen form is a chance coincidence with the **siru-* forms, the most probable scenario that **siru-* with the meaning 'sand' was a local innovation which then spread via contacts. Distinct from Evenic-Udiheic **(x)iŋa:* 'coarse sand, gravel' (Tsintsius 1975: 320–321; Starostin, Dybo & Mudrak 2003: 588).

Middle Korean. *mòr-ʔáy* (Starostin, Dybo & Mudrak 2003: 904).

Proto-Japonic. **súná* (Starostin, Dybo & Mudrak 2003: 1293, Vovin 1999: 89). Attested in Japanese and Ryukyuan. Probably to be analyzed as **sú-ná*, where **-ná* = 'earth' (Robbeets 2005: 101, although her identification of **sú-* with the auxiliary morpheme 'plain, simple' is rather questionable in this case).

71. 'to say'

Proto-Turkic. **dẹ̄-* (Sevortyan et al. 1974–: vol. 3: 221; Dybo 2013: 451), attested as basic 'to say' in Chuvash and some Nuclear Turkic subgroups (e.g., Yakut, Kipchak). Distinct from **ạy-, *ạy-ït-* 'to tell' (Sevortyan et al. 1974–: vol. 1: 99, 111; Dybo 2013: 452).

Proto-Mongolic. There are two frequent verbs of speech which usually coexist in the attested languages: (1) **kehe-* (Northern, Southern) (Nugteren 2011: 408; Gruntov & Mazo 2015: 238), modern languages point to the protoform **ge-*, probably a result of occasional reduction in the final phrasal position. (2) **kele-le-* (Northern, Southern, Dagur) (Nugteren 2011: 410; Gruntov & Mazo 2015: 238), regularly derived from **kele-n* 'tongue' with an occasional haplology **kelele-* > **kele-* in modern languages (the shape *kelele-* is attested in Middle Mongolian). Descendants of both verbs can be used with the meaning 'to say', but **kele-le-* also frequently means 'to speak', so the Proto-Mongolic opposition may tentatively be reconstructed as **kehe-* 'to say' vs. **kele-le-* 'to speak'.

Proto-Tungusic. **guːn-* (Tsintsius 1975: 171; Starostin, Dybo & Mudrak 2003: 571, partially incorrectly in Kazama 2003: 81), attested as a basic term in Evenic (Even, Evenki, Negidal), Udiheic (Udihe, Oroch) and Jurchen (later shifted into the meaning 'to think' in Manchu). In Nanaic, superseded with **un-* of unclear origin.

Middle Korean. *nìrʔ- ~ nìrʌ̀-* (Starostin, Dybo & Mudrak 2003: 973).

Proto-Japonic. **íp-* (Starostin, Dybo & Mudrak 2003: 589), **ip-* (Vovin 1999: 89). Attested in Japanese and Ryukyuan.

72. 'to see'

Proto-Turkic. **gör* (Sevortyan et al. 1974–: vol. 3: 77; Dybo 2013: 455), attested as a basic term in all subgroups.

Proto-Mongolic. **üǯe-* (Nugteren 2011: 540; Gruntov & Mazo 2015: 239), attested as a basic term in all three subgroups. Distinct from **kara-* 'to look' (Nugteren 2011: 404); note that Dagur uses **üǯe-* for both meanings.

Proto-Tungusic. **iče-* (Tsintsius 1975: 334–335; Starostin, Dybo & Mudrak 2003: 579; Kazama 2003: 80), used as a basic term in all non-Manchu-Jurchen languages (not attested in Manchu-Jurchen). The Jurchen verb 'to see' is *hača* (> Manchu *ača-* 'to meet' without further etymology) which could reflect an original stem **xača-*. If Manchu-Jurchen is indeed the first outlier, **iče-* and **xača-* are equiprobable candidates, so we take them as technical synonyms.

Middle Korean. *pó-* (Starostin, Dybo & Mudrak 2003: 323).

Proto-Japonic. **mì-* (Starostin, Dybo & Mudrak 2003: 981, Vovin 1999: 89). Attested in Japanese and Ryukyuan. Same root as ʽeye' q.v.

73. 'seed'

Proto-Turkic. **ur-ug* (Sevortyan et al. 1974–: vol. 1: 604; Tenishev 2001: 115; Dybo 2013: 464), attested as a basic term in Chuvash, Karluk, Kipchak and in Ancient Turkic (Clauson 1972: 214); frequently with polysemy 'seed (botanic) / progeny, descendants / clan, kin', but not in Chuvash, so there is no formal ground to reconstruct such a polysemy for Proto-Turkic. A standard derivative from the bound root **ur* not attested elsewhere except for the analogous(?) new formation **ur-luk* 'seed' in Kipchak (Sevortyan et al. 1974–: vol. 1: 604; Tenishev 2001: 116; Dybo 2013: 464).

Proto-Mongolic. **hüre-n* (Nugteren 2011: 370; Gruntov & Mazo 2015: 239), attested as a basic term in all three subgroups.

Proto-Tungusic. **üse* (Tsintsius 1975: 332; Tsintsius 1977: 290; Starostin, Dybo & Mudrak 2003: 623; Kazama 2003: 40), used as a substantive 'seed' in Jurchen-Manchu, Nanaic (Nanai, Ulch), Udiheic (Oroch), and as a verb 'to grow' in Evenic, Udiheic (Udihe). In the meaning 'seed', it tends to be superseded with Russian or Mongolian loans. Even if Tungusic **üse* is eventually of Mongolic origin, the semantics 'seed' is an inner Tungusic development. Kazama 2003: 40 suspects that the forms *use, usi* 'seed' in non-Jurchen-Manchu subgroups were actually borrowed from Manchu *use* 'seed' which makes the reconstruction **üse* 'seed' even more dubious. Nevertheless there are no formal reasons not to project **üse* 'seed' onto the Proto-Tungusic level.

Middle Korean. *psí* (Starostin, Dybo & Mudrak 2003: 1091).

Proto-Japonic. **tànài* (Tower of Babel project), **tàná-Ci* (Vovin 1999: 89). Attested in Japanese and Ryukyuan. The word is sometimes supposed to be of Austronesian origin (Starostin 1991: 115). Differently in Robbeets (2005: 102), who suspects a compound origin from *ta* 'rice' and *ne* 'root' (not very convincing on semantic grounds).

Etymological notes. Turkic **ur-ug*, Mongolic **hüre-n* < Proto-Altaic **pʰùri* (Starostin, Dybo & Mudrak 2003: 1187). Correspondences seem regular. Robbeets does not consider this etymology.

74. 'to sit'

Proto-Turkic. **ol-ur* ~ **ol-tur* (Sevortyan et al. 1974–: vol. 1: 489; Dybo 2013: 468), attested as a basic term in all subgroups. Formally a causative from the verbal root **ol*, not attested elsewhere. This implies that the Pre-Proto-Turkic meanings of **ol* and **ol-(t)ur* might have been 'to sit' and 'to sit down' respectively; later Proto-Turkic **ol-(t)ur* acquired the general stative meaning 'to sit'.

Proto-Mongolic. **sahu-* (Nugteren 2011: 484; Gruntov & Mazo 2015: 239), attested as a basic term in all three subgroups.

Proto-Tungusic. **tege-* (Tsintsius 1977: 226–228; Kazama 2003: 92; Starostin, Dybo & Mudrak 2003: 1410), attested as a basic term in all four subgroups.

Middle Korean. *ànč-* (Starostin, Dybo & Mudrak 2003: 302).

Proto-Japonic. **b(u)í-* (Starostin, Dybo & Mudrak 2003: 342), *bí- (Vovin 1999: 89). Attested in Japanese and Ryukyuan.

75. ‘skin’

Proto-Turkic. **dẹri* (Sevortyan et al. 1974–: vol. 3: 207; Tenishev 2001: 383; Dybo 2013: 470), attested as a basic term in all subgroups except for Tofa-Tuvinian.

Proto-Mongolic. **ara-sun* (Nugteren 2011: 272; Gruntov & Mazo 2015: 239), attested as a basic term in all three subgroups.

Proto-Tungusic. An unclear situation with two competing stems (Kazama 2003: 22). The first candidate is **xere-kte* (Tsintsius 1977: 467; Starostin, Dybo & Mudrak 2003: 782), a basic term for ‘human skin’ in non-Manchu-Jurchen subgroups (shifted to ‘bark’ in Evenki). From the formal point of view, this a derivative from the root **xere-*, retained in Nanaic as Ulch *xere-* ‘to remove scales from dried fish’ and Orok *xere* ‘yukola (dried fish meat)’. The root is not known in Manchu-Jurchen. In non-Manchu-Jurchen subgroups, **xere-kte* ‘human skin’ is opposed to **nansa* ‘animal skin’ (Tsintsius 1975: 583–584; Starostin, Dybo & Mudrak 2003: 962; without reliable Manchu-Jurchen cognates) and **subgu* ‘fish skin’ (Tsintsius 1977: 115, 116; Starostin, Dybo & Mudrak 2003: 1270). The opposition between the three terms is usually stable in non-Manchu-Jurchen subgroups, although **nansa* tends to acquire the generic meaning ‘human/animal skin’ in some lects (e.g., Negidal, Udihe). Differently in Manchu-Jurchen, where **subgu* is a generic term for ‘human/animal skin’. Various scenarios are possible, but, if Manchu-Jurchen is indeed the first outlier, the most parsimonious one is that **subgu* meant ‘human/animal/fish skin’ in Proto-Tungusic and then narrowed in the meaning ‘fish skin’ after the Manchu-Jurchen split-off. If the root **xere-* indeed meant ‘to skin (*vel sim.*)’, the original meaning of its derivative **xere-kte* could hardly be ‘human skin’, thus **xere-kte* ‘human skin’ should be a semantic innovation of the non-Manchu-Jurchen subgroups. Nevertheless, despite the above discussion, we accept both **subgu* and **xere-kte* as technical synonyms.

Middle Korean. *kàčòk* ~ *kàčʰ* (Starostin, Dybo & Mudrak 2003: 627).

Proto-Japonic. **kapa* (Starostin, Dybo & Mudrak 2003: 764). Attested in Japanese and Ryukyuan. Same word as ‘bark’ (see above).

76. ‘to sleep’

Proto-Turkic. **uː-ðɨ* ~ **u-ðɨ* (Sevortyan et al. 1974–: vol. 1: 579; Dybo 2013: 473), occurs as a basic term ‘to sleep’ in Yakut-Dolgan, Tofa-Tuvinian, South Siberian, some Oghuz lects as well as in Ancient Turkic sources (Clauson 1972: 42), where the deverbative **u(ː)ðɨ-g* ‘sleepy, asleep’ is attested as well (Clauson 1972: 46). The available forms do not allow to establish the vowel quantity in Proto-Turkic. In Chuvash, **u(ː)-ðɨ* was superseded with a denominative from **yabaĺ* ‘calm, quiet’ (Sevortyan et al. 1974–: vol. 4: 51; Dybo 2013: 474), a transparent innovation. The morphological analysis of the stem **u(ː)(-)ðɨ* requires comments. We find sufficient evidence to single out the rare and archaic denominative suffix **-ðV-* (Räsänen 1957: 145) and connect **u(ː)-ðɨ* with the Proto-Turkic substantive **uː* ‘sleep, dream’ and its derivative **uː-y-kɨ* with the same substantive meaning (medial *-y-* represents a morphophonological effect in the position when a root in a long vowel is modified with a consonant suffix). The latter stem also serves

as the basis for the denominative verb *u:yuk-la*, used in the basic meaning 'to sleep' in Karluk, Kipchak, Oghuz, Altay.

Proto-Mongolic. **unta* (Nugteren 2011: 532; Gruntov & Mazo 2015: 239), attested as a basic term in all three subgroups.

Proto-Tungusic. The adjacent concepts 'to lie' and 'to sleep' are rather unstable in Tungusic. One good candidate for 'to sleep' is **a:w-* (Tsintsius 1975: 2; Starostin, Dybo & Mudrak 2003: 307) - a basic term 'to sleep' in Evenic (Evenki, Negidal) and Nanaic (Nanai, Ulch, Orok; polysemy 'to sleep / to lie' in Nanai), but not attested in Manchu-Jurchen. S. Starostin et al. reconstruct it as **a:b-*, but the reflexes of the root final element in the modern languages (*w, u* or zero) significantly differ from those of **b*, so it is reasonable to introduce a phoneme like *w* for this case, cf. the similar development in **ǯuwer* 'two' q.v. Ryzhkov-Shukumine (2020: 74) prefers to introduce a *u*-diphthong here: **au-* 'to sleep'. The second candidate for 'to sleep' is the verb **dedu-* (Tsintsius 1975: 227, 230), meaning 'to sleep' in Jurchen and 'to lie' in Manchu (the Manchu verb 'to sleep' is a secondary derivative from **a:m-* 'to be sleepy'). Suffixal derivatives of **dedu-* mean 'flooring, deck' in Nanaic. In Starostin, Dybo & Mudrak 2003: 466, it is analyzed as **de-du-* < **de:-* in order to compare it with Oroch *de:* 'bed', but the assumed extension **-du-* remains unparalleled. Cf. also **ŋu(y)a-* (Tsintsius 1975: 597, 611, 636, 666; Starostin, Dybo & Mudrak 2003: 1038), basic 'to sleep' in Udiheic (Udihe and probably Oroch), its suffixal derivatives are also attested in Evenki ('to sleep fast') and Manchu ('to be lulled to sleep') - despite the promising external etymology proposed in Starostin, Dybo & Mudrak 2003: 1038, there is no firm reason to posit **ŋu(y)a-* as a basic Proto-Tungusic verb 'to sleep'. Cf. **pukele:-* (Tsintsius 1977: 340, 342; Starostin, Dybo & Mudrak 2003: 1168) which means 'to lie' or 'to lie; to sleep' in the Evenic subgroup, but its Proto-Tungusic meaning should be reconstructed as 'to roll down' or 'to fall' as follows from the Nanaic, Udiheic and Manchu-Jurchen data. As for 'to lie', there are no good candidates. The meaning 'to lie' can be expressed with the help of etymologically isolated verbs (Even *desči-* 'to lie', Tsintsius 1975: 238), Mongolian loans (Udiheic), or verbs with eventually different meanings (**a:b-* 'to sleep' in Nanai, **pukele:-* in Evenki). The only match between the subgroups is the exact morphological correspondence Orok *a:-pa-qat-* 'to lie' / Negidal *a:-pu-xat-* 'to lie' < **a:w-* 'to sleep', but these stems can represent parallel new formations. Summing up, we fill the slot 'to sleep' with **a:w-* and prefer to leave the slot 'to lie' empty. Note the stem **de(-)du-* which is also a possible candidate for either 'to sleep' or 'to lie'.

Middle Korean. *čá-* (Starostin, Dybo & Mudrak 2003: 1543).

Proto-Japonic. **úi-* (Starostin, Dybo & Mudrak 2003: 1038), **ui* (Vovin 1999: 89). Attested in Japanese proper. The root is nominal in nature ('sleep, dream /n./'), usually taking on the verbal meaning 'to sleep' in conjunction with the verbal extension **na-* 'to lie' q.v.: **úi-na-* 'to sleep'. In Ryukyuan dialects, the same verbal meaning is usually expressed with secondary suffixal extensions of the same verb **na-*.

Etymological notes. We treat Turkic **u-ðï*, Tungusic **a:w-*, Japonic **úi-na* as root cognates. Mongolic **unta* may also be ultimately related, but this requires the assumption of a fossilized suffix **-nt-*, not supported by other data. Differently Starostin, Dybo & Mudrak 2003: 307, 1038, 1498, where less plausible etymological solutions are proposed for the Turkic, Mongolic and Tungusic verbs. Robbeets (2005: 354) accepts S. Starostin et al.'s (2003: 1038) solution connecting Japonic **i-* (our **úi-*), Tungusic **ŋua-*, Mongolic **nojir-* and Turkic **u:-*.

77. ‘small’

Proto-Turkic. **kičü-g* (Sevortyan et al. 1974–: vol. 5: 75; Dybo 2013: 476), attested as a basic term in all Nuclear Turkic subgroups as well as in Ancient Turkic sources (Clauson 1972: 696). In Chuvash, superseded with a Mongolic loan (Dybo 2013: 475).

Proto-Mongolic. **očü-ken* (Nugteren 2011: 537; Gruntov & Mazo 2015: 240), attested as a basic term in all three subgroups.

Proto-Tungusic. The best candidate is **ŋüši-* (Tsintsius 1975: 589–590; Kazama 2003: 114; Starostin, Dybo & Mudrak 2003: 1035), attested as a basic term in Evenic (Evenki dial., Negidal), Udiheic, Nanaic, and probably Jurchen (if Jurchen *osu-wan* ‘small’ and Manchu *isu-xun* ‘tiny’ are related).

Middle Korean. *čyǎːk-* ~ *čyǝ̌ːk-* (Starostin, Dybo & Mudrak 2003: 437).

Proto-Japonic. **tìpì-sà-* (Starostin, Dybo & Mudrak 2003: 448, Vovin 1999: 89); **-sa-* is a relatively common adjectival suffix. Attested in Japanese proper. The main Ryukyuan equivalent is **goma-* (Thorpe 1983: 330), a somewhat suspicious form because of initial **g-* (substrate lexeme?).

Etymological notes. Mongolic **očü-ken*, Tungusic **ŋüši-* < Proto-Altaic **ŋóːyču* (Starostin, Dybo & Mudrak 2003: 1035). Correspondences seem regular. Robbeets does not consider this etymology.

78. ‘smoke’

Proto-Turkic. **tüt-ün* (Tenishev 2001: 364; Dybo 2013: 479), attested as a basic term in Chuvash, Oghuz, Altay and some Ancient Turkic sources. Derived from the verb **tüt-* ‘to smoke’. In some Karluk, Oghuz and Kipchak lects, **tüt-ün* is superseded with the new formation **tüt-süg* ‘smoke’ from the same verb. Another candidate is the primary stem **ɨːʎ* (Tenishev 2001: 370; Dybo 2013: 480), which means ‘smoke’ in Tofa-Tuvinian, South Siberian, Altay; in other lects it can mean ‘invisible smoke (which irritates eyes in the room)’ or ‘soot’. It is therefore likely that the opposition **tüt-ün* ‘visible smoke (as of bonfire)’ vs. **ɨːʎ* ‘invisible smoke (which irritates eyes in the room)’ existed at least in Proto-Nuclear Turkic.

Proto-Mongolic. The first candidate is **huni-n* (Nugteren 2011: 364; Gruntov & Mazo 2015: 240), attested as a basic term in the Southern branch (incl. Middle Mongolic) and Dagur (acc. to main lexicographic sources). This stem is also retained in Buriat (Northern subgroup) where it means ‘heat from hot embers, smoke with sparks’ and ‘mist, haze’ (Selenka dialect). Its derivative **huni-har* ‘mist, haze’ is known from Northern and probably Dagur. The second candidate is **hutu-ha-n* (Nugteren 2011: 366; Gruntov & Mazo 2015: 241), attested as a basic term in Northern, Southern (East Yugur) and also in Dagur (where it coexists with **huni-n*, but it is likely that **hutu-ha-n* is a more marginal word for ‘smoke’). This is a regular deverbative from **hutu-* ‘to emit smoke’ (Nugteren 2011: 366). The primary stem **huni-n* may be regarded as a likely option for the Proto-Mongolic term for ‘smoke’, in which case the deverbative **hutu-ha-n* can be treated as a trivial innovation of the Northern subgroup which was later introduced into East Yugur and Dagur through the influence of the dominating adjacent Northern languages. A serious weakness of this

scenario is that it is typologically strange for a language to have a verb ‘to emit smoke’ (*hutu-) morphologically unrelated to the noun ‘smoke’ (*huni-n), but such a situation is not impossible in theory. The opposite scenario is less parsimonious, although it cannot be fully excluded: the deverbative *hutu-ha-n represents the Proto-Mongolic term for ‘smoke’, whereas *huni-n meant ‘haze, mist’ (as retained in the Northern subgroup and East Yugur from the Southern subgroup) and later shifted towards the meaning ‘smoke’ independently in the majority of the Southern lects and Dagur. For the moment, we take *huni-n as *hutu-ha-n technical synonyms.

Proto-Tungusic. **saŋŋa-n* (Tsintsius 1977: 63; Starostin, Dybo & Mudrak 2003: 1210), attested as a basic term in all four subgroups. The cognate verb is **saŋŋa-* ‘to smoke’ which is also widely attested.

Middle Korean. *nʌ́y* (Starostin, Dybo & Mudrak 2003: 974).

Proto-Japonic. **kái-n-púri* (Starostin, Dybo & Mudrak 2003: 685), **kái[-]npuri* (Vovin 1999: 89). Attested in Japanese proper; the Ryukyuan equivalent is the related (and also composite) form **kebusi* (Thorpe 1983: 330) < **kai-n-pusi*. Comparison of both forms allows to single out **kái-* as the original root for ‘smoke’, extended in different ways in different branches (perhaps to prevent homonymy with **kái* ‘hair’), though the precise meanings of the other components remain debatable.

79. ‘to stand’

Proto-Turkic. **dur* (Sevortyan et al. 1974–: vol. 3: 296; Dybo 2013: 482), attested as a basic term in all subgroups.

Proto-Mongolic. **bayi-* (Nugteren 2011: 277; Gruntov & Mazo 2015: 241), attested as a basic term in Southern Mongolic and Dagur, where it usually acquires additional meanings such as ‘to stop to do smth.’ or ‘to be situated, stay’. In the Northern branch, **bayi-* has narrowed its semantics to the meaning ‘to be situated, stay’, whereas the concept ‘to stand’ is expressed with the help of the verb **ǯogso* (Gruntov & Mazo 2015: 241), probably without cognates in two other subgroups.

Proto-Tungusic. **ili-* (Tsintsius 1975: 302–303; Kazama 2003: 92; Starostin, Dybo & Mudrak 2003: 583), attested as a basic term in all four subgroups.

Middle Korean. *syə́-* (Starostin, Dybo & Mudrak 2003: 1200).

Proto-Japonic. **tàt-* (Starostin, Dybo & Mudrak 2003: 404, Vovin 1999: 89). Attested in Japanese and Ryukyuan.

Etymological notes. Turkic **dur*, Japonic **tàt-* < Proto-Altaic **čùra* (Starostin, Dybo & Mudrak 2003: 404), correspondences seem regular except for the fact that the distribution between Japonic **r* and **t* as an outcome of Proto-Altaic **r* remains unclear. Robbeets (2005: 392; Robbeets & Bouckaert 2018) rejects this etymology comparing Japonic *tatu-* ‘to stand, rise, run high’ with Middle Korean *toT-* ‘to run’.

80. ‘star’

Proto-Turkic. **yul-dirʸ* (Sevortyan et al. 1974–: vol. 4: 279; Tenishev 2001: 53; Dybo 2013: 484), attested as a basic term in all subgroups. For the rare and archaic suffix **-dVrʸ* cf. Räsänen 1957: 113.

Proto-Mongolic. **hodu-n* (Nugteren 2011: 358; Gruntov & Mazo 2015: 241), attested as a basic term in all three subgroups.

Proto-Tungusic. **xo:si-kta* (Tsintsius 1977: 27; Kazama 2003: 47; Starostin, Dybo & Mudrak 2003: 845), attested as a basic term in all four subgroups.

Middle Korean. *pyə̌:r* (Starostin, Dybo & Mudrak 2003: 1155).

Proto-Japonic. **pə́sí* (Starostin, Dybo & Mudrak 2003: 1155), **pósí* (Vovin 1999: 89). Attested in Japanese and Ryukyuan.

Etymological notes. In Starostin, Dybo & Mudrak 2003: 1155, Mongolic **hodu-n* is deduced from Pre-Proto-Mongolic **hol-du-n* with the rare suffix **-du* and then compared with Turkic **yul-dirʸ*, Korean *pyə̌:r*, Japonic **pə́sí* < Altaic **pʰi̯óʎo* 'star'. Although such a comparison seems semantically likely, it faces certain difficulties since the clusters **ld, *ʎd* are assumed to be retained in Proto-Mongolic (Starostin, Dybo & Mudrak 2003: 84–85), Cf., however, even more similar cases in Mongolic: **modu-n* (< **mo(r)-du-n*?) 'tree', **nidü-n* (< **nil-dü-n*?) 'eye', **sidü-n* (< **sil-dü-n*?) 'tooth'. Robbeets (2005: 404; Robbeets & Bouckaert 2018) only accepts the Korean-Japonic comparison.

81. 'stone'

Proto-Turkic. **dia:ʎ* (Sevortyan et al. 1974–: vol. 3: 167; Tenishev 2001: 638; Dybo 2013: 486), attested as a basic term in all subgroups.

Proto-Mongolic. **čila-hu-n* (Nugteren 2011: 302; Gruntov & Mazo 2015: 242), attested as a basic term in all three subgroups; in Middle Mongolic, **čila-hu-n* means 'stone', but is more rarely used than its synonym **güri* ~ **küri* (for which see below). The second and definitely weaker candidate is **güri* ~ **küri* (Nugteren 2011: 347; Gruntov & Mazo 2015: 241), which functions as a basic term in some Southern lects, namely Middle Mongolic and Mogholi; in Northern, it usually means 'ironstone, ore'.

Proto-Tungusic. **ǯolo* (Tsintsius 1975: 263; Kazama 2003: 43; Starostin, Dybo & Mudrak 2003: 1373), attested as a basic term in all non-Manchu-Jurchen subgroups. In Manchu-Jurchen, superseded with a diminutive in **-k-* from **xure:* 'mountain' q.v.

Middle Korean. *tŏ:rh* (Starostin, Dybo & Mudrak 2003: 1373).

Proto-Japonic. **(d)ísì* (Starostin, Dybo & Mudrak 2003: 1373), **(d)ísò* (Vovin 1999: 89). Attested in Japanese and Ryukyuan. Initial **d-* may be assumed only inasmuch as word-initial **di- / *i-* (or **yi- / *i-* in the "conservative" variant of the reconstruction) were completely neutralized already in Proto-Japonic.

Etymological notes. Turkic **dia:ʎ*, Mongolic **čila-hu-n*, Tungusic **ǯolo*, Korean *tŏ:rh*, Japonic **(d)ísì* < Proto-Altaic **ti̯ó:ʎì* (Starostin, Dybo & Mudrak 2003: 1373). Correspondences seem regular. Robbeets

(2005: 87–88) does not accept this etymology and does not mention any Altaic *comparanda* for the Japonic form.

82. ‘sun’

Proto-Turkic. **gün ~ *gün-eʎ ~ *guɲ-aʎ* (Sevortyan et al. 1974–: vol. 3: 100; Tenishev 2001: 20; Dybo 2013: 488), attested as a basic term for ‘sun’ in all Turkic subgroups, frequently with additional meanings ‘day’ and/or ‘heat’ (sometimes these meanings are distributed among the morphological and phonological variants). Details are not clear, but it is likely that the observed morphological and phonological variants are due to contamination with the verb **köɲ* ‘to burn (intr., of fire, firewood)’ (Tenishev 2001: 362; Dybo 2013: 189) and its presumed deverbative ***köɲ-eʎ* ‘heat’. The suffix **-eʎ* / **-aʎ* has a diminutive meaning (Tenishev 1988: 14).

Proto-Mongolic. **nara-n* (Nugteren 2011: 452; Gruntov & Mazo 2015: 241), attested as a basic term in all three subgroups.

Proto-Tungusic. **siguː-n* (Tsintsius 1977: 78; Kazama 2003: 46; Starostin, Dybo & Mudrak 2003: 1274), attested as a basic term in all four subgroups.

Middle Korean. *hʌ́y* (Starostin, Dybo & Mudrak 2003: 1274).

Proto-Japonic. **pí* (Starostin, Dybo & Mudrak 2003: 1147, Vovin 1999: 89). Attested in Japanese proper, with polysemy ʻsun / day’. Different in Ryukyuan: *teda (Thorpe 1983: 336), possibly from an older form like *tianta-, but without any correlations in Japanese proper.

Etymological notes. Tungusic **siguː-n*, Korean *hʌ́y* < Proto-Altaic **si̯ògu* (Starostin, Dybo & Mudrak 2003: 1274). Correspondences seem regular. Robbeets does not consider this etymology.

83. ‘to swim’

Proto-Turkic. Generally an unstable item (Dybo 2013: 490–493), but there is nevertheless a reliable candidate: **yürʸ* (Sevortyan et al. 1974–: vol. 4: 261; Dybo 2013: 490), meaning ‘to swim’ in South Siberian, Karluk, Oghuz, Kipchak, Altay as well as in Ancient Turkic sources (Clauson 1972: 984).

Proto-Mongolic. Despite the general instability of the concept (see the overview in Gruntov & Mazo 2015: 242–243), there is a reliable candidate: **hunba* (tends to assimilate > **humba*; Nugteren 2011: 363; Gruntov & Mazo 2015: 242), attested as a basic term in all three subgroups (incl. Middle Mongolic). In individual lects, it acquires such additional meanings or shifts towards such new meanings as ‘to bathe’, ‘to ford’, ‘to wander through the water (or through tall grass)’. Other candidates are weaker and apparently represent some local developments, cf., e.g., the Northern stem **oyi-ma-* (Gruntov & Mazo 2015: 243), which is used as a basic term for ‘to swim’ in closely related Oirat and Kalmyk (these lects form a distinct clade) and as one of marginal verbs of swimming in Khalkha (the basic Khalkha verb goes back to **sele*, whose original meaning was likely ‘to row, paddle’, Gruntov & Mazo 2015: 243).

Proto-Tungusic. **elbe-s-* (Tsintsius 1977: 445; Starostin, Dybo & Mudrak 2003: 502), attested as a basic term in all four subgroups, although in some lects it shifts towards the meaning 'to bath' (Udihe, Nanai) or displays the polysemy 'to swim / to bathe' (e.g., Evenki, Manchu). Probably an old derivative from **elbe-* 'to cover' which is widely attested in Tungusic (Tsintsius 1977: 445). The derivation from a virtual verb **elbe-* 'to rake up' is less probable despite the general semantic plausibility since **elbe-* can only be reconstructed on the basis of an isolated dialectal Even form (Tsintsius 1977: 445). A weaker candidate is **pabri-* (Tsintsius 1975: 458; Starostin, Dybo & Mudrak 2003: 1071), attested in Evenic and Nanaic. In Nanaic, **pabri-* is a basic term for 'to swim' in Nanai & Ulch, it is also glossed as 'to swim (of animals); to bath, swim (of humans)' for Orok (but the Orok basic term 'to swim' goes back to **elbe-s-*). In Evenic, **pabri-* is glossed as 'to dive; to swim' for Evenki and 'to swim (of human)' for Negidal - the exact differences between these verbs and the reflexes of **elbe-s-* is unclear, but in both languages the basic terms for 'to swim' go back to **elbe-s-*.

Middle Korean. *hə̀y-* (Starostin, Dybo & Mudrak 2003: 1488).

Proto-Japonic. **ə̀yə̀-nk-* (Starostin, Dybo & Mudrak 2003: 1043), **òyò-* (Vovin 1999: 90). Attested in Japanese and Ryukyuan.

84. 'tail'

Proto-Turkic. **kudruk* (Sevortyan et al. 1974–: vol. 6: 114; Tenishev 2001: 145; Dybo 2013: 494), attested as a basic term in all Turkic subgroups.

Proto-Mongolic. **sehül* (Nugteren 2011: 487; Gruntov & Mazo 2015: 243), attested as a basic term in all three subgroups.

Proto-Tungusic. **xürgü* (Tsintsius 1975: 325; Starostin, Dybo & Mudrak 2003: 814), attested as a basic term at least in non-Manchu-Jurchen subgroups. Manchu *unče-xen* 'tail' is also likely to belong here, although the development *-nč-* < **-rg-* requires additional investigation.

Middle Korean. *skòrí*, possibly < Proto-Korean prefixed **s=kori*.

Proto-Japonic. **bə̀* (Starostin, Dybo & Mudrak 2003: 1037), **bò* (Vovin 1999: 90). Attested in Japanese proper; the Ryukyuan equivalent is unclear **zu(wo)* (Thorpe 1983: 338), where the second syllable may actually go back to **bə̀*, meaning that the word is originally a compound.

Etymological notes. Turkic **kudruk*, Tungusic **xürgü*, Korean *s=kòrí* < Proto-Altaic **kʰiúdo-(rgV)* (Starostin, Dybo & Mudrak 2003: 814). Correspondences seem regular, assuming fossilization of the suffix **-rgV* in Turkic and Tungusic and subsequent cluster simplification in Tungusic. For a possible analysis of Korean *skori* as the root **kori* modified with the fossilized prefix **s-*, see notes on Korean *s(-)pɨr* 'horn', *s(-)pyə́* 'bone'. Robbeets does not consider this etymology.

85. 'that'

Proto-Turkic. Reconstruction of the Proto-Turkic opposition of deictic pronouns is a non-trivial task which requires additional investigation. It is very likely that the Proto-Turkic system was ternary (proximal / medial / distal) rather than binary, but the details are not clear, since even synchronic systems of many modern Turkic languages are not described properly. At least the suppletive pronoun **ol* [nom.] / **V-n-* [obl.] can be reconstructed for medial/distal deixis, since it is attested in this function in all subgroups (Sevortyan et al. 1974–: vol. 1: 147, 444; Dybo 2013: 496, 498). The second likely candidate for medial/distal deixis is **ti* (Dybo 2013: 498), which is relatively widely attested in Nuclear Turkic including Yakut *i=ti* 'that'. We take **ol* and **ti* as synonyms. The proximal deixis pronoun can be reconstructed either as **bu* [nom.] / **bu-n-* [obl.] (Sevortyan et al. 1974–: vol. 2: 225; Dybo 2013: 500), attested in all Nuclear Turkic subgroups, but not in Chuvash, or **kö* (Dybo 2013: 501), only Chuvash. We take **bu* and **kö* as technical synonyms for 'this'.

Proto-Mongolic. The binary system **e-* 'this' (Nugteren 2011: 330; Gruntov & Mazo 2015: 244; Poppe 1955: 225) / **te-* 'that' (Nugteren 2011: 519; Gruntov & Mazo 2015: 243; Poppe 1955: 225) is stable in the attested languages and can be safely reconstructed for Proto-Mongolic.

Proto-Tungusic. The binary system **e-re* 'this' (Tsintsius 1977: 460–462; Starostin, Dybo & Mudrak 2003: 487) / **ta-re* 'that' (Tsintsius 1977: 164–167; Starostin, Dybo & Mudrak 2003: 1389) is retained in all four subgroups and can be safely reconstructed for Proto-Tungusic. The desemanticized element **-re* is singled out in light of such cognate pronouns as Evenki *e-leː* 'here', *taː-du* 'there', Nanai *ta-wuy* 'that (one of two distal)' etc.

Middle Korean. *kɨ̀* 'that (medial)' / *tyə́* 'that (distal)' (Starostin, Dybo & Mudrak 2003: 709, 1389).

Proto-Japonic. Old Japanese seems to have had a ternary deictic system, although only the proximal degree (*ko-*) and the medial degree (*so-*) are sufficiently well attested; distal degree *ka-* is very rare, and throughout the Middle Japanese period is being gradually replaced by *a-* (Frellesvig 2010: 139–140, 246–247). However, since monovocalic *a-* is also sufficiently well attested in Ryukyuan, it seems more prudent to reconstruct the Proto-Japonic system as **kə́-* 'this' / **sə́-* 'that (medial)' / **a-* 'that (distal)'. Vovin (1999: 90) reconstructs **ká-* and **a-* as synonyms for 'that'; the limited presence of *ka-* in Japonic might, however, suggest that the latter is a sort of "portmanteau" form coined from **a-* and **kə́-* (this solution is proposed in Starostin, Dybo & Mudrak 2003: 709).

Etymological notes. Turkic **ti*, Mongolic **te-*, Tungusic **ta-re*, Korean *tyə́* < Proto-Altaic **tʰà* (Starostin, Dybo & Mudrak 2003: 1389), correspondences seem regular. Robbeets does not consider this etymology. Additionally, Japonic **(k)á-* 'that (distal)' formally corresponds to Korean *kɨ̀* 'that (medial)', but see above on a possible secondary origin for Old Japanese *ká-*.

86. 'this'

Proto-Turkic. **bu* (Sevortyan et al. 1974–: vol. 2: 225; Dybo 2013: 500) or **kö* (Dybo 2013: 501), see notes on 'that'.

Proto-Mongolic. **e-*, see notes on 'that'.

Proto-Tungusic. **e-re*, see notes on 'that'.

Middle Korean. *í* (Starostin, Dybo & Mudrak 2003: 577).

Proto-Japonic. **kə́-* (Starostin, Dybo & Mudrak 2003: 709), **kó-* (Vovin 1999: 90). Attested in Japanese and Ryukyuan.

Etymological notes. Turkic **kö* 'this', Japonic **kə́-* 'this', further Korean *kɨ̀* 'that (medial)' < Proto-Altaic **ko* (Starostin, Dybo & Mudrak 2003: 709; Robbeets & Bouckaert 2018). Mongolic **e-*, Tungusic **e-re* < Proto-Altaic **é* (Starostin, Dybo & Mudrak 2003: 487; Robbeets & Bouckaert 2018). Correspondences seem regular.

87. 'thou'

Proto-Turkic. nom. **si*, obl. **se-n* (Sevortyan et al. 1974–: vol. 7: 232; Dybo 2013: 503); this paradigm was retained in Bulghar, but simplified in favor of the oblique stem **sen* in Nuclear Turkic.

Proto-Mongolic. **či*, gen. **či-n-*, obl. **či-ma-* (Nugteren 2011: 300; Gruntov & Mazo 2015: 244; Poppe 1955: 213), attested in all three subgroups.

Proto-Tungusic. **si* (Tsintsius 1977: 72–73; Starostin, Dybo & Mudrak 2003: 1237), attested as a basic term in all four subgroups. Cf. the apparently cognate pronoun **suː* 'you (pl.)' (Tsintsius 1977: 115; Starostin, Dybo & Mudrak 2003: 1237).

Middle Korean. *nə̀* (Starostin, Dybo & Mudrak 2003: 959).

Proto-Japonic. The issue of reconstructing the 2nd p. pronoun in Proto-Japonic is very complicated due to a large number of competing forms with unclear functional and dialectal distribution (see detailed discussion in Vovin 2010: 63–66). In light of this complexity, we resort to formal procedure and only project onto the Proto-Japonic level the one morpheme that is attested in both Japanese and Ryukyuan, namely, Common Japonic **na* (Starostin, Dybo & Mudrak 2003: 959, Vovin 1999: 90).

Etymological notes. Turkic **si*, Tungusic **si* < Proto-Altaic **si* (Starostin, Dybo & Mudrak 2003: 1237; Robbeets & Bouckaert 2018). Korean *nə̀*, Japonic **ná* < Proto-Altaic **ná* (Starostin, Dybo & Mudrak 2003: 959; Robbeets & Bouckaert 2018). Correspondences seem regular.

88. 'tongue'

Proto-Turkic. **dilk* (Sevortyan et al. 1974–: vol. 3: 228; Tenishev 2001: 227; Dybo 2013: 504), attested as a basic term in all Turkic subgroups.

Proto-Mongolic. **kele-n* (Nugteren 2011: 409; Gruntov & Mazo 2015: 244), attested in all three subgroups. Additionally see notes on 'to say'.

Proto-Tungusic. **xilŋü* (Tsintsius 1975: 316–317; Starostin, Dybo & Mudrak 2003: 796), attested as a basic term in all four subgroups. The stem demonstrates tangled reflexes of the medial cluster, the first element **l* is reconstructed on the basis of such forms as Evenki *ilŋi*, Nanai *sirmu*, Manchu *ileŋgu*, etc. Similar (although not identical) reflexes are observed for **xulŋu-* 'navel' (Tsintsius 1977: 266, 280; Starostin, Dybo & Mudrak 2003: 818) and **palŋa-* 'palm of hand' (Tsintsius 1977: 312, 314; Starostin, Dybo & Mudrak 2003: 1121) which makes Doerfer's (1995: 257) idea about the secondary nature of *-l-* in the aforementioned forms due to contamination with **ile-* 'to lick' unnecessary.

Middle Korean. *hyə́* < Proto-Korean **hyət* (Starostin, Dybo & Mudrak 2003: 1275; Vovin 2000: 145).

Proto-Japonic. **sìtà* (Starostin, Dybo & Mudrak 2003: 1275, Vovin 1999: 90). Attested in Japanese and Ryukyuan.

Etymological notes. Mongolic **kele-n*, Tungusic **xilŋü* < Proto-Altaic **k^{h}iali* (Starostin, Dybo & Mudrak 2003: 1237). Correspondences seem regular, assuming a fossilized *ŋ*-suffix in Tungusic. Further, Korean **hyət*, Japonic **sìtà* < Proto-Altaic **si̯VtV* (differently in Starostin, Dybo & Mudrak 2003: 1275). Robbeets (2005: 304) treats all the aforementioned forms (Mongolic **kele-n*, Tungusic **xilŋü*, Korean **hyət*, Japonic **sìtà*) as cognates.

89. 'tooth'

Proto-Turkic. Topologically there are two candidates: (1) **diːʎ*, meaning 'tooth' in all Nuclear Turkic subgroups, not attested in Chuvash (Sevortyan et al. 1974–: vol. 3: 242; Tenishev 2001: 228; Dybo 2013: 506). (2) **siːʎ*, meaning 'tooth' in Chuvash and 'spit, sharp stick' in many Nuclear Turkic subgroups (Dybo 2013: 507); the root is sometimes reconstructed as **siːʎ* ~ **sɨːʎ*, but actually the only unequivocal piece of evidence for the back variant **sɨ(ː)ʎ* is the Karakhanid Uyghur form from Mahmud al-Kashgari's dictionary (11th c. AD), other numerous sources point to front **-i(ː)-* or do not distinguish between **i* and **ɨ*, thus Mahmud's *ɨ* is likely to represent an occasional irregular innovation.

Semantic development is possible in both directions 'tooth' ↔ 'sharp stick', but the key piece of evidence for the Proto-Turkic meaning of **siːʎ* is the Proto-Turkic derivatives **siːʎ-leg* 'pike perch' (Sevortyan et al. 1974–: vol. 7: 257; Dybo 2013: 507) and **siːʎ-ek* 'two-year-old lamb' (Clauson 1972: 563; Räsänen 1969: 424), both are attested in the Bulghar and Nuclear Turkic branches, literally 'toothy', since specific teeth are a distinctive trait of pike perch and two-year-old lamb is an animal with adult teeth. In some Nuclear Turkic lects secondary expressions for pike perch were introduced based on **diːʎ*, literally 'toothy fish' or simply 'toothy'; semantic derivation 'tooth' > 'two-year-old small cattle' finds parallels in Mongolian (see Sevortyan et al. 1974–: vol. 7: 257).

Both reconstructed stems, **siːʎ-leg* 'pike perch' and **siːʎ-ek* 'two-year-old lamb', require additional comments. **siːʎ-leg* is reliably attested in the Bulghar branch: modern Chuvash *šзla* 'pike perch' and Hungarian *süllő* 'pike perch', borrowed from a medieval Bulghar source (Hungarian geminated *-ll-* points to Bulghar *-ll-* < **ʎl*). In Nuclear Turkic, the inherited form is Turkmen *siːle* 'pike perch'. Other Nuclear Turkic forms with back vowels could be treated as a Chuvash loan which was firstly borrowed in Tatar (*sɨla* 'pike perch') and then spread among various Turkic lects (Sevortyan et al. 1974–: vol. 7: 257) via dialectal contacts.

Proto-Turkic **si:ʎ-ek* ‘two-year-old lamb’ has a better distribution. Its medieval Bulghar descendant is retained as a loanword in Mongolian and some Nuclear Turkic lects (Sevortyan et al. 1974–: vol. 7: 257). Nuclear Turkic **si:šek* ‘two-year-old lamb’ is well attested in Ancient Turkic sources (Räsänen 1969: 424). It is worth noting that Räsänen’s idea that Nuclear Turkic **si:šek* is a deverbative from Nuclear Turkic **si:š* ‘to swell’ (Starostin, Dybo & Mudrak 2003: 429) looks like folk etymology. Firstly, Nuclear Turkic **si:š* does not mean ‘to be fat’, but ‘to swell’ (with the derivative ‘tumour’). Secondly, fatness is not a distinctive treat of two-year-old lambs.

In the light of the above, **si:ʎ* can be safely posited as a Proto-Turkic term for ‘tooth’, whereas the original meaning of **di:ʎ* remains unclear.

Proto-Mongolic. **sidü-n* (Nugteren 2011: 494; Gruntov & Mazo 2015: 244), attested in all three subgroups. Theoretically can be treated as **si-dü-n* with the rare desemanticized suffix **-du(-)n*, but there is no internal Mongolic evidence for such an analysis. Middle Mongolic *šilegü* ‘two-year-old lamb’, Buriat *šülge* ‘id.’ can formally go back to Proto-Mongolic **šil-egü*, lit. ‘toothy, animal with adult teeth’, but the distribution of the Mongolic lexeme is too narrow and most probably we are dealing with a loan from Turkic (Bulghar **šilleɣ(V)* ‘two-year-old lamb’, Sevortyan et al. 1974–: vol. 7: 257).

Proto-Tungusic. **xü:kte* (Tsintsius 1975: 300; Starostin, Dybo & Mudrak 2003: 815), attested as a basic term in all four subgroups.

Middle Korean. *ní* (Starostin, Dybo & Mudrak 2003: 1002).

Proto-Japonic. **pa* (Starostin, Dybo & Mudrak 2003: 1075), **pà* (Vovin 1999: 90). Attested in Japanese and Ryukyuan.

Etymological notes. In Starostin, Dybo & Mudrak 2003: 1251, Mongolic **sidü-n* is deduced from Pre-Proto-Mongolic **sil-dü-n* with the rare suffix **-du* and then compared with Turkic **si:ʎ* ‘tooth’ < Altaic **si:ʎa* ‘tooth’. Although such a comparison seems semantically likely, it faces certain difficulties since the clusters **ld, *ʎd* are retained in Proto-Mongolic (Starostin, Dybo & Mudrak 2003: 84–85). However, cf. similar cases: **hodu-n* (< **hol-du-n*?) ‘star’, **modu-n* (< **mo(r)-du-n*?) ‘tree’, **nidü-n* (< **nil-dü-n*?) ‘eye’. Robbeets does not consider this etymology.

90. ‘tree’

Proto-Turkic. **ɑ̰ɲ-gač* (Sevortyan et al. 1974–: vol. 1: 71; Tenishev 2001: 104; Dybo 2013: 509), attested as a basic term in all Turkic subgroups except for Karluk. Final **-gač* is a rare diminutive suffix (Räsänen 1957: 101; Sevortyan et al. 1974–: vol. 1: 73). The root is traditionally reconstructed as **ɨ-*, i.e., **ɨ-gač* (Tenishev 2001: 104), but daughter forms with nasal reflexes such as Yakut *mas*, Tuvan *ɨ̃yaš*, Tuba *aŋiš* speak in favor of Dybo’s (2013: 509) reconstruction **ɑ̰ɲ-gač.*

Proto-Mongolic. **modu-n* (Nugteren 2011: 444; Gruntov & Mazo 2015: 244), attested as a basic term in all three subgroups. In Starostin, Dybo & Mudrak 2003: 956 analyzed as **mo-du-n* with the rare desemanticized suffix **-du(-)n*, although there is no internal Mongolic evidence for such an analysis.

Proto-Tungusic. **mo:* (Tsintsius 1975: 540–541; Starostin, Dybo & Mudrak 2003: 956), attested as a basic term in all four subgroups.

Middle Korean. *nàmk ~ nàmò* (Starostin, Dybo & Mudrak 2003: 1004).

Proto-Japonic. **kə̀i* (Starostin 1991: 114), **kò-* (Vovin 1999: 90). Attested in Japanese and Ryukyuan and safely reconstructible for Proto-Japonic despite the possibility of a suspected Austronesian origin (Starostin 1991: 114).

Etymological notes. *Mongolic *modu-n* can be analyzed as **mo-du-n* (with the nominal suffix attested in some other Mongolic stems) and then plausibly compared with Tungusic **mo:* 'tree' < Altaic **mo:*. In Starostin, Dybo & Mudrak 2003: 956, Mongolic **modu-n* is deduced from Pre-Proto-Mongolic **mor-du-n* and compared with Tungusic **mo:* 'tree', Korean **mòró* 'forest', Japanese **mə̀rí* 'forest' < Altaic **mú:ro*. Such an analysis is acceptable for Tungusic **mo:* since **r* can be dropped after a long vowel in Tungusic (Starostin, Dybo & Mudrak 2003: 54), but faces certain difficulties for Mongolic since the cluster **-rd-* is not prohibited in Proto-Mongolic. At the current stage of research it would be probably better to keep the Mongolic-Tungusic 'tree' apart from the Korean-Japanese 'forest'. Cf. the similar Mongolic cases: **hodu-n* (< **hol-du-n*?) 'star', **nidü-n* (< **nil-dü-n*?) 'eye', **sidü-n* (< **sil-dü-n*?) 'tooth'. Robbeets (Robbeets & Bouckaert 2018) accepts the whole Altaic etymology.

91. 'two'

Proto-Turkic. **ẹyki* (Sevortyan et al. 1974–: vol. 1: 337; Dybo 2013: 511), attested as a basic term in all Turkic subgroups.

Proto-Mongolic. **koyar* (Nugteren 2011: 421; Gruntov & Mazo 2015: 245), attested as a basic term in all three subgroups. In some languages phonetically contaminated with **gur-ban* 'three'.

Proto-Tungusic. **ǯuwer* (Tsintsius 1975: 276–277; Starostin, Dybo & Mudrak 2003: 1374), attested as a basic term in all four subgroups. Reconstructed as **ǯube-(r)* by Starostin, Dybo & Mudrak 2003: 1374, but it seems more reasonable to treat the medial consonant as a rare occurrence of the glide **w*, cf. similarly **a:w-* 'to sleep' (q.v.) instead of **a:b-*. Ryzhkov-Shukumine (2020: 86) simply reconstructs it as **ǯüär*.

Middle Korean. *tŭ:* (Starostin, Dybo & Mudrak 2003: 1374).

Proto-Japonic. **puta* (Starostin, Dybo & Mudrak 2003: 1107, Vovin 1999: 90). Attested in Japanese and Ryukyuan.

Etymological notes. Tungusic **ǯuwer*, Korean *tŭ:* < Proto-Altaic **ti̯ubu* (Starostin, Dybo & Mudrak 2003: 1374). Correspondences seem regular, assuming a fossilized *r*-suffix in Tungusic. Robbeets does not consider this etymology.

92. 'to go'

Proto-Turkic. **bar* (Sevortyan et al. 1974–: vol. 2: 64; Dybo 2013: 513), attested as a basic term in Chuvash and many if not all Nuclear Turkic subgroups.

Proto-Mongolic. **yabu-* (Nugteren 2011: 543; Gruntov & Mazo 2015: 245), attested as a basic term in all three subgroups.

Proto-Tungusic. **ŋene-* (Tsintsius 1975: 669–671; Kazama 2003: 107; Starostin, Dybo & Mudrak 2003: 1027), attested as a basic term in all four subgroups. Distinct from **peli-* ~ **puli-* 'to walk' (Tsintsius 1977: 363–364; Starostin, Dybo & Mudrak 2003: 1133) which is also a stable concept in Tungusic.

Middle Korean. *ká-* (Starostin, Dybo & Mudrak 2003: 538), *nyə́-* (Starostin, Dybo & Mudrak 2003: 1014).

Proto-Japonic. **dúk-* (**yúk-*) (Starostin, Dybo & Mudrak 2003: 1536), **dik-* (Vovin 1999: 88). Attested in Japanese proper. Vocalism fluctuations between dialects are somewhat irregular, but the original consonantal shape is clear enough.

Etymological notes. Tungusic **ŋene-*, Korean *nyə́-* < Proto-Altaic **ŋeynV-*, correspondences seem regular. Different etymologies are proposed for the Tungusic and Korean forms in Starostin, Dybo & Mudrak 2003: 1014, 1027. Robbeets does not consider this etymology.

93. 'warm'

Proto-Turkic. Since the lexical opposition between 'warm' and 'hot' is common for the attested Turkic languages, it is natural to attempt to reconstruct the same opposition for Proto-Turkic. Normally adjectives 'warm' and 'hot' in the Turkic languages represent derivatives from verbs 'to be warm', 'to be hot', 'to burn' etc. Among them, the following candidates should be discussed.

(1) **yɨlɨ-g* (Sevortyan et al. 1974–: vol. 4: 275; Tenishev 2001: 21; Dybo 2013: 517), meaning 'warm' in all Nuclear Turkic subgroups. It is a regular deverbative from **yɨlɨ* 'to be warm' (Sevortyan et al. 1974–: vol. 4: 275; Tenishev 2001: 21) also attested in Nuclear Turkic only.

(2) **isi-g* ~ **ɨsɨ-g* (Sevortyan et al. 1974–: vol. 1: 668; Tenishev 2001: 19; Dybo 2013: 518), meaning 'warm' in Chuvash and 'hot' or 'warm' in Nuclear Turkic. It is a regular deverbative from **isi* ~ **ɨsɨ* 'to be warm' or 'to be hot' (Sevortyan et al. 1974–: vol. 1: 669) which is attested in Nuclear Turkic only.

(3) Chuvash *vəri* 'hot' which could go back to something like **ör-gey* (see Räsänen 1957: 124 for this deverbal suffix). The assumed verb **ör* is not attested in the Turkic languages, but Turkic **ört* 'steppe fire; flame' (Sevortyan et al. 1974–: vol. 1: 550) can be analyzed as **ör-t* with the deverbal suffix **-t* 'result of action' (Räsänen 1957: 142). This implies that the Proto-Turkic meaning of **ör* should be 'to burn (vel sim.)' and accordingly the meaning 'hot' of Chuvash *vər-i* has a good chance to be an inner Chuvash development.

It is thus likely that **yɨlɨ-g* and **isi-g* ~ **ɨsɨ-g* can be reconstructed for the Proto-Turkic meanings 'warm' and 'hot', but since fluctuations between these meanings are very frequent cross-linguistically, it is hard to decide which stem denoted 'warm' and which one meant 'hot'. We take **yɨlɨ-g* and **isi-g* ~ **ɨsɨ-g* as technical synonyms for 'warm'.

Proto-Mongolic. There are two Mongolic terms for 'warm', both are stably used in all three subgroups. (1) **büli-hen* (Nugteren 2011: 294; Gruntov & Mazo 2015: 246), its reflexes are usually glossed as 'warm (of liquid)' in synchronic dictionaries. (2) **dula-han* (Nugteren 2011: 319; Gruntov & Mazo 2015: 246), its reflexes are usually glossed as 'warm (not of liquid)' or specifically 'warm (of weather or clothes)' in synchronic dictionaries. Unfortunately, collocations with 'warm' applied to physical objects other than liquids are poorly documented for the Mongolic languages, but the available data show different combinatory peculiarities of **büli-hen* and **dula-han* in individual languages. E.g., in Khalkha, Oirat, Ordos, **büli-hen* 'warm' is applicable to liquids (e.g., water), breath, wind, weather, **dula-han* is applicable to weather, clothes. A Google search suggests that **büli-hen* and **dula-han* are actually interchangeable in Khalkha: instances with **büli-hen* + water or **büli-hen* + stone are more numerous than those with **dula-han*, but instances with **dula-han* + bread are more numerous than those with **büli-hen*. In other languages, e.g., Buriat and Kalmyk, **büli-hen* indeed seems to be restricted to liquids (although the available instances are scant). Since the lexical opposition 'warm (of objects)' / 'warm (of weather, clothes)' seems the most normal cross-linguistically it is likely that the Proto-Mongolic system is to be reconstructed as **büli-hen* 'warm (of objects)' / **dula-han* 'warm (of weather, clothes)'. In the attested languages, the opposition tends to diffuse, sometimes in favor of **dula-han* (in this case **büli-hen* can indeed be retained for liquids, since collocations 'warm' + a liquid are more frequent in everyday speech than those with 'warm' and non-liquid physical objects), but in other languages, on the contrary, **büli-hen* can be expanded to weather. In any case, the usual gloss 'warm (of liquid)' for the reflexes of **büli-hen* in modern dictionaries seems to be just a lexicographic imperfection of Mongolian linguistics. The two terms for 'warm' discussed above are distinct from **kala-hun* 'hot (generic application)' (Nugteren 2011: 402; Gruntov & Mazo 2015: 246).

Proto-Tungusic. Inherited words for 'warm' and 'hot' were superseded with Mongolian loans in Manchu-Jurchen. In non-Manchu-Jurchen subgroups, there are two candidates for 'warm'. The first one is **ɲama* 'warm' (Tsintsius 1975: 630–631; Starostin, Dybo & Mudrak 2003: 992). It is generally applied in Evenic (Even, Evenki, Negidal), but has restricted usage in Udiheic and Nanaic. Details are not entirely clear due to insufficiency of lexicographic descriptions available, but Udihe *ɲama-hi* 'warm' is applicable specifically to weather; likewise, Oroch *ɲama-(si)* is probably applicable to weather only (Udihe and Oroch words for 'warm (of objects)' represent various innovations). Similarly in Nanaic: Nanai *ɲama* 'warm (of weather)', likewise Ulch and Orok *ɲama* is probably applicable to weather only, at least not to liquids. No traces of **ɲama* in Manchu-Jurchen (this proto-root is to be distinguished from **ɲemu* 'soft', Tsintsius 1975: 652–653). The second candidate is **xuldü* (Tsintsius 1977: 260; Starostin, Dybo & Mudrak 2003: 849), attested as 'warm' in Nanaic: applicable to objects in Nanai, specifically to liquid in Orok and probably to objects or at least to liquid in Ulch. In other subgroups, the cognate verbs or substantives survived: Evenki *uldi* 'flame', Udihe *ugdi-* 'to heat (intr., tr.)', Manchu *wenǯe-* 'to warm up, heat (intr.)', if the Manchu word is not a Chinese loan(!) (Tsintsius 1975: 132). Various scenarios are possible. (1) **ɲama* 'warm' had general application in Proto-Tungusic (as retained in Evenic and narrowed to weather in Udiheic and Nanaic). (2) **ɲama* meant 'warm (of weather)' as opposed to **xuldü* 'warm (of objects)', this opposition is only retained in Nanaic. (3) Finally, **ɲama* could be an old term for 'warm (of weather)', the adjectival function 'warm (of objects)' is a Nanaic innovation for **xuldü*, whereas the original expression for 'warm (of objects)' has not survived in attested Tungusic. Since Evenki *uldi* 'flame' is easily explained as substantivization and the Udihe and Manchu reflexes of **xuldü* retain the meaning 'to become warm (of objects)' at least in predicative function, we provisionally fill the slot with **xuldü*. Distinct from the more

stable concept **peku* ‘hot’ (Tsintsius 1977: 322, 362; Kazama 2003: 120; Starostin, Dybo & Mudrak 2003: 1084).

Middle Korean. *tʌ̀sʌ̀-* (Starostin, Dybo & Mudrak 2003: 1067).

Proto-Japonic. **àtà-taka-* (Starostin, Dybo & Mudrak 2003: 1067, Vovin 1999: 90). Attested in Japanese proper.

94. ‘water’

Proto-Turkic. **sɨb* (Sevortyan et al. 1974–: vol. 7: 325; Tenishev 2001: 88; Dybo 2013: 519), attested as a basic term in all Turkic subgroups.

Proto-Mongolic. **usu-n* (Nugteren 2011: 535; Gruntov & Mazo 2015: 246), attested as a basic term in all three subgroups.

Proto-Tungusic. **mu:* (Tsintsius 1975: 548–549; Kazama 2003: 42; Starostin, Dybo & Mudrak 2003: 935), attested as a basic term in all four subgroups.

Middle Korean. *mɨ́r* (Starostin, Dybo & Mudrak 2003: 935).

Proto-Japonic. **mí-* (Starostin, Dybo & Mudrak 2003: 935, Vovin 1999: 90). Attested in Japanese and Ryukyuan.

Etymological notes. In Starostin, Dybo & Mudrak 2003: 1285, Mongolic **usu-* is explained as Pre-Mongolic **sub-su-* with a fossilized suffix *-*su* > **su-su-* > with dissimilation **u-su-*. Such a complicated scenario is theoretically possible and would allow us to connect Turkic **sɨb* and Mongolic **usu-*, but there are too many unprovable assumptions to regard this as a safe etymology, therefore we keep these forms apart. Additionally,Tungusic **mu:*, Korean *mɨ́r*, Japonic **mí-* < Proto-Altaic **mi̯ù:ri* (Starostin, Dybo & Mudrak 2003: 935); Robbeets (Robbeets & Bouckaert 2018) accepts the Tungusic-Korean comparison without the Japonic *comparandum*.

95. ‘we’

Proto-Turkic. **bi-rʸ* (Sevortyan et al. 1974–: vol. 7: 232; Dybo 2013: 503), attested in all subgroups. No clusivity. Morphologically a plural form of **bi* ‘I’ q.v.

Proto-Mongolic. Not all languages retain the clusivity opposition, but nevertheless two separate pronominal paradigms can be safely reconstructed for Proto-Mongolian: **ba*, obl. **ma-n-* (< **ba-n-*) ‘we (exclusive)’ (Nugteren 2011: 276; Gruntov & Mazo 2015: 246; Poppe 1955: 215), **bi-da*, obl. **bida-n-* ‘we (inclusive)’ (Nugteren 2011: 281; Gruntov & Mazo 2015: 246; Poppe 1955: 215). Both pronouns can hardly be disconnected from **bi*, gen. **mi-n* (< **bi-n*) ‘I’, but morphological details are unclear.

Proto-Tungusic. The clusivity opposition is attested in Evenic, Udiheic and Manchu and thus should be reconstructed for Proto-Tungusic: **bu:* ~ **bue*, obl. **mu-n-* (< **bu-n-*) ‘we (exclusive)’ (Tsintsius 1975:

98; Starostin, Dybo & Mudrak 2003: 341), *mu-n-ti* (< obl. *mu-n-* with unclear *-*ti*) 'we (inclusive)' (Tsintsius 1975: 539; Starostin, Dybo & Mudrak 2003: 341). In Nanaic, the clusivity opposition was simplified in favor of exclusive *bu:*. Manchu *muse* 'we (incl.)' - a new compound of obl. *mu-* 'we (excl.)' + *si* 'thou'. In sum, the Tungusic clusivity opposition looks like an innovation, which probably took place already in Proto-Tungusic. Cognate to *bi*, obl. *mi-n-* (< *bi-n-*) 'I', but morphological details are unclear.

Middle Korean. *úrí* (Starostin, Dybo & Mudrak 2003: 341).

Proto-Japonic. *bà-* (Starostin, Dybo & Mudrak 2003: 341), *bàn[u]* (Vovin 1999: 90). Attested in Japanese and Ryukyuan. No clusivity; same root as in 'I' q.v.

Etymological notes. The plural pronoun 'we' is normally derived from the singular pronoun 'I', but in all likelihood independently in Turkic, Mongolic and Tungusic (Starostin, Dybo & Mudrak 2003: 341). Especially note Turkic *bi-r^y* 'we', a regular plural of Turkic *bi* 'I'. Korean *úrí* might represent Pre-Proto-Korean *bu-ri* with irregular loss of *b-* and the same plural exponent as in the Turkic form.

96. 'what?'

Proto-Turkic. *ne:* (Sevortyan et al. 1974–: vol. 7: 96; Dybo 2013: 522), attested in all subgroups with the probable exception of Yakut and Tofa-Tuvinian (otherwise a quite irregular *y*-like development of *n-* should be assumed in these subgroups). Many languages, including Chuvash, reflect the stem modified with an additional morpheme: *ne:-me*.

Proto-Mongolic. *yahun* ~ *yahan* (Nugteren 2011: 543; Gruntov & Mazo 2015: 247; Poppe 1955: 229), attested as a basic term in all three subgroups.

Proto-Tungusic. *xia* ~ *xay* (Tsintsius 1975: 286–288; Starostin, Dybo & Mudrak 2003: 754), attested as a basic term in Evenic, Nanaic and Manchu.

Middle Korean. *mɨ̀sɨ́* (Starostin, Dybo & Mudrak 2003: 958).

Proto-Japonic. *nV̀-* (Starostin, Dybo & Mudrak 2003: 1034), *nà[-]ní* (Vovin 1999: 90). Attested in Japanese and Ryukyuan (although the vocalism in Proto-Ryukyuan *nu*, as opposed to Old Japanese *na-ni*, is difficult to explain).

Etymological notes. In Starostin, Dybo & Mudrak 2003: 1034, Turkic *ne:* (probably a unique occurrence of an initial nasal in Proto-Turkic), Mongolic *yahun* and Japonic *nà-* are traced back to Altaic *ŋi̯V-*. Such a solution is possible (assuming an unusual reflex of Altaic *ŋ-* in Turkic and a fossilized morpheme *-*hun* in Mongolic), but not obligatory. We assign the same cognation index to Turkic *ne:* and Japonic *nà-* while keeping Mongolic *yahun* apart. Robbeets does not consider this etymology.

97. 'white'

Proto-Turkic. A very unclear situation with two candidates in Nuclear Turkic. (1) *a:k* (Sevortyan et al. 1974–: vol. 1: 116; Tenishev 2001: 598; Dybo 2013: 523; Clauson 1972: 75), attested as a basic term for

‘white’ in all Nuclear Turkic subgroups (including some Ancient Turkic sources) except for Yakut; this root is simply not attested in Chuvash and Yakut. (2) **hürü-ŋ* (Tenishev 2001: 601; Dybo 2013: 524; Clauson 1972: 233), meaning ‘white’ in Yakut, Khalaj and many Ancient Turkic sources (not attested outside these languages). It is possible that one of them (**hürü-ŋ*?) originally meant ‘white’, another meant ‘pale’ or ‘bright’ (**aːk*?), or both meant ‘white’, but **hürü-ŋ* was a generic term, whereas **aːk* expressed a color of horses and cattle. Details are not clear, however, so we accept **aːk* and **hürü-ŋ* as synonyms. In Chuvash, the old term for ‘white’ was recently superseded with **siaːrɨg* ‘yellow’ q.v.

Proto-Mongolic. **čaga-han* (Nugteren 2011: 298; Gruntov & Mazo 2015: 247), attested as a basic term in all three subgroups. A derivative from **čahi-* ‘to be(come) white’ (Nugteren 2011: 298), i.e., **čaga-han* < Pre-Proto-Mongolic **čaha-han* with the regular dissimilation *h-h* > *g-h*.

Proto-Tungusic. **šaː-* (Tsintsius 1977: 380–382; Kazama 2003: 125; Starostin, Dybo & Mudrak 2003: 1322, 1324), this *CV*-root, modified with various suffixes, forms basic terms for ‘white’ in all subgroups except for Evenic. The most transparent structure with a common suffix is **šaː-gǯa* attested in Nanaic: Nanai *čaːgǯa-n*, Ulch *ča(ː)gǯa-n*, Orok *ta(ː)gda* ‘white’ (no reasons to treat these as Mongolic loans). Other stems demonstrate several rare and fossilized suffixes: Udihe *ča-ligi* ‘white’, Oroch *čeː-kke* ‘white’; additionally, the stem **čaː-m* ‘white’ occurs in Udiheic and Nanaic, but not as a basic term. The stems **šaː-ŋgia-(n)* is used in Manchu-Jurchen, although the Manchu-Jurchen data represent a somewhat special case: Jurchen *šaŋgia*, Manchu *šaŋgʲan* (with the irregular abbreviated variant *šanyan*), Xibe *šʲaŋã*, all with polysemy ‘smoke / white’. These Manchu-Jurchen forms are derivable from both Proto-Tungusic **saŋŋa-n* ‘smoke’ q.v. and **šaː-ŋgia-(n)* ‘white’, so we are probably dealing with secondary polysemy here. Cf. the same suffix patterns in the stems for ‘green, blue’ q.v.: **ɲog-(g)ǯo* in Nanaic and Udiheic, **ɲog-aŋgia-(n)* in Manchu-Jurchen. In Evenic, ‘white’ is expressed with the help of either new formations from **bag-* ‘to become clear (of sky)’ (**bag-d-* ‘white’ in Evenki, Negidal) or etymologically isolated forms. It is proposed in Starostin, Dybo & Mudrak 2003: 1322, 1324 to divide the aforementioned Tungusic forms for ‘white’ into two separate proto-roots: **šaːŋ-* and **šaːk-*. However, such a solution does not explain the observed peculiarities, and does not seem justified, cf. some other color terms, e.g., ‘red’, ‘yellow’, which also demonstrate the abundance of petrified suffixes (including **-l-, *-m-* as in ‘white’) distributed among individual languages.

Middle Korean. *hʌ́y-* (Starostin, Dybo & Mudrak 2003: 1264).

Proto-Japonic. **sìruà-* (Starostin, Dybo & Mudrak 2003: 1264), **sírà-Cu* (Vovin 1999: 90). Attested in Japanese and Ryukyuan.

Etymological notes. Mongolic **čaga-* and Tungusic **šaː-* could be connected under the assumption of a fossilized *ga*-suffix in the Mongolic form < Altaic **šàː-* (cf. Starostin, Dybo & Mudrak 2003: 1322, 1324 with an incorrect analysis of the Tungusic data). Nevertheless it seems more prudent to keep these forms apart. Korean *hʌ́y-*, Japonic **sìruà-* < Proto-Altaic **si̯àːyri* (Starostin, Dybo & Mudrak 2003: 1264). Correspondences seem regular. Robbeets (2015: 112; Robbeets & Bouckaert 2018) rejects the Korean-Japonic etymology.

98. ‘who?’

Proto-Turkic. **kim* ~ **kem* (Sevortyan et al. 1974–: vol. 5: 67; Dybo 2013: 525), attested in all subgroups.

Proto-Mongolic. **ke-* (Nugteren 2011: 410; Gruntov & Mazo 2015: 247; Poppe 1955: 229), attested as a basic term in all three subgroups. Paradigm: sg. **ke-n* / pl. **ke-d*.

Proto-Tungusic. **ŋü:* (Tsintsius 1975: 660–661; Starostin, Dybo & Mudrak 2003: 1034), attested as a basic term in all four subgroups.

Middle Korean. *nú* (Starostin, Dybo & Mudrak 2003: 1034).

Proto-Japonic. **tá* (Tower of Babel project; Vovin 1999: 90). Attested in Japanese and Ryukyuan.

Etymological notes. Turkic **kim*, Mongolic **ke-* < Proto-Altaic **kʰe* (Starostin, Dybo & Mudrak 2003: 754). Correspondences seem regular, assuming a fossilized *m*-suffix in Turkic (such an affixation of *CV*-pronouns is frequent cross-linguistically so it does not seem risky to compare these forms). Tungusic **ŋü:*, Korean *nú* < Proto-Altaic **ŋi̯V* (Starostin, Dybo & Mudrak 2003: 1034). Robbeets does not consider these etymologies.

99. 'woman'

Proto-Turkic. A highly unstable item, which cannot be reconstructed with certainty. See Dybo 2013: 527–530 for overview.

Proto-Mongolic. **eme* (Nugteren 2011: 328; Gruntov & Mazo 2015: 247), the root is attested in all three subgroups. Apparently to be reconstructed with polysemy 'woman / female (of animals)'. In some modern languages, the bare root **eme* is retained with the meaning 'female', whereas the meaning 'woman' is expressed with the help of suffixed stems. Cf. the same situation with **ere* 'man; male' q.v.

Proto-Tungusic. The most probable candidate is **asi:* (Tsintsius 1975: 55; Starostin, Dybo & Mudrak 2003: 271), attested as a basic term frequently with polysemy 'woman / wife' (see Kazama 2003: 59 for 'wife') in Evenic, Udiheic and Nanaic ('woman, wife' in Orok, narrowed in 'wife' in Nanai, Ulch). Its Manchu cognate means 'elder brother's wife'. Distinct from **eke* (Tsintsius 1977: 443; Starostin, Dybo & Mudrak 2003: 499), which became a basic term for 'woman' in some Nanaic lects (Nanaic, Ulch) and a secondary synonym for 'woman' in Orok and Oroch, but means 'elder sister' in Evenic and 'female relative' in Udihe. The second candidate is **keke* (Tsintsius 1975: 480; Starostin, Dybo & Mudrak 2003: 499), a basic term for 'woman' in Manchu, Xibe (borrowed as 'wife' in Solon) and 'button loop' in Nanaic. If Manchu-Jurchen is indeed the first outlier, **asi:* and **keke* are equiprobable candidates, so we take them as technical synonyms. In Starostin, Dybo & Mudrak 2003: 499, two proto-roots **eke* and **keke* are unjustifiably merged together.

Middle Korean. *kyə̌:-čìp* (Starostin, Dybo & Mudrak 2003: 499), the second element means 'house'.

Proto-Japonic. **bəmina* (Starostin 1991: 114), **-mina* (Vovin 1999: 90). Attested in Japanese and Ryukyuan. It is sometimes thought (e.g. Martin 1987: 507; Vovin 1999; etc.) that **bə-mina* is to be analyzed as **bə* ʻman' + (unattested) **mina* ʻwoman', but since the meaning of **bə* is specifically ʻmale' (rather than

‘person’ in general), such an analysis is dubious. The form has sometimes been identified as a potential Austronesian loanword (Starostin 1991: 114).

Etymological notes. In Starostin, Dybo & Mudrak 2003: 499, Tungusic **keke* is compared with Korean *kyǝ̆ː-(čip)* < Proto-Altaic **ekʰà* which is highly dubious. Robbeets does not consider this etymology.

100. ‘yellow’

Proto-Turkic. **siaːrɨg* (Sevortyan et al. 1974–: vol. 7: 206; Tenishev 2001: 601; Dybo 2013: 531), attested as a basic term for ‘yellow’ in all Nuclear Turkic subgroups. In Chuvash, it means ‘white’, but Hungarian ⟨sárga, sárog⟩ ‘yellow’ borrowed from a Bulghar source proves that the shift ‘yellow’ > ‘white’ is a late Chuvash innovation.

Proto-Mongolic. **sira* (Nugteren 2011: 492; Gruntov & Mazo 2015: 248), attested as a basic term in all three subgroups.

Proto-Tungusic. **soː-* (Tsintsius 1977: 103–104; Starostin, Dybo & Mudrak 2003: 1308), a *CV*-root which is not attested in Evenic, but forms words for ‘yellow’ in other subgroups with the help of various suffixes: **soː-gǯo* ‘yellow’ in Udiheic and Nanaic, **soː-gia* in Manchu-Jurchen. Cf. further such cognate stems as **soː-m* ‘becoming yellow’ in Nanaic, Udihe *so-ligi* ‘blond, red (of hair)’, etc. The non-standard morphological situation is similar to that of some other color names, e.g., ‘white’ q.v. Differently in Evenic, where expressions with polysemy ‘yellow, brown, gray’ are based on the root **siŋa-* (Tsintsius 1977: 90; Starostin, Dybo & Mudrak 2003: 1268) with various suffixes. No traces of **siŋa-* outside Evenic. If Manchu-Jurchen is indeed the first outlier, **soː-* has the advantage, but we prefer to treat **soː-* and **siŋa-* as technical synonyms.

Middle Korean. *nùrɨ́-* (Starostin, Dybo & Mudrak 2003: 1032).

Proto-Japonic. **kú-i* (Starostin, Dybo & Mudrak 2003: 695), **kú-Ci* (Vovin 1999: 90). Attested in Japanese and Ryukyuan.

Etymological notes. Turkic **siaːrɨg*, Mongolic **sira*, Tungusic **soː-* < Proto-Altaic **siòːyri* (Starostin, Dybo & Mudrak 2003: 1264; Robbeets & Bouckaert 2018, both sources with another Tungusic *comparandum*). Correspondences seem regular, assuming a fossilized *g*-suffix in Turkic. The vowel match is the same as in Turkic **diaːʎ*, Mongolic **čila-hu-n*, Tungusic **ǯolo* ‘stone’ q.v. Normally Altaic **-r-* is retained in Tungusic (Starostin, Dybo & Mudrak 2003: 24), but in Starostin, Dybo & Mudrak 2003: 54, several etymologies are offered where Altaic **-r-* is lost in Tungusic after a long vowel, i.e., in the roots of the shape *CVːr-* (e.g., ‘to give’ q.v.).

101. ‘far (adv.)’

Proto-Turkic. **ɨyra-k* (Sevortyan et al. 1974–: vol. 4: 286; Dybo 2013: 534), attested as a basic term in all Nuclear Turkic subgroups as well as in Ancient Turkic sources (Clauson 1972: 214). A regular deverbative from Nuclear Turkic **ɨyra* ‘to become far, distant’ (Sevortyan et al. 1974–: vol. 4: 286). In many lects,

ɨyra-k competes with **ur*^{y}*a-k* ‘far’ (Sevortyan et al. 1974–: vol. 1: 570; Dybo 2013: 534), derived from the verb **ur*^{y}*(a)* ‘to stretch (intr.), be long’, the latter, however, has a narrower distribution and looks like an innovation for this meaning spreading via dialect contacts. A not entirely clear derivative *in-ʃe* ‘far’ in Chuvash (Dybo 2013: 536).

Proto-Mongolic. **kolo* (Nugteren 2011: 417; Gruntov & Mazo 2015: 248), an adjectival stem attested as a basic term in all three subgroups.

Proto-Tungusic. **goro* (Tsintsius 1975: 161–162; Kazama 2003: 119; Starostin, Dybo & Mudrak 2003: 573), attested as a basic term in all four subgroups.

Middle Korean. *mə̆ːr-* (Starostin, Dybo & Mudrak 2003: 897).

Proto-Japonic. **tə́pə́-* (Starostin, Dybo & Mudrak 2003: 1427). Attested in Japanese and Ryukyuan.

102. ‘heavy’

Proto-Turkic. **iagɨr* (Sevortyan et al. 1974–: vol. 1: 85; Tenishev 2001: 338; Dybo 2013: 537), attested as a basic term in all Turkic subgroups.

Proto-Mongolic. **kündü* (Nugteren 2011: 435; Gruntov & Mazo 2015: 248), attested as a basic term in all three subgroups.

Proto-Tungusic. **xurge* (Tsintsius 1977: 283–284; Starostin, Dybo & Mudrak 2003: 826), attested as a basic term in all four subgroups.

Middle Korean. *mɨ̀k-ə́β-* (Starostin, Dybo & Mudrak 2003: 295).

Proto-Japonic. **ə́m(p)ə́-* (Starostin, Dybo & Mudrak 2003: 295). Attested in Japanese and Ryukyuan (the variant *ə́mə́- is Japanese, *ə́mpə́- is Ryukyuan).

Etymological notes. In Starostin, Dybo & Mudrak 2003: 295, the Korean root is analysed as a suffixed stem *mɨ̀-k-* and compared with Japonic **ə́m(p)ə́* < Proto-Altaic **ámbe*. This would be acceptable, if we had internal Korean evidence for suffixal origin of *-k*. Robbeets does not consider this etymology.

103. ‘near (adv.)’

Proto-Turkic. **yạgu-k* (Sevortyan et al. 1974–: vol. 4: 63; Dybo 2013: 539; Clauson 1972: 901), attested as a basic term in Chuvash and some Nuclear Turkic subgroups (incl. Yakut and Tofa-Tuvinian) as well as in Ancient Turkic sources. Regular deverbative from **yạgu* ‘to approach’ (Sevortyan et al. 1974–: vol. 4: 62). Cf. a phonetically similar form **yak-ɨn* ‘near’ in some Nuclear Turkic lects (Dybo 2013: 539).

Proto-Mongolic. **ohira* (Nugteren 2011: 417; Gruntov & Mazo 2015: 248), an adjectival stem attested as a basic term in all three subgroups.

Proto-Tungusic. **daga* (Tsintsius 1975: 187–188; Kazama 2003: 119; Starostin, Dybo & Mudrak 2003: 456), attested as a basic term in Evenic, Udiheic (Udihe) and probably Jurchen. In other lects, usually superseded with forms which lack reliable etymology, e.g., Nanai, Ulch *ǯiǯa* or Manchu *xanči*.

Middle Korean. *kàs-kàβ-* < Proto-Korean **kàč-kàb-* (Starostin, Dybo & Mudrak 2003: 811).

Etymological notes. Tungusic **daga*, Japonic **tìkà-* < Proto-Altaic **tagi*, correspondences are regular except for **t-* instead of **d-* in Japonic, since it is proposed by Starostin, Dybo & Mudrak 2003: 47 that Proto-Altaic initial **t-* regularly shifts > Japonic **d-* before Japonic **i*. This is indeed so in the majority of cases, but, firstly, instances for the retention of voiceless **t-* before **i* in Japonic are also know and, secondly, one could suppose an occasional assimilative influence on the part of the medial **-k-*. Differently in EDAL, where Turkic **ya̯gu-k* 'near', Tungusic **daga* 'near', Japonic **dánká-* 'soon after, before long' < Proto-Altaic **dágá* (Starostin, Dybo & Mudrak 2003: 456), Turkic **yak-* 'to come near', Tungusic **daxa-* 'to follow', Japonic **tìkà-* 'near' < Proto-Altaic **dàk*^{h}*i* (Starostin, Dybo & Mudrak 2003: 458). Note that even Turkic **ya̯gu-k* 'near' is eventually related to Tungusic **daga* 'near', the Turkic form is an inner Turkic deverbative from Turkic **ya̯gu-* 'to approach, come near', thus at best we would be dealing with a derivational drift. Robbeets (2005: 298) units Turkic **yak*-, Tungusic **daga* ~ **daka*, Japonic **tika*-.

104. 'salt'

Proto-Turkic. **du:r*^{y} (Sevortyan et al. 1974–: vol. 3: 288; Dybo 2013: 541), attested as a basic term in all Turkic subgroups.

Proto-Mongolic. **dabu-su* (Nugteren 2011: 310; Gruntov & Mazo 2015: 249), attested as a basic term in the Northern and Southern branches. Cf. the unetymologizable Dagur form *kata:* 'salt'.

Proto-Tungusic. Not reconstructible and almost everywhere superseded with the Mongolic loan (Tsintsius 1975: 186; Kazama 2003: 26). Evenki *turuke* and Udihe *saʔi* 'salt' remain without etymology. Even *tak* 'salt' might be cognate to Manchu *takan* 'the name of an edible mustard-like wild plant that grows along streams' (Starostin, Dybo & Mudrak 2003: 1396), but the comparison seems too dubious.

Middle Korean. *sòkòm* (Starostin, Dybo & Mudrak 2003: 1204).

Proto-Japonic. **sìpə̀* (Starostin, Dybo & Mudrak 2003: 1238). Attested in Japanese and Ryukyuan.

Etymological notes. Turkic **du:r*^{y} and Mongolic **dabu-su* are sometimes thought to be cognates < Proto-Altaic **čiober*^{y}*V* (Starostin, Dybo & Mudrak 2003: 398), but even assuming the simplification of the cluster **rs* in Mongolic (which is usually retained, see Starostin, Dybo & Mudrak 2003: 83), development of the medial labial is not regular. It seems more prudent to treat these forms as unrelated. Robbeets (2005: 398) does not accept this etymology.

105. 'short'

Proto-Turkic. **kɨs-ka* (Sevortyan et al. 1974–: vol. 6: 247; Dybo 2013: 542), attested as a basic term in all Nuclear Turkic subgroups, except for Yakut, as well as in Ancient Turkic sources (Clauson 1972: 667). Derived from the verb **kɨs* 'to compress, squeeze' (Sevortyan et al. 1974–: vol. 6: 246) with the rare deverbal suffix **-ka* (Räsänen 1957: 124). Non-productivity of this suffix proves the antiquity of the stem **kɨs-ka*, although it is unknown whether 'short' was the original Proto-Turkic meaning of **kɨs-ka* or not (cf. Chuvash *xəzək* 'narrow' from the same verb, but with another suffix). The second and much more marginal candidate for Proto-Turkic 'short' is Poshkart Chuvash *mɜt-ɜk* 'short' (if inherited) < something like Proto-Turkic **mutu-k*, lacking any Turkic *comparanda*.

Proto-Mongolic. **hokar* ~ **hakor* (Nugteren 2011: 358; Gruntov & Mazo 2015: 249), attested as a basic term in all three subgroups.

Proto-Tungusic. The first candidate is **xuru-mü* (Tsintsius 1977: 287–288; Kazama 2003: 117; Starostin, Dybo & Mudrak 2003: 843), a basic term in non-Manchu-Jurchen subgroups (no traces in Manchu-Jurchen). The second candidate is **poko-lo* (Tsintsius 1977: 331; Kazama 2003: 117; Starostin, Dybo & Mudrak 2003: 1100), a basic term in Manchu-Jurchen, which might be cognate to Evenki *hoko-pčo* 'sacral bone', if the original meaning of *hoko-pčo* was 'coccyx, tailbone' < 'short (bone)' (*-pčo* is an adjectival suffix). If Manchu-Jurchen is indeed the first outlier, **xuru-mü* and **poko-lo* are equiprobable candidates, so we take them as technical synonyms.

Middle Korean. *tyə̀r-ɨ̀-* (Starostin, Dybo & Mudrak 2003: 1372).

Proto-Japonic. **mìnsì-kà-* (Starostin, Dybo & Mudrak 2003: 1010). Attested in Japanese and Ryukyuan. Final **-kà-* is a frequent adjectival suffix, although the pure stem **mìnsì-* is not encountered by itself.

Etymological notes. Mongolic **hokar* ~ **hakor*, Tungusic **poko-lo* < Proto-Altaic **pìùkʰì* (Starostin, Dybo & Mudrak 2003: 1100). Correspondences seem regular. Robbeets does not consider this etymology.

106. 'snake'

Proto-Turkic. **yɨl-an* (Sevortyan et al. 1974–: vol. 4: 277; Tenishev 2001: 180; Dybo 2013: 544), attested as a basic term in all subgroups except for Yakut. Derived from the verb **yɨl* 'to crawl' (Sevortyan et al. 1974–: vol. 4: 40).

Proto-Mongolic. **mogay* (Nugteren 2011: 445; Gruntov & Mazo 2015: 250), attested as a basic term in all three subgroups.

Proto-Tungusic. **mü:ki* (Tsintsius 1975: 537–538; Starostin, Dybo & Mudrak 2003: 932), attested as a basic term for 'snake' is all subgroups: Evenic (Lower Negidal), Udiheic (Oroch), Nanaic (everywhere; polysemy 'snake; worm' in Orok), Manchu-Jurchen. Occasionally gets contaminated or phonetically coincides with **mirkü-* 'to crawl' (Tsintsius 1975: 537–538; Starostin, Dybo & Mudrak 2003: 926).

Middle Korean. *pʌ́yám* (Starostin, Dybo & Mudrak 2003: 1165).

Proto-Japonic. **pàim(p)V* (Starostin, Dybo & Mudrak 2003: 1165). Attested in Japanese and Ryukyuan. Vovin (2010: 104) claims that Ryukyuan *pabu is not related to Old Japanese pemyi, but Thorpe (1983:

332) reconstructs Proto-Ryukyuan variation *pebu ~ *pabu and suggests that *-bu may be "a suffix of animates". If so, the Ryukyuan forms may actually represent a contraction from something like *pàimi-mpu, which would explain the discrepancy in vocalism.

Etymological notes. Mongolic **mogay*, Tungusic **mü:ki* < Proto-Altaic **miu:ko* (Starostin, Dybo & Mudrak 2003: 932). Korean *pʌ́yám*, Japonic **pàimV* ~ **pàimpV* < Proto-Altaic **pʰòyamV* (Starostin, Dybo & Mudrak 2003: 1165). Correspondences seem regular. Robbeets (2005: 206) treats the Japonic forms as a Korean loan; such a scenario cannot be ruled out, but does not have any advantage over the idea of a common genetic origin for both forms.

107. 'thin'

Proto-Turkic. The lexical opposition between 'thin 2D' and 'thin 1D' is typical for Turkic languages and can be reconstructed for Proto-Turkic. (1) **yubka* 'thin 2D' (Sevortyan et al. 1974–: vol. 4: 241; Dybo 2013: 547), attested as a basic term in Chuvash and the majority of Nuclear Turkic subgroups. (2) **yiŋč-ge* 'thin 1D' (Sevortyan et al. 1974–: vol. 1: 364; Dybo 2013: 546), attested as a basic term in all Turkic subgroups.

Proto-Mongolic. The lexical opposition between 'thin 2D' and 'thin 1D' is typical for Mongolic languages and can be safely reconstructed for Proto-Mongolic, both terms are very stable and attested in all three subgroups. (1) **nim-gen* 'thin 2D' (Nugteren 2011: 460; Gruntov & Mazo 2015: 250). (1) **nari-n* 'thin 1D' (Nugteren 2011: 452; Gruntov & Mazo 2015: 250).

Proto-Tungusic. **nem-* (Tsintsius 1975: 621; Starostin, Dybo & Mudrak 2003: 989), attested as a basic term in all four subgroups. In Evenic and Manchu-Jurchen modified with the suffix **-ku-n*. No lexical opposition between 2D and 1D.

Middle Korean. *yə̀rβ-* '2D' < Proto-Korean **yə̀rb-* (Starostin, Dybo & Mudrak 2003: 972), *kʌ́nʌ́r-* '1D' (Starostin, Dybo & Mudrak 2003: 776).

Proto-Japonic. **úsú-* 'thin 2D' (Starostin, Dybo & Mudrak 2003: 1035); **pə̀sə̀-* (Japanese) ~ **pìsà-* (Ryukyuan) 'thin 1D' (Starostin, Dybo & Mudrak 2003: 1079).

Etymological notes. Mongolic **nim-gen*, Tungusic **nem-* < Proto-Altaic **nemi* (slightly differently in Starostin, Dybo & Mudrak 2003: 989,). Correspondences seem regular. Korean **yə̀rb-* '2D' is tentatively connected to Mongolic **nari-n* 'thin 1D' in Starostin, Dybo & Mudrak 2003: 972, but since the onset correspondence is irregular, we prefer to reject this etymology. Robbeets (Robbeets & Bouckaert 2018) does not accept these etymologies.

108. 'wind'

Proto-Turkic. **yẹl* (Sevortyan et al. 1974–: vol. 4: 174; Tenishev 2001: 40; Dybo 2013: 549), attested as a basic term in all subgroups except for Yakut. (Sevortyan et al. 1974–: vol. 4: 174; Tenishev 2001: 40; Dybo 2013: 549).

Proto-Mongolic. The main candidate is **key* (Nugteren 2011: 409; Gruntov & Mazo 2015: 250), attested as the basic term 'wind' in the Southern branch (incl. Middle Mongolic) and Dagur and with the meaning 'air, atmosphere' in the Northern branch, where it may have been superseded in the meaning 'wind' with **salki-n* (Nugteren 2011: 481; Gruntov & Mazo 2015: 250), which means 'breeze, soft wind' in some Southern lects (thus 'breeze' could be a Proto-Mongolic meaning of **salki-n*). Mongolic **key* resembles Middle Chinese 氣 **kʰɨy* 'air, vapour, qi' and is sometimes regarded as a Chinese loanword which is not very likely since **key* seems too deeply rooted in Mongolic, but cannot be fully excluded. Because of this we treat **key* and **salki-n* as technical synonyms.

Proto-Tungusic. **xedü-n* (Tsintsius 1977: 438–439; Starostin, Dybo & Mudrak 2003: 771), attested as a basic term in all four subgroups. The cognate verb **xedü-* 'to blow' is attested in Manchu. In other subgroups, the verb 'to blow' represents a secondary formation: **xedü-l-* in Evenic, denominative **xedün(V)-* in Udiheic and Nanaic.

Middle Korean. *pʌ̀rʌ̀m* (Starostin, Dybo & Mudrak 2003: 375).

Proto-Japonic. **kánsá-i* (Starostin, Dybo & Mudrak 2003: 642). Attested in Japanese and Ryukyuan.

Etymological notes. Turkic **yẹl*, Mongolic **salki-n* < Proto-Altaic **zali* (Starostin, Dybo & Mudrak 2003: 1508). Correspondences seem regular, assuming a fossilized *k*-suffix in Mongolic. Robbeets does not consider this etymology.

109. 'worm'

Proto-Turkic. The main candidate is **kuːrt* (Sevortyan et al. 1974–: vol. 6: 167; Tenishev 2001: 181; Dybo 2013: 552), meaning 'worm (in general)' in Nuclear Turkic and generally 'insect (esp. bee), worm, larva, caterpillar' in Chuvash. Cf. also **uman*, reconstructed on the basis of Chuvash *əман* 'earthworm' (Dybo 2013: 553).

Proto-Mongolic. **kora-kay* (Nugteren 2011: 420; Gruntov & Mazo 2015: 250), a very stable word with polysemy 'worm (in general) / insect (in general)'. Final **-kay* is a nominal suffix probably with diminutive semantics.

Proto-Tungusic. A relatively unstable item. An additional difficulty is that in non-Manchu-Jurchen subgroups three concepts are often lexically discriminated: 'worm' in general (it can also cover insects *per se* and even reptiles), 'larva in rotten meat' (the so-called "yukola worm"), and 'earthworm'. The main candidate for 'worm (in general)' is **kuliː* or **kuli-kaː-n* with a diminutive *k*-suffix (Tsintsius 1975: 428; Kazama 2003: 34; Starostin, Dybo & Mudrak 2003: 736), which is attested as a generic term for 'worm' in Evenic (Negidal, Evenki, in the latter language the plain stem acquired the meaning 'snake', whereas the suffixed one means 'worm; insect'), Udiheic (Udihe 'snake; worm; insect; reptile', Oroch 'worm; caterpillar; snake'), Nanaic (Nanai 'worm; insect', Orok 'worm; insect', Ulch 'worm'). No traces of **kuliː* in Manchu-Jurchen. The second candidate is **xuŋVl* (Tsintsius 1977: 280; Starostin, Dybo & Mudrak 2003: 823), this is a basic general term for 'worm; insect' in Manchu and for 'worm' in Jurchen (this root is modified with the suffix **-ka* in Manchu-Jurchen), but for other subgroups **xuŋVl* can be safely reconstructed with the

specific meaning 'larva in rotten meat (e.g., yukola worm)'. If Manchu-Jurchen is indeed the first outlier, **kuli:* and **xuŋVl* are equiprobable candidates, so we take them as technical synonyms.

Middle Korean. *pə̀r-ʔə́y* (Starostin, Dybo & Mudrak 2003: 1151).

Proto-Japonic. **músí* (Starostin, Dybo & Mudrak 2003: 893). Attested in Japanese and Ryukyuan.

Etymological notes. Turkic **ku:rt*, Mongolic **kora-kay* < Proto-Altaic **kʰió:ro* (Starostin, Dybo & Mudrak 2003: 807). Correspondences seem regular, assuming a fossilized *k*-suffix in Turkic. Robbeets does not consider this etymology.

110. 'year'

Proto-Turkic. For Nuclear Turkic, the following lexical opposition can be safely reconstructed: **yɨl* 'year (as a time period)' (Sevortyan et al. 1974–: vol. 4: 275; Tenishev 2001: 70; Dybo 2013: 554) vs. **ya:ʎ* 'year (as a unit of measure for age)' (Sevortyan et al. 1974–: vol. 4: 162; Tenishev 2001: 84; Dybo 2013: 555). In Chuvash, only **ya:ʎ* is attested meaning 'year (in general)'. It is not clear which situation is primary, the Nuclear Turkic one or the Chuvash one, so we treat **yɨl* and **ya:ʎ* as technical synonyms.

Proto-Mongolic. For Proto-Mongolic, the following ternary opposition can be reconstructed which is relatively stably retained in the attested languages: **hon* 'year (as a time period)' (Nugteren 2011: 359; Gruntov & Mazo 2015: 251), **ǯil* 'annual cycle, calendar year' (Nugteren 2011: 388), **nasu-n* 'year (as a unit of measure for age)' (Nugteren 2011: 452). We fill the slot with **hon*.

Proto-Tungusic. **aŋŋa* (Tsintsius 1975: 43–44; Starostin, Dybo & Mudrak 2003: 303), attested as a basic term in all four subgroups.

Middle Korean. *hʌ́y* (Starostin, Dybo & Mudrak 2003: 1274).

Proto-Japonic. **tə̀sì* (Starostin, Dybo & Mudrak 2003: 475). Attested in Japanese and Ryukyuan.

Etymological notes. Turkic **yɨl* and Japonic **tə̀sì* might be cognates, if Turkic **yɨl* < Proto-Altaic **dìlo*, whereas Japonic **tə̀sì* < Proto-Altaic suffixed **dìlo-čʰV* with Japonic **-s-* < **-lčʰ-* (Starostin, Dybo & Mudrak 2003: 475). Although details of such cluster simplification in Japonic remains unclear due to scantiness of evidence, the whole etymology could be acceptable in light of the presence of possibly the same sibilant suffix in the Tungusic and Korean cognate stems: Tungusic **dilača:* 'sun' (not a basic term), Korean **tors* ~ **torč* 'anniversary (cycle)'. On the other hand, Robbeets' (2005: 279) criticism of the whole etymology cannot be ignored: indeed Middle Korean *tors* 'anniversary (cycle)' could be a recent derivative from Middle Korean *tor* 'to rotate' (although the morphological pattern is not entirely clear), whereas Tungusic **dilača:* 'sun (*vel sim.*)' is too distant semantically.

Supplement references

Arsenyev, Vladimir K. 2008. *Russko-oročskij slovar'. Materialy po jazyku i tradicionnoj kul'ture oročej i udègejcev [Russian-Oroch-Udihe dictionary. Materials on the language and traditional culture of the Oroch and Udihe peoples]*. (Ed.) A. Kh. Girfanova & N. L. Sukhachev. Saint Petersburg: SPbGU.

Benzing, Johannes. 1955. *Die tungusischen Sprachen: Versuch einer vergleichenden Grammatik* (Akademie Der Wissenschaften Und Der Literatur in Mainz. Abhandlungen Der Geistes- Und Sozialwissenschaftlichen Klasse 11). Wiesbaden: Verlag der Akademie der Wissenschaften und der Literatur in Mainz.

Blažek, Václav. 2019. *Altaic languages: history of research, survey, classification and a sketch of comparative grammar*. Brno: Masaryk University Press.

Clauson, Gerard. 1972. *An etymological dictionary of Pre-Thirteenth-Century Turkish*. Oxford, UK: Oxford University Press.

Doerfer, Gerhard. 1978. Classification problems of Tungus. In Gerhard Doerfer & Michael Weiers (eds.), *Beiträge zur Nordasiatischen Kulturgeschichte* (Tungusica 1), 1–26. Wiesbaden: Otto Harrassowitz.

Doerfer, Gerhard. 1995. The recent development of Nostratism. *Indogermanische Forschungen* 100. 252–267.

Dybo, Anna V. 2007. *Lingvističeskie kontakty rannix tjurkov. Leksičeskij fond. Pratjurkskij period [Language contacts of the early Turkic peoples: vocabulary: Proto-Turkic epoch]*. Moscow: Vostochnaya Literatura.

Dybo, Anna V. 2013. *Ètimologičeskij slovar' tjurkskix jazykov [Etymological dictionary of the Turkic languages]. Vol. 9: Ètimologičeskij slovar' bazisnoj leksiki tjurkskix jazykov [Etymological dictionary of the Turkic basic vocabulary]*. Astana: Prosper Print.

Dybo, Anna V. & George S. Starostin. 2008. In defense of the comparative method, or the end of the Vovin controversy. *Aspects of comparative linguistics*, vol. 3, 119–258. Moscow: RSUH Publishers.

Frellesvig, Bjarke. 2010. *A history of the Japanese language*. Cambridge: Cambridge University Press.

Georg, Stefan. 2004. Unreclassifying Tungusic. In Carsten Naeher (ed.), *Proceedings of the First International Conference on Manchu-Tungus Studies (Bonn, August 28 - September 1, 2000)*, vol. 2: Trends in Tungusic and Siberian linguistics, 45–57. Wiesbaden: Harrassowitz.

Gruntov, Ilya A. & Olga M. Mazo. 2015. Klassifikacija mongol'skix jazykov po leksikostatističeskim dannym [Lexicostatistical classification of the Mongolic languages]. *Journal of Language Relationship* 13(3). 205–255.

Janhunen, Juha. 2012. The expansion of Tungusic as an ethnic and linguistic process. In Andrej L. Malchukov & Lindsay J. Whaley (eds.), *Recent advances in Tungusic linguistics* (Turcologica 89), 5–16. Wiesbaden: Harrassowitz Verlag.

Johanson, Lars. 1998. The history of Turkic. In Lars Johanson & Éva Á. Csató (eds.), *The Turkic languages* (Routledge Language Family Descriptions), 81–125. London: Routledge.

Kazama, Shinjiro. 2003. *Basic vocabulary (A) of Tungusic languages* (Endangered Languages of the Pacific Rim A2-037). Kyoto: Nakanishi.

Lee, Ki-Moon & S. Robert Ramsey. 2011. *A history of the Korean language*. Cambridge: Cambridge University Press.

Lessing, Ferdinand D. (ed.). 1960. *Mongolian-English dictionary*. Berkeley: University of California Press.

Logunova, Ekaterina. 2020. Korean. In Alexei S. Kassian (ed.), *Moscow lexical database*. http://moslex.org/languages/Korean.

Martin, Samuel E. 1961. *Dagur Mongolian grammar, texts, and lexicon* (Indiana University Publications Uralic and Altaic Series 4). Bloomington: Indiana University.

Martin, Samuel E. 1966. Lexical evidence relating Korean to Japanese. *Language* 42(2). 185. doi:10.2307/411687.

Martin, Samuel E. 1987. *The Japanese language through time* (Yale Language Series). New Haven: Yale University Press.
Martin, Samuel E. 1992. *A reference grammar of Korean: a complete guide to the grammar and history of the Korean language*. Rutland, Vt: Charles E. Tuttle.
Martin, Samuel E. 1996. *Consonant lenition in Krean and the Macro-Altaic Question*. Honolulu: University of Hawaii Press.
Norman, Jerry. 1978. *A concise Manchu-English lexicon* (Publications on Asia of the School of International Studies 32). Seattle: University of Washington Press.
Nugteren, Hans. 2011. *Mongolic phonology and the Qinghai-Gansu languages*. Utrecht: LOT.
Orlovskaya, Maria N. 1999. *Jazyk mongol'skix tekstov XIII-XIV vv. [Language of Mongolian texts of the 13-14 centuries]*. Moscow: Institut vostokovedenija RAN.
Poppe, Nicholas. 1955. *Introduction to Mongolian comparative studies*. Helsinki: Suomalais-ugrilainen Seura.
Ramsey, S. Robert. 1991. Proto-Korean and the origin of Korean accent. In William G. Boltz & Michael C. Shapiro (eds.), *Studies in the historical phonology of Asian languages* (Amsterdam Studies in the Theory and History of Linguistic Science 77), 215–238. Amsterdam: J. Benjamins Pub. Co.
Räsänen, Martti. 1957. *Materialien zur Morphologie der türkischen Sprachen* (Studia Orientalia 21). Helsinki: Societas Orientalis Fennica.
Räsänen, Martti. 1969. *Versuch eines etymologischen Wörterbuchs der Türksprachen*. Helsinki: Suomalais-Ugrilainen Seura.
Robbeets, Martine. 2005. *Is Japanese related to Korean, Tungusic, Mongolic and Turkic?* (Turcologica 64). Wiesbaden: Harrassowitz.
Robbeets, Martine. 2015. *Diachrony of verb morphology: Japanese and the Transeurasian languages* (Trends in Linguistics. Studies and Monographs 291). Berlin: Mouton de Gruyter.
Robbeets, Martine & Remco Bouckaert. 2018. Bayesian phylolinguistics reveals the internal structure of the Transeurasian family. *Journal of Language Evolution* 3(2). 145–162. doi:10.1093/jole/lzy007.
Rybatzki, Volker. 2003. Intra-Mongolic taxonomy. In Juha Janhunen (ed.), *The Mongolic languages* (Routledge Language Family Series 5), 364–390. London: Routledge.
Ryzhkov-Shukumine, Alexandre. 2020. Long vowels in Proto-Tungusic. *International Journal of Eurasian Linguistics* 2(1). 70–93. doi:10.1163/25898833-12340024.
Sevortyan, Ervand V., Liya S. Levitskaya, Anna V. Dybo, Valentin I. Rassadin, Galina F. Blagova & Dmitriy M. Nasilov. 1974–. *Ètimologičeskij slovar' tjurkskix jazykov [Etymological dictionary of the Turkic languages]*. 9 vols. Moscow: Nauka.
Skvortsov, Mikhail I. (ed.). 1985. *Čuvašsko-russkij slovar' [Chuvash-Russian dictionary]*. 2nd edn. Moscow: Russkij Jazyk.
Starostin, Sergei A. 1991. *Altajskaja problema i proisxoždenie japonskogo jazyka [The Altaic problem and the origins of the Japanese language]*. Moscow: Nauka.
Starostin, Sergei A., Anna V. Dybo & Oleg A. Mudrak. 2003. *Etymological dictionary of the Altaic languages* (Handbook of Oriental Studies = Handbuch Der Orientalistik. Section Eight, Central Asia 8). 3 vols. Leiden: Brill.
Sunik, Orest P. 1997. Tunguso-man'čžurskie jazyki [Tungusic languages]. *Jazyki mira: Mongol'skie jazyki. Tunguso-man'čžurskie jazyki. JAponskij jazyk. Korejskij jazyk*, 153–162. Moscow: Indrik.
Tenishev, Edkhyam R. (ed.). 1988. *Sravnitel'no-istoričeskaja grammatika tjurkskix jazykov: morfologija [Comparative grammar of Turkic languages: morphology]*. Moscow: Nauka.
Tenishev, Edkhyam R. (ed.). 2001. *Sravnitel'no-istoričeskaja grammatika tjurkskix jazykov: leksika [Comparative grammar of Turkic languages: lexicon]*. 2nd edn. Moscow: Nauka.
Tenishev, Edkhyam R. & Anna V. Dybo (eds.). 2002. *Sravnitel'no-istoričeskaja grammatika tjurkskix jazykov: regional'nye rekonstrukcii [Comparative grammar of Turkic languages: regional reconstructions]*. Moscow: Nauka.
Tenishev, Edkhyam R. & Anna V. Dybo (eds.). 2006. *Sravnitel'no-istoričeskaja grammatika tjurkskix jazy-*

kov: pratjurkskij jazyk-osnova. Kartina mira pratjurkskogo ètnosa po dannym jazyka [Comparative grammar of Turkic languages: the Proto-Turkic language. A world-view of the Proto-Turkic people according to language data]. Moscow: Nauka.

Thorpe, Maner Lawton. 1983. *Ryūkyūan language history*. Los Angeles, CA: University of Southern California Unpublished PhD thesis. http://digitallibrary.usc.edu/cdm/ref/collection/p15799coll3/id/505374.

Tsintsius, Vera N. 1949. *Sravnitel'naja fonetika tunguso-man'čžurskix jazykov [Comparative phonology of the Tungusic languages]*. Leningrad: Ministerstvo prosveshcheniya RSFSR.

Tsintsius, Vera N. (ed.). 1975. *Sravnitel'nyj slovar' tunguso-man'čžurskix jazykov [Comparative dictionary of the Tungusic languages]*. Vol. 1. 2 vols. Leningrad: Nauka.

Tsintsius, Vera N. (ed.). 1977. *Sravnitel'nyj slovar' tunguso-man'čžurskix jazykov [Comparative dictionary of the Tungusic languages]*. Vol. 2. 2 vols. Leningrad: Nauka.

Vasilevich, Glafira M. 1960. K voprosu o klassifikacii tunguso-man'čžurskix jazykov [On the classification of the Tungusic languages]. *Voprosy Jazykoznanija* 2. 43–49.

Vovin, Alexander. 1993. Towards a new classification of Tungusic languages. *Eurasian Studies Yearbook* 65. 99–113.

Vovin, Alexander. 1999. Some notes on linguistic comparison. In Vitalij V. Shevoroshkin & Paul Sidwell (eds.), *Historical linguistics & lexicostatistics* (AHL Studies in the Science & History of Language 3), 67–94. Melbourne: AHL.

Vovin, Alexander. 2000. Pre-Hankul materials, Koreo-Japonic, and Altaic. *Korean Studies* 24. 142–155.

Vovin, Alexander. 2005. The end of the Altaic controversy. *Central Asiatic Journal* 49(1). 71–132.

Vovin, Alexander. 2010. *Koreo-Japonica: a re-evaluation of a common genetic origin* (Hawai‘i Studies on Korea). Honolulu: University of Hawai‘i Press.

Whaley, Lindsay J. & Sofia Oskolskaya. 2020. The classification of the Tungusic languages. In Martine Robbeets & Alexander Savelyev (eds.), *The Oxford guide to the Transeurasian languages*, 80–91. New York: Oxford University Press.

Whitman, John Bradford. 1985. *The phonological basis for the comparison of Japanese and Korean*. Cambridge: Harvard University Unpublished PhD thesis.
